# Genetic Diversity and Elite Allele Mining for Grain Traits in Rice (*Oryza sativa L.*) by Association Mapping

**DOI:** 10.3389/fpls.2016.00787

**Published:** 2016-06-07

**Authors:** Wisdom M. Edzesi, Xiaojing Dang, Lijun Liang, Erbao Liu, Imdad U. Zaid, Delin Hong

**Affiliations:** State Key Laboratory of Crop Genetics and Germplasm Enhancement, Nanjing Agricultural UniversityNanjing, China

**Keywords:** rice, grain traits, linkage disequilibrium, association mapping, elite alleles

## Abstract

Mining elite alleles for grain size and weight is of importance for the improvement of cultivated rice and selection for market demand. In this study, association mapping for grain traits was performed on a selected sample of 628 rice cultivars using 262 SSRs. Grain traits were evaluated by grain length (GL), grain width (GW), grain thickness (GT), grain length to width ratio (GL/GW), and 1000-grain weight (TGW) in 2013 and 2014. Our result showed abundant phenotypic and genetic diversities found in the studied population. In total, 2953 alleles were detected with an average of 11.3 alleles per locus. The population was divided into seven subpopulations and the levels of linkage disequilibrium (LD) ranged from 34 to 84 cM. Genome-wide association mapping detected 10 marker trait association (MTAs) loci for GL, 1MTAs locus for GW, 7 MTAs loci for GT, 3 MTAs loci for GL/GW, and 1 MTAs locus for TGW. Twenty-nine, 2, 10, 5, and 3 elite alleles were found for the GL, GW, GT, GL/GW, and TGW, respectively. Optimal cross designs were predicted for improving the target traits. The accessions containing elite alleles for grain traits mined in this study could be used for breeding rice cultivars and cloning the candidate genes.

## Introduction

Today, rice (*Oryza sativa* L.) feeds more than half the world's population, and accounts for 20% of the world's total calorie intake. Although a staple in diets worldwide, rice is central to the economy and landscape of wider East Asian, Southeast Asian, and South Asian ancient, and modern civilizations. The rapid human population growth in the world is boosting demand for a corresponding increase in crop grain yield. Developing countries account for 95% of global rice production, with China and India responsible for nearly half the world output. Nine out of the top 10 rice producing countries are in Asia and interestingly, major rice producers are also typically the major rice consumers.

Rice grain shape related traits (grain length, width, thickness, and length to-width ratio) have a direct bearing on grain weight and quality, and hence commercial success, of modern rice (*O. sativa* L.) cultivars (Redoña and MacKill, 1998). Grain yield in rice is determined by three major components: number of panicles per plant, number of grains per panicle, and grain weight. Among these, the most reliable trait is grain weight, which is measured as the 1000-grain weight (TGW). Grain size, as specified by grain length (GL), grain width (GW), grain thickness (GT), and grain length to-width ratio (GL/GW), is a major determinant of grain appearance quality and grain weight in rice.

Grain size is an important agronomic trait for artificial selection in rice breeding. Breeders tend to select plants with large seed size for high yield and appropriate grain size for milling yield and market preferences. However, it is difficult for breeders to improve grain size efficiently by phenotypes, since the traits are quantitatively inherited (McKenzie and Rutger, 1983). Rice yield is the most noticeable characteristic to farmers while the crop is in the ground, but when the product of the crop, the milled rice, reaches the market, quality becomes the key determinant of its sale-ability. Buyers, millers, and consumers judge the quality of rice on the uniformity of its size and shape as well as the pleasing appearance of its overall size-shape relationship.

Many QTLs for grain size and weight have been identified and reported by various researchers (Lin et al., 1996; Hua et al., 2002; Xing et al., 2002; Aluko et al., 2004; Li et al., 2004; Agrama et al., 2007; Song et al., 2007; Bai et al., 2010; Huang et al., 2011; Wang et al., 2011; Tran Thi et al., 2014; Dang et al., 2015). Genes that affect the grain size have been identified in inter-specific crosses (Xiao et al., 1998; Brondani et al., 2002; Ordonez et al., 2010). In most cases, wild-type alleles were associated with small grain, whereas cultivar alleles were associated with large grains. Usually, grain size is determined by GL, GW, and GT. The three traits are quantitatively inherited under the control of several or many genes. To date, 5 key genes controlling grain size have been isolated in rice: *GS*3, *GW*2, *qSW*5 or *GW*5, *GIF1*, and *GS5* (Fan et al., 2006; Shomura et al., 2008; Li et al., 2011). *GS*3 has a major effect on GL, whereas *qSW5/GW5* and *GW2* confer both the GW and weight in rice. *GIF1* encodes a cell-wall invertase that is required for carbon partitioning during early grain filling, and the over-expression of *GIF1* by using its native promoter leads to large grains (Wang et al., 2008). Shomura et al. (2008) found that a deletion in *qSW*5 was associated with grain size owing to an increase in the cell number in the outer glume of the rice spikelet.

A number of QTL studies showed that one genomic region was associated with several traits, especially yield component traits, indicating linkage and/or pleiotropic effects (Xiao et al., 1996; Tan et al., 2000; Yu et al., 2005; Tian et al., 2006; Liu et al., 2015). Recent advent of molecular and computational tools now enables the estimation of genetic diversity and population structure of rice germplasm rather easily. With the growing availability of genome sequence data and advances in technology for rapid identification and scoring of genetic markers, linkage disequilibrium (LD) based genome-wide association study (GWAS) has gained favor in higher plants, especially crops, for the mapping of genetic factors responsible for trait variation (Remington et al., 2001; Gupta et al., 2005; Mackay and Powell, 2007; Cockram et al., 2008; Sneller et al., 2009; Atwells et al., 2010; Zhou and Stephens, 2012). Providing the intrinsic nature of exploiting historical recombination events, association mapping offers increased mapping resolution to polymorphisms at sequence level and should therefore enhance the efficiency of gene discovery and facilitate marker assisted selection (MAS) in plant breeding (Moose and Mumm, 2008; Zhang et al., 2010). Once the plant cultivars are genotyped with high-density markers, association mapping is promising in resolving the genetic basis of traits of both economic and ecological importance.

The objectives of this study were (1) to evaluate the population structure and genetic diversity of a set of rice materials; (2) to detect the extent of LD between pairs of SSR markers on genome-wide scale in the population used; and (3) to detect QTLs controlling seed grain components traits and mine elite alleles; (4) to explore design of parental combinations for cultivar improvement.

## Materials and methods

### Sample collection

A total of 628 rice accessions were used from China (507) and Vietnam (121) in this study (Supplementary Table S1). Five hundred and seven varieties originated from different regions of China and have been widely used as parents in plant breeding during the past decades. The seeds of all accessions were collected, stored, and supplied by State Key Laboratory of Crop Genetics and Germplasm Enhancement, Nanjing Agricultural University, Nanjing, China.

### Phenotypic data collection

For field studies, 628 rice accessions were planted out in a field at the Jiangpu Experimental Station, Nanjing Agricultural University, China (118.62°E, 32.07°N) from early May to November in 2013 and 2014 using a randomized complete block design with two replications. For all varieties, seedlings aged about 30 days were transplanted onto the field at a spacing of 20 cm between rows and 17 cm between each individual with standard agronomic management. The middle five plants in the central row of each plot were sampled to examine grain size traits. Grain size traits contained grain length (GL), grain width (GW), grain thickness (GT), ratio of grain length to grain width (GL/GW), and 1000-grain weight (TGW), were evaluated. For each trait, means of the replicates were used in the data analyses.

### SSR marker genotyping

**Figure 1:**
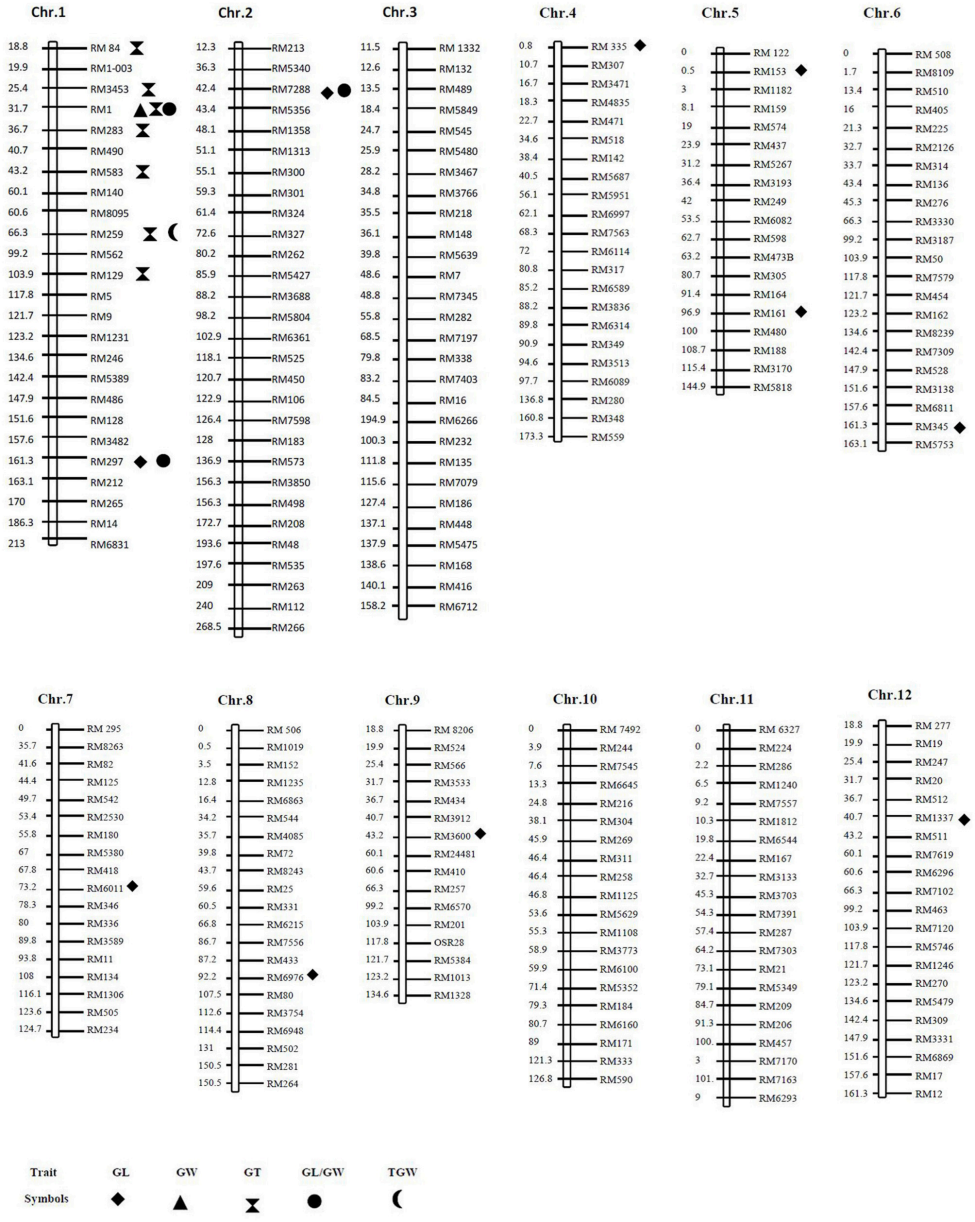
**Graphical genotypes (GGT) of all the 262 markers and their corresponding chromosome positions (measured in cM) showing marker- traits associations**.

Genomic DNA was extracted from leaf tissue of each selected plant according to the methods described by Murray and Thompson (1980). According to the published rice molecular map and microsatellite database of Temnykh et al. (2000) and McCouch et al. (2002), 262 polymorphic microsatellite markers, approximately evenly distributed scattered on 12 chromosomes were used for genotyping (Figure 1). Primers were synthesized by Shanghai Generay Biotech Co. Ltd., Shanghai, China. Each 10 μl PCR reaction consisted of 10 mM tris HCl (PH 9.0), 50 mM KCl, 0.1% triton X-100, 1.5 mM MgCl_2_, 0.5 nM dNTPs, 0.14 pM forward primers, 0.14 pM reverse primers, 0.5 U of taq polymerase, and 20 ng of genomic DNA. DNA amplification was performed using a PTC-100™ Peltier Thermal Cycler (MJ Research™ Incorporated, USA) under the following conditions: (1) denaturation at 94°C for 5 min; (2) 34 cycles of denaturation at 94°C for 0.5 min, annealing at 55–61°C for 1 min, and extension at 72°C for 1 min; (3) final extension at 72°C for 10 min. The PCR products were run on 8% polyacrylamide gel at 150 V for 1 h, and visualized using silver staining.

### Heritability

Analysis of variance (ANOVA) was carried out to determine genotypic and environmental variances among the traits measured using the SAS package (SAS Institute Inc., CARY, NC, USA). Heritability in the broad sense (H${}_{\text{B}}^{2}$) was computed on the basis of the natural population through analysis of variance using the formula H${}_{\text{B}}^{2}=$ σ${}_{\text{g}}^{2}$/(σ${}_{\text{g}}^{2}$ + σ${}_{\text{e}}^{2}$/*n*), where σ${}_{\text{g}}^{2}$ is genetic variance, σ${}_{\text{e}}^{2}$ is error variance, and *n* is number of replicates.

### Genetic diversity, phylogenetic analysis, and population structure

The summary statistics including the number of alleles per locus, major allele frequency, gene diversity, and polymorphism information content (PIC) values were determined using PowerMarker version 3.25 (Liu and Muse, 2005). Nei's distance (Nei et al., 1983) was calculated and used for the unrooted phylogeny reconstruction using neighbor joining method as implemented in PowerMarker with the tree viewed using MEGA 4.0 (Tamura et al., 2007).

Analysis of population structure among rice accessions was performed using the software package STRUCTURE in its revised version 2.2 (Falush et al., 2007). The optimum number of populations (K) was selected after five independent runs of a burn-in of 50,000 iterations followed by 100,000 iterations for each value of *K* (testing from *K* = 2 to *K* = 10). A model based clustering algorithm was applied that identified subgroups with distinctive allele frequencies and placed individuals into *K* clusters, where *K* is chosen in advance but can be varied for independent runs of the algorithm. The most likely number of clusters (*K*) was selected by comparing the logarithmized probabilities of data [Pr (X|K)] and a value for each value of *K* according to Pritchard et al. (2000). The software SPAGeDi55 (Spatial Pattern Analysis of Genetic Diversity) was used to calculate the pairwise relatedness coefficients (K, kinship matrix) to estimate the genetic relatedness among individuals.

### Linkage disequilibrium

Linkage disequilibrium (LD) was evaluated for each pair of SSR loci using TASSEL 2.1, both on all accessions and on the clusters as inferred by STRUCTURE. D′ measures modified for multiple loci were used. Significance (*P*-values) of D′ for each SSR pair was determined by 100,000 permutations. To compare phenotypes of the seven groups identified by STRUCTURE, ANOVA was employed with the SAS program version 8 (SAS Institute Inc., Cary, NC), followed by multiple means comparisons among these groups.

### Association mapping

To avoid possible spurious associations, the mixed linear model (Q+K) built in TASSEL V2.1 (Excoffier et al., 2005; Yu and Buckler, 2006; Bradbury et al., 2007) was used to account for population structure and relatedness of individuals among 628 rice accessions. Association between marker alleles and grain size component traits and weight data were performed (trait analysis by association, and linkage) taking into account gross level population structure (Q) (Bradbury et al., 2007). The *P*-value determined whether a marker is associated with the trait and the *R*^2^ indicated the fraction of the total variation explained by the marker identified. Using the association locus identified, the “null allele” (non-amplified allele) was used to determine the phenotypic effects of other alleles (Breseghello and Sorrells, 2006). The formula used for calculating phenotypic effect of a single allele was a_i_ = ∑x_ij_/n_i_ − ∑N_k_/n_k_, where a_i_ was the phenotypic effect of the allele of i; x_ij_ denoted the phenotypic measurement values of j variety carrying the allele of i; n_i_ represented the number of materials carrying the allele of i; N_k_ meant the phenotypic value of the variety of k carrying the null allele; and n_k_ represented the number of materials for the null allele. If we want to increase the trait value of interest, we have to use alleles with positive effect as the elite allele likewise, if we want to reduce the trait, we use allele of negative effect as the elite allele. In our study, marker loci with PVE >5% were considered for further analysis. Top 30 varieties with higher phenotypic values were analyzed together with the selected marker loci to determine elite alleles and their carrier varieties.

## Results

### Phenotypic evaluations

Mean value, range, coefficient of variation, kurtosis, and skewness for each trait measured in 628 accessions were shown in Table 1. The phenotypic data of the GW, GT, GL/GW, and TGW followed a normal distribution but GL followed a skewed distribution based on the skewness values and kurtosis. A two-way analysis of variance (ANOVA) showed that differences among cultivars for each trait were highly significant (*P* < 0.01), indicating a large amount of genetic variation existed in the population.

**Table 1:** **Phenotypic characteristics for grain traits in 628 rice accessions**.

**Trait**	**Year**	**Mean ± SD^a^**	**Range**	**CV^b^(%)**	**Kurtosis**	**Skewness**	**H${}_{\text{B}}^{2}$^c^(%)**
Grain length (mm)	2013	7.80 ± 1.04	6.50–12.53	13.33	4.06	1.93	96.66
	2014	7.83 ± 1.06	6.50–12.61	13.50	3.94	1.92	94.99
Grain width (mm)	2013	3.05 ± 0.41	1.97–3.94	13.30	−0.14	−0.90	88.78
	2014	3.08 ± 0.41	1.98–4.00	13.58	−0.17	−0.87	94.99
Grain thickness (mm)	2013	2.15 ± 0.18	1.53–2.58	8.44	0.01	−0.43	88.24
	2014	2.16 ± 0.18	1.53–2.59	8.37	−0.02	−0.45	95.84
Grain length to width ratio	2013	2.66 ± 0.79	1.83–6.36	29.67	3.61	1.91	98.36
	2014	2.63 ± 0.78	1.80–6.31	30.00	3.59	−1.90	97.99
1000-grain weight (g)	2013	24.35 ± 3.10	12.06–37.57	12.74	1.12	−0.22	93.50
	2014	24.41 ± 3.12	11.95–37.46	12.80	1.08	−0.22	97.99

a SD, standard deviation.

b CV, coefficient of variation.

c H${}_{\text{B}}^{2}$, heritability in the broadsense.

There existed variances between 2013 and 2014 for the five grain components traits studied (Supplementary Tables S2–S6). The means of GL over the 628 accessions were 7.80 and 7.83 mm with 96.7 and 98.8 % of H${}_{\text{B}}^{2}$ in 2013 and 2014 respectively (Table 1). GW had means of 3.05 and 3.08 mm, with 88.78 and 94.99% of H${}_{\text{B}}^{2}$ in 2013 and 2014, respectively. The means of GT over the 628 accessions were 2.15 and 2.16 mm, with 88.24 and 95.84% of H${}_{\text{B}}^{2}$ in 2013 and 2014, respectively. GL/GW had means of 2.66 and 2.63 with 93.50 and 97.99% of H${}_{\text{B}}^{2}$ in 2013 and 2014, respectively. The biggest values for TGW were 37.59 and 37.46 g in 2013 and 2014, respectively.

Data in Table 2 showed that GL was negatively correlated with GW, GT, and positively correlated with GL/GW but shows no correlation with TGW when α = 0.01. GW was positively correlated with GT and TGW but negatively correlated with GL/GW. Again, GT also shows positive correlation with TGW but negatively correlated to GL/GW. TGW was negatively correlated to GL/GW.

**Table 2:** **Correlation coefficient among five grain component traits in 2013 and 2014**.

	**GL**	**GW**	**GT**	**GL/GW**	**TGW**
GL		−0.726^**^	−0.510^**^	0.931^**^	−0.003
GW	−0.732^**^		0.678^**^	−0.910^**^	0.319^**^
GT	−0.484^**^	0.637^**^		−0.631^**^	0.606^**^
GL/GW	0.933^**^	−0.910^**^	−0.599^**^		−0.181^*^
TGW	0.006	0.286^**^	0.570^**^	−0.160^*^	

The bottom diagonal is the correlation coefficient in 2014 and the upper diagonal is the correlation coefficient in 2013. The asterisk (^*^ and ^**^) shows significant level at 0.05 and 0.01 respectively.

### Genetic diversity

**Figure 2:**
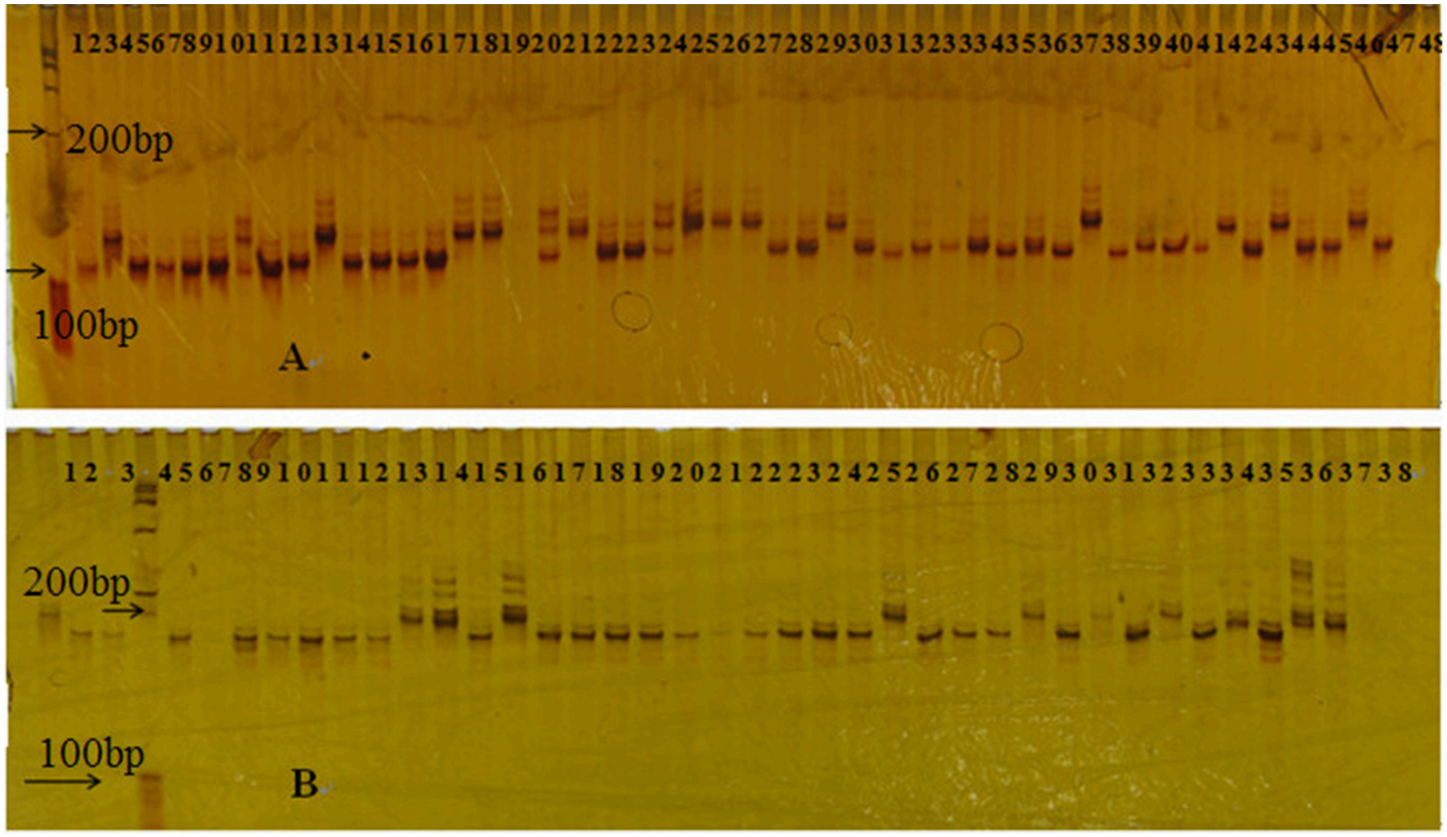
**Gel picture display profile results of SSR markers (A, RM471), and (B, RM208), amplified by some rice materials**.

A total of 262 SSR markers, randomly distributed across the genome, were used to evaluate the genetic diversity of the population. All of the 262 SSR markers were polymorphic across the 628 rice accessions and a total of 2953 alleles were detected (Supplementary Tables S7, S8). Numbers of alleles ranged from 3 (at locus RM7163_chr11) to 25 (RM7545_chr10) with an average of 11.3 alleles per locus (Figure 2, Supplementary Table S8). The genetic diversity averaged 0.7620 ranging from 0.2543 (RM82_chr7) to 0.9406 (RM7545_chr10; Supplementary Table S8). The PIC had a mean of 0.7365 ranging from 0.2425 (RM82_chr7) to 0.9373 (RM7545_chr10) with a major distribution between 0.5225 and 0.9021 (Supplementary Table S8). Two hundred and twenty-six markers (89.6%) were highly informative (PIC > 0.5), 22 (8%) moderately informative (0.5 > PIC > 0.25), and 2 (0.75%) slightly informative (PIC < 0.25).

### Population structure and genetic relatedness

**Figure 3:**
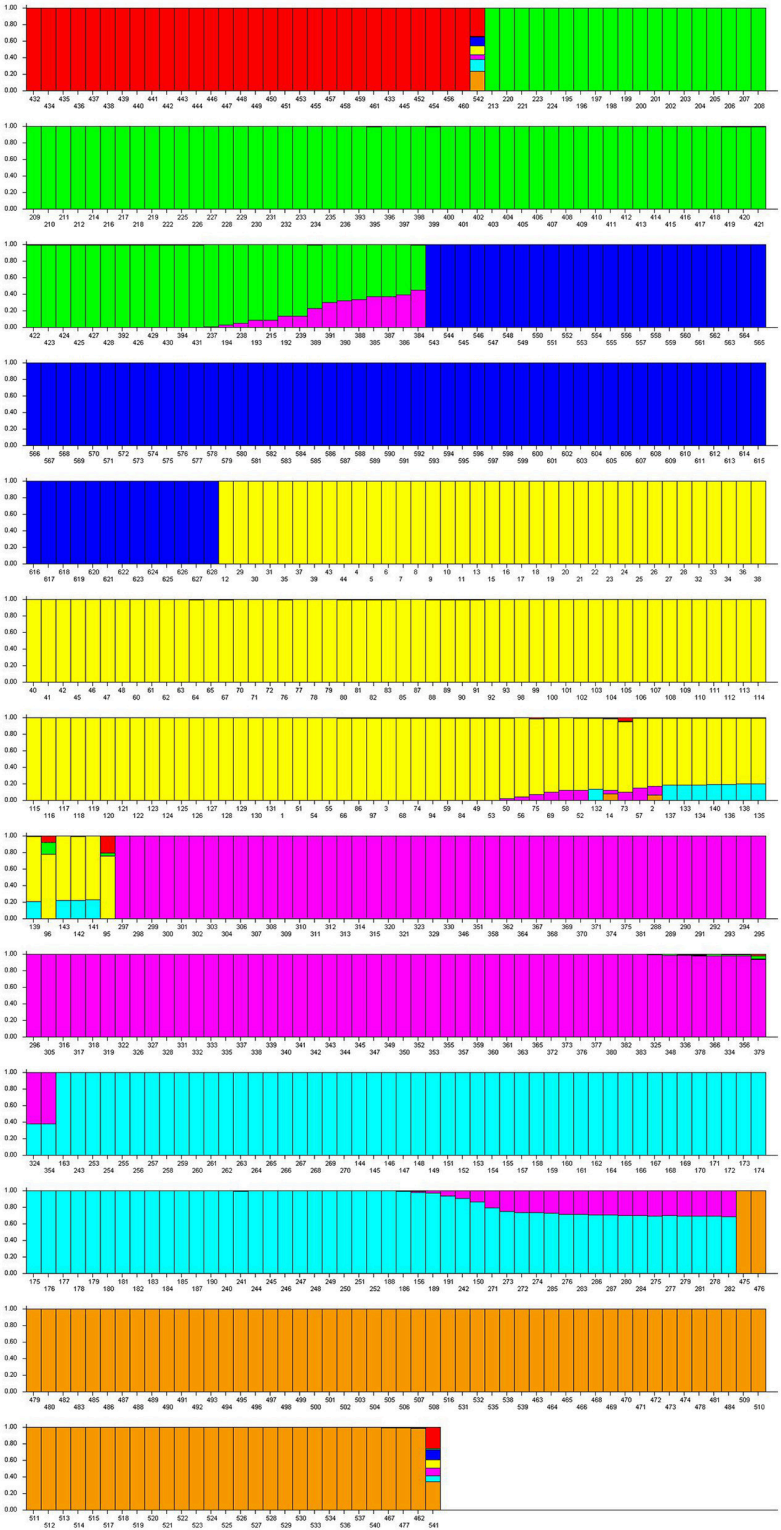
**Model based population structure of all 628 rice variety belonging to seven subpopulations predefined by STRUCRURE software**. Each accession is represented by a vertical bar. The colored subsections within each vertical bar indicate membership coefficient (Q) of the accession to different clusters. Identified subpopulations are POP1 (red color), POP2 (green color), POP3 (navy blue color), POP4 (yellow color), POP5 (purple color), POP6 (light blue color), POP7 (brown color).

**Figure 4:**
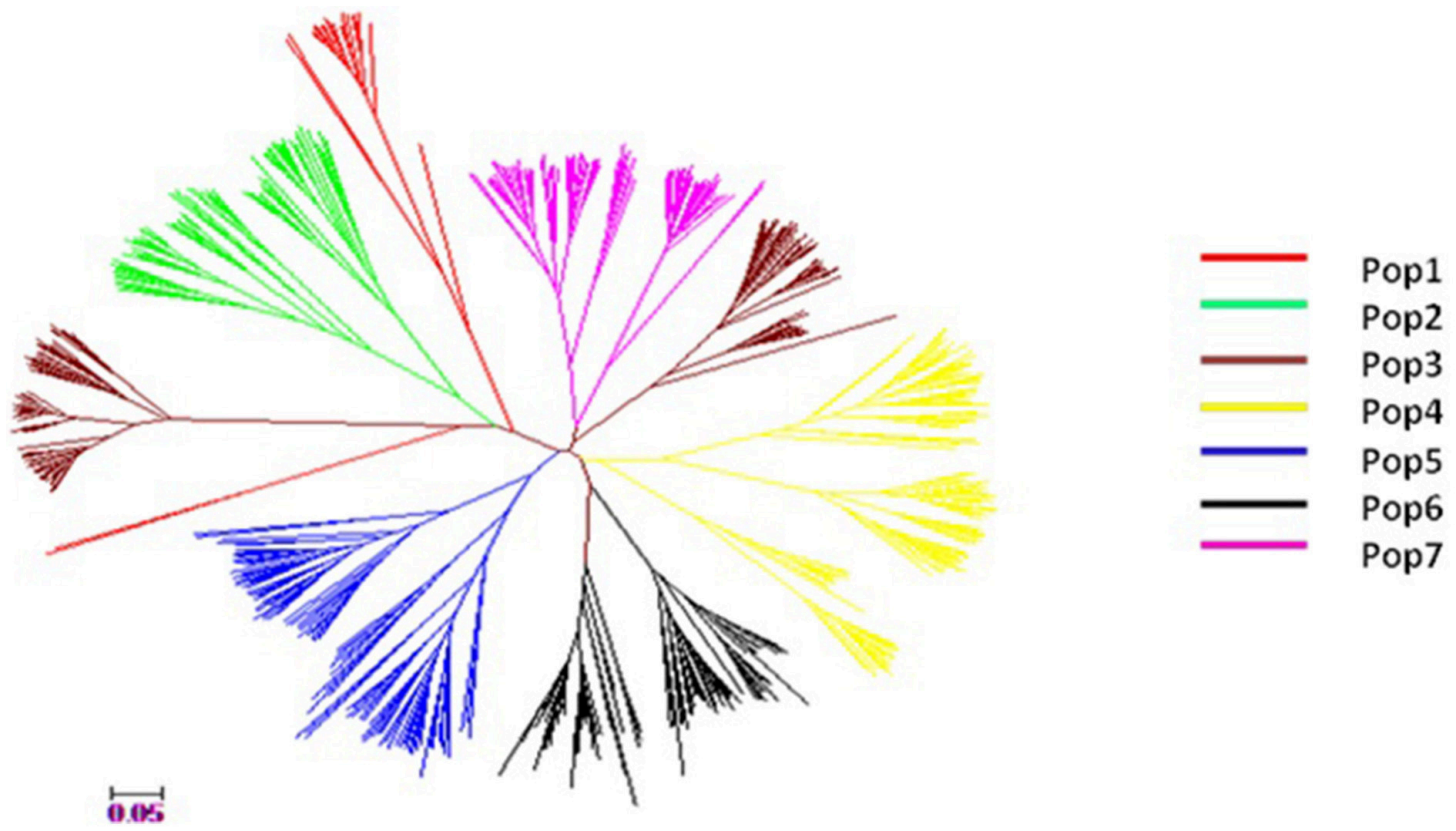
**A neighbor- joining tree for 628 rice accessions using Nei's et al. (1983) genetic distance**.

The model-based simulation of population structure using SSRs showed that the log-likelihood increased with the elevation of model parameter K, so the statistic ΔK was used to determine a suitable value for *K*. Here, the Δ*K*-value was much higher for the model parameter *K* = 7 than for other values of *K*. Population structure data based on the Q matrix for each accession are summarized in Supplementary Table S1, and the 628 accessions could be divided into seven subpopulations, viz. from POP1 to POP7 (Figure 3). A neighbor-joining tree of 628 accessions was constructed based on Nei's genetic distance and the information revealed was consistent with the result from STRUCTURE analysis (Figure 4). For instance, the accessions from Vietnam were mainly clustered into POP2 while the accessions which were landraces coming from Taihu lake valley were in POP3.

**Figure 5:**
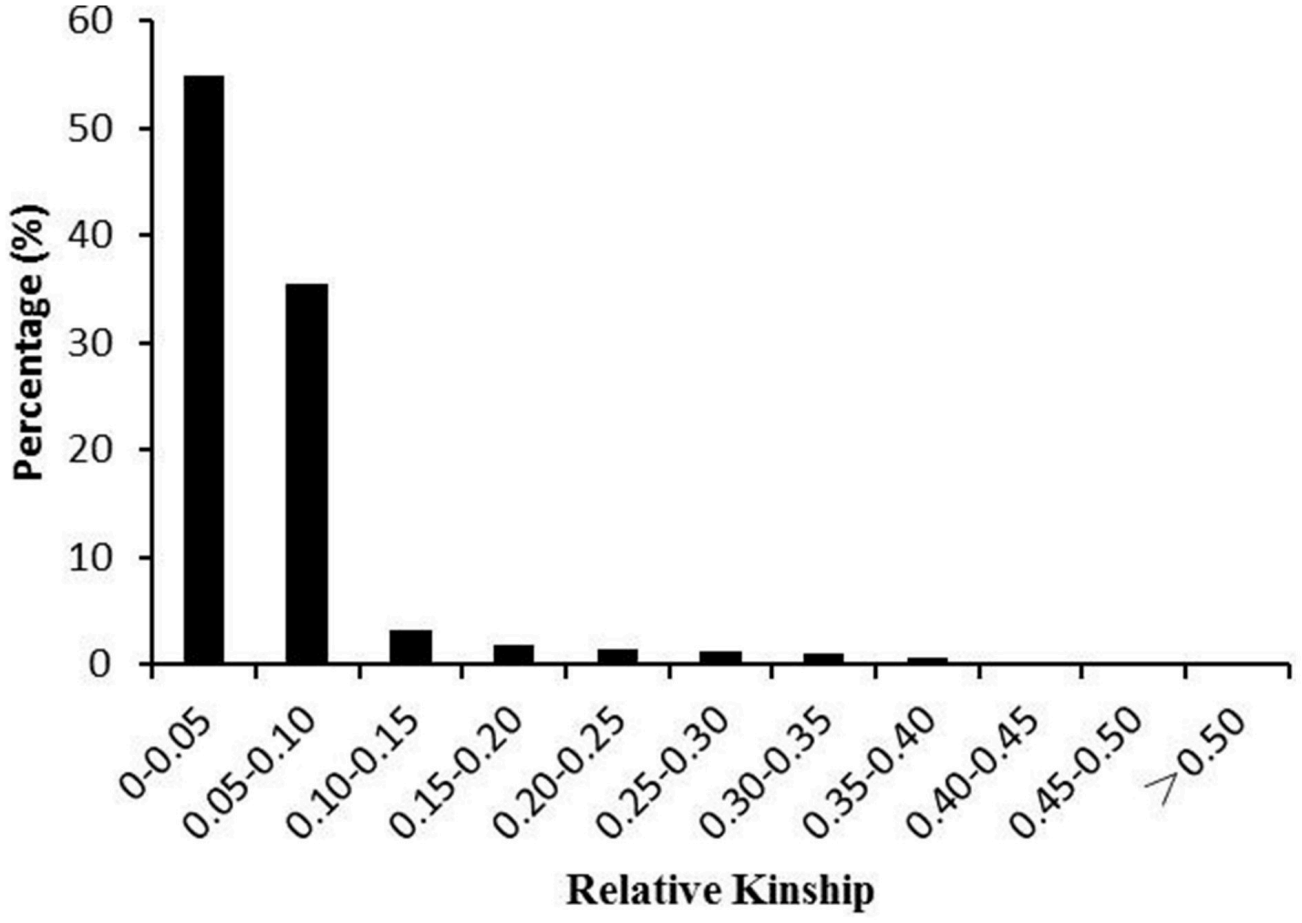
**Distribution of pair-wise kinship coefficients among 628 rice accessions based on 262 SSR markers**.

Genetic relatedness analysis in this study based on 262 SSR markers showed that more than 50% of the kinship coefficient values were < 0.05 (Figure 5), 35.6% were in a range of 0.05–0.10, and the remaining 9.5% showed various degrees of genetic relatedness, indicating that there was no or weak relatedness between pair-wise accessions used. Based on the results of the relatedness analysis, a K matrix was constructed for the association analysis.

### Linkage disequilibrium

Among the seven subpopulations, the lowest percentage of significant pair-wise loci in LD was found in POP3 (4.4%), and the highest was found in POP4 (29.3%; Table 3). POP1 had the highest average of D′-value among the subpopulation (0.64) and POP7 had the lowest average of D′- value among the seven subpopulations (0.52), suggesting that accessions in this subpopulation might have been subjected to extreme artificial selection.

**Table 3:** **Comparison of D′-values for pairwise SSR loci in each subpopulation**.

**Cluster**	**No. of LD^a^ locus pairs**	**Ratio^b^(%)**	**Frequency of D′^c^-value (*P* < 0.05)**					**Means of D′**
			**0–0.2**	**0.2–0.4**	**0.4–0.6**	**0.6–0.8**	**0.8–1.0**	
POP1	362	7	19	117	43	37	146	0.64
POP2	468	9.1	13	121	181	84	69	0.54
POP3	225	4.4	25	33	52	60	55	0.59
POP4	1507	29.3	53	277	578	436	163	0.55
POP5	804	15.6	23	185	282	102	212	0.59
POP6	992	19.3	90	83	348	326	145	0.57
POP7	791	15.4	119	91	314	180	87	0.52

a LD means linkage disequilibrium.

b Ratio between the number of significant LD locus pairs and total number of LD locus pairs.

c D′ means standardized disequilibrium coefficients.

**Figure 6:**
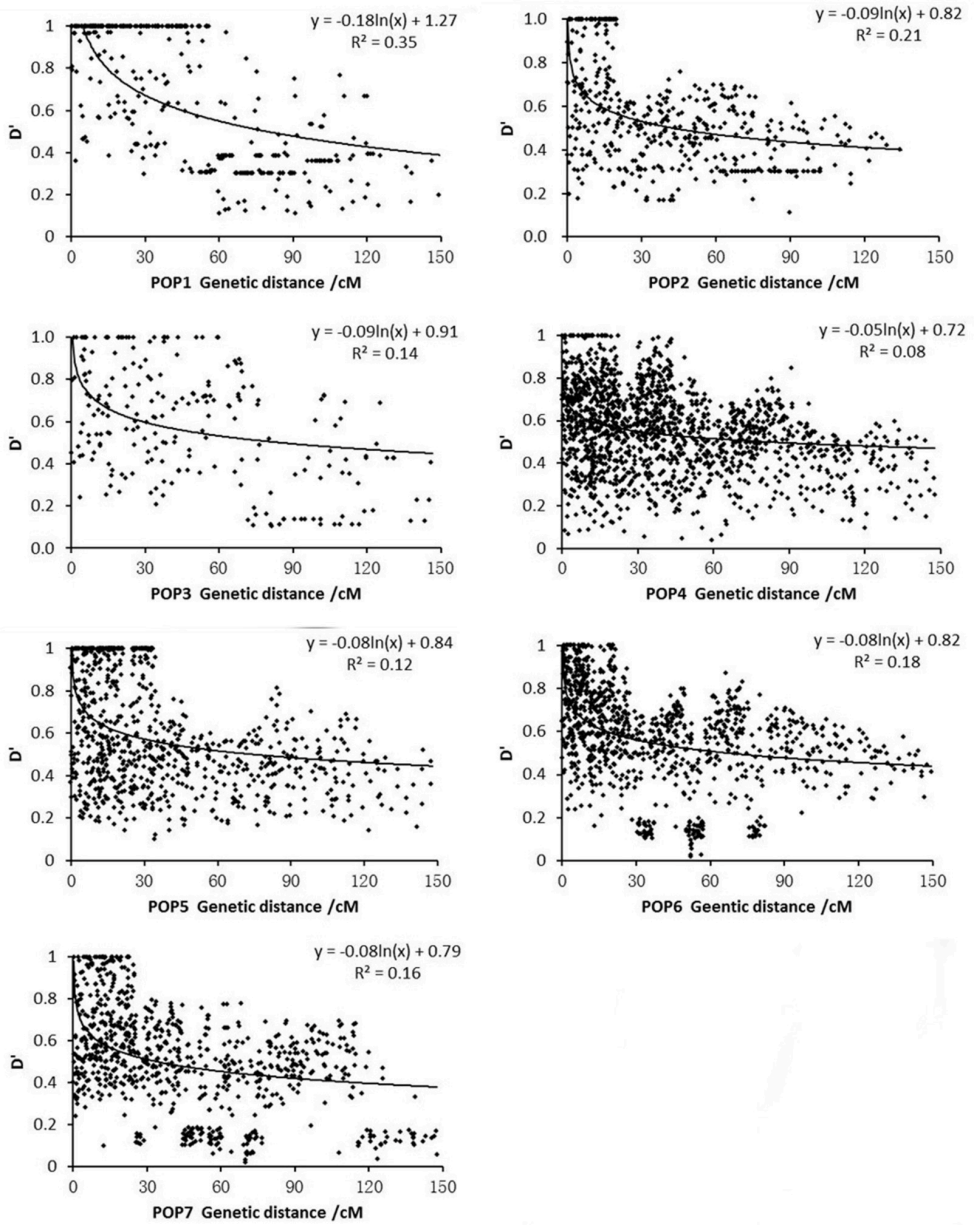
**Relationship between *D*′-value and genetic distance of syntenic marker pairs in subpopulations**.

Regression analysis between the D′-value and genetic distance of syntenic (intra-chromosome) marker pairs shown that the seven subpopulation genomes fitted in the equation y = blnx + c (Figure 6). The minimum distance of LD decay for POP1 to POP7 was 79.39, 42.82, 83.67, 78.26, 73.70, 67.48, and 34.35 cM, respectively. It was realized that POP7 had the lowest decay velocity, while POP3 demonstrated the fastest decay velocity among the seven subpopulations.

### Association analysis

Marker–trait associations were detected using MLM for five grain traits across 2 years. Association analysis identified marker–trait associations (*P* < 0.05) for all the traits evaluated (Supplementary Table S9). Table 4 showed the significant association loci with the PVE more than 5% for five grain traits across 2 years. Ten markers located on all the 12 chromosomes except chromosome 3, 10, and 11 were associated with GL. The range of phenotypic variation explained (PVE) was from 5.03 to 21.97%. RM297_Chr 1, which resides on 161.3 cM, had the maximum PVE for GL, which was 21.97% in 2013 and 21.89% in 2014 (Table 4). One marker was found distributed on chromosome 1 associated with GW, of which RM1_Chr 1 had PVE, from 6.1 to 8.3% (Table 4). Seven markers distributed on chromosome 1 were found associated with GT, of which RM84 _chr1 explained the maximum phenotypic variation, viz 19.16% in 2013 and 23.46% in 2014, respectively (Table 4). Three markers associated with GL/GW distributed on chromosomes 1 and 2 with the corresponding PVE range from 5.21 to 19.37% of which RM297_chr1 had the highest PVE of 19.37% in 2013 and 18.47% in 2014 explaining the maximum phenotypic variation (Table 4). One marker RM259 associated with TGW distributed on chromosome 1 and the PVE was from 5.40% in 2013 and 5.88% in 2014. RM259_chr1, reside on 66.3 cM on the short arm of chromosome 1 (Table 4).

**Table 4:** **Marker-trait associations with *P* < 0.05, proportion of phenotypic variance explained (PVE), marker position on chromosome derived from 262 markers and 628 rice accessions**.

**Trait**	**Marker**	**Chr. No**	**Position (cM)**	**2013**		**2014**	
				***P*-value**	**PVE (%)**	***P*-value**	**PVE (%)**
GL/mm	RM297	1	161.3	1.19E−03	21.97	1.26E−03	21.89
	RM7288	2	42.4	4.47E−02	6.37	3.93E−02	6.47
	RM335	4	0.8	7.77E−05	7.92	8.38E−05	7.88
	RM153	5	0.5	8.67E−04	6.43	8.26E−04	6.45
	RM161	5	96.9	1.49E−02	5.36	1.47E−02	5.37
	RM345	6	140.6	3.98E−05	5.22	4.03E−05	5.22
	RM6011	7	73.2	6.53E−03	17.38	6.32E−03	17.42
	RM6976	8	92.2	3.91E−04	8.42	4.09E−04	8.40
	RM3600	9	62.7	1.16E−04	6.75	1.19E−04	6.74
	RM1337	12	0.0	2.04E−03	5.05	2.16E−03	5.03
GW/mm	RM1	1	31.7	5.28E−04	8.33	4.75E−03	6.05
GT/mm	RM84	1	18.8	2.16E−04	19.16	1.03E−06	23.46
	RM3453	1	25.4	3.64E−04	9.18	5.73E−05	10.16
	RM1	1	31.7	8.12E−04	5.09	5.07E−04	5.23
	RM283	1	36.7	2.37E−04	5.76	1.92E−05	4.87
	RM583	1	43.2	2.56E−03	5.68	9.50E−04	5.20
	RM259	1	66.3	1.54E−03	6.50	8.22E−04	4.66
	RM129	1	103.9	1.35E−02	15.72	1.27E−02	16.31
GL/GW	RM1	1	31.7	2.03E−02	5.28	2.33E−04	7.36
	RM297	1	161.3	5.35E−04	19.37	2.28E−03	18.47
	RM7288	2	42.4	2.88E−03	8.30	2.24E−02	5.21
TGW/g	RM259	1	66.3	7.04E−03	5.40	3.08E−02	5.88

### Mining elite alleles

In this study, the alleles with positive effects are considered elite alleles for all five grain traits measured. Supplementary Table S10 shows a summary of elite alleles of the significant association loci with PVE more than 5% and their typical carrier materials for the given traits. The total numbers of elite alleles for GL, GW, GT, GL/GW, and TGW detected across the entire population were 29, 2, 10, 5, and 3, respectively. The allele RM3600–120 bp showed the largest phenotypic effect (1.81 mm) for GL, and the typical carrier accession was Yuedao 62 (Supplementary Table S10). The allele RM1-90 bp showed the largest phenotypic effect (0.20 mm) for GW, and the typical carrier accession was Wumangzaodao (Supplementary Table S10). The allele RM3453-135 bp showed the largest phenotypic effect (0.31 mm) for GT, and the typical carrier accession was Zhen9424 (Supplementary Table S10). The allele RM1-170 bp showed the largest phenotypic effect (0.92) for GL/GW, and the typical carrier accession was Yuedao100 (Supplementary Table S10). The allele RM259-185 bp showed the largest phenotypic effect (0.74 g) for TGW, and the typical carrier accession was Yuedao86 (Supplementary Table S10).

### Excellent cross design for novel parental combination

**Figure 7:**
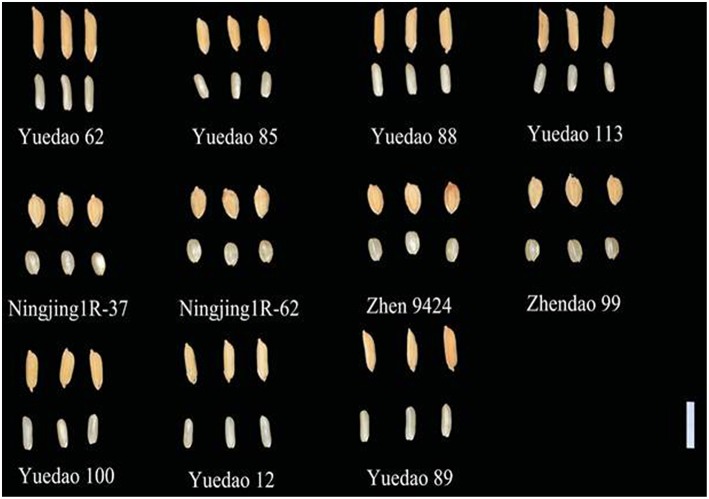
**Unhulled grains (above) and brown rice (down) of the elite parents for improving of the grain traits**. Bar, 10 mm.

Based on the number of positive alleles that could be pyramided into an individual plant and the expected phenotypic effects, the best five cross combinations for improving GL, GT, and GL/GW were proposed (Table 5). The elite alleles carried by the parents in excellent crosses were listed in Supplementary Table S11. These are super parents or varieties carrying elite alleles which are potential for cross breeding purpose. Alleles identified in these materials will help in the cross breeding programme for grain size improvement. Figure 7 shows parents in their superior crosses. Yuedao 62 and Ningjing1R-37 were found repeatedly in the supposed parental combinations which demonstrate their excellent possession of elite alleles.

**Table 5:** **Parental combinations, numbers of elite alleles, and phenotypic effects after combinations predicted from association mapping of grain length, grain thickness, and grain length to width ratio**.

**Trait**	**Parental Combination**	**No. of elite alleles predicted**	**Predicted phenotypic effect**
Grain length (mm)	Yuedao 62 × Yuedao 85	9	2.18 mm
	Yuedao 62 × Yuedao 88	8	2.22 mm
	Yuedao 62 × Yuedao 113	8	2.21 mm
Grain thickness (mm)	Ningjing1R-37 × Zhen9424	5	0.50 mm
	Ningjing1R-61 × Zhen 9424	5	0.41 mm
	Ningjing1R-37 × Zhendao 99	5	0.41 mm
Grain length to width ratio	Yuedao 100 × Yuedao12	2	1.16
	Yuedao 100 × Yuedao 89	2	2.1
	Yuedao 12 × Yuedao89	2	2.1

## Discussion

Population structure is a strong confounding factor in association studies, especially with respect to traits that are important in local adaptation or diversifying selection and familial relatedness associated with recent co-ancestry (Nordborg and Weigel, 2008). Here, we made use of the MLM approach, which took into account population structure and familial relatedness, in order to reveal the association between SSR makers and five seed grain traits in rice. Such genome-wide association mapping should, therefore, be valid even in a self-pollinating species such as rice with high levels of population structure and much familial relatedness. By use of Structure software, population consisted of 628 accessions was clustered into seven subpopulations, i.e., POP1, POP2, POP3, POP4, POP5, POP6, and POP7. One common discovery about each subpopulation is that the accessions with the same geographic origin were classed into the same group. It was revealed that POP6 had accessions coming from Vietnam likewise POP1 also revealed accession coming from the Northeastern part of China. This helps us to conclude that the result of the groupings by structure analysis was essentially consistent with the geographical region. General agreement between the genetic and predefined clusters suggests that knowledge of the ancestral background can facilitate choices of parental lines in rice-breeding programs. Also, the sample size used in this study is larger than used in the previous studies in rice (Garris et al., 2005; Agrama et al., 2007; Mather et al., 2007; Rakshit et al., 2007; Thomson et al., 2007). A larger sample size increases detection power and allows the quantification of the effects of more alleles.

Linkage disequilibrium is the basis of association analysis (Flint-Garcia et al., 2003). In comparison to other populations, the levels of LD for POP2 and POP5 in this study (42.82 and 34.35 cM, respectively), we observed a substantial drop in LD values between 20 and 40 cM, suggesting it should, nevertheless, be possible to achieve resolution down to the 25 cM level. The same observation on LD at larger distances was found in Arabidopsis (Nordborg et al., 2002) and (Kraakman et al., 2004). Many factors affect LD (Ardlie et al., 2002), but the most probable cause of the high level of LD in rice is that it is a self-pollinated species. Selection can also increase LD, for instance, by a hitchhiking effect in which the alleles at flanking loci of a locus under selection can be rapidly swept to high frequency or fixation (Kraakman et al., 2004). These studies suggest that the extent of LD varies among different genomic regions (Mather et al., 2007), different rice accessions studied (Agrama and Eizenga, 2008) and different markers used. Thus, in large populations like this kind of autogamous species, the stretches of LD extending over several cM are expected. Again, on the basis of the LD decay range in this present study, genome-wide LD mapping is possible using this set of rice materials.

In this study, we identified twenty-two markers (with PVE more than 5%) associated with grain traits using the entire set of accessions, including 10 associated with GL, 1 associated with GW, 7 associated with GT, 3 associated with GL/GW, and 1 associated with TGW. Seven of the 22 associations were in regions where the QTL associated with the given traits had been identified (http://www.gramene.org/), and they are listed in Supplementary Table S12. Fifteen loci in this study were found for the first time, including 4 for GL, 1 for GW, 7 for GT, and 3 for GL/GW. For the 4 new loci in GL, RM297_chr 1 had the largest PVE (21.97% in 2013 and 21.89% in 2014). The marker RM1, which is located on chromosome 1, was a new locus associated with GW. For the 7 new loci in GT, RM84_chr 1 had the largest PVE (19.16% in 2013 and 23.46% in 2014). For the 3 new loci in GL/GW, RM297_chr 1 had the largest PVE (19.37% in 2013 and 18.47% in 2014). These results might increase the QTL information for the grain traits and provide help for further fine mapping and cloning.

For GL trait, heritability in the broad sense averaged across 2 years was 95.8%. Among the ten SSR associated markers detected for GL, RM297_chr1 had the largest PVE (21.97% in 2013 and 21.89% in 2014). RM297-145 bp had the largest phenotypic effect value (0.22 mm) among positive elite alleles found on their marker loci. And the typical carrier material was Tongjing 109. We expect GL could be improved by the crosses listed in Table 5 which shows a potential cross breeding of super parents based on the number of alleles that could be pyramided into an individual plant and the expected phenotypic effects for improving seed grain size component traits and predicted alleles outcomes.

For GW trait, heritability in the broad sense averaged across 2 years was 91.9%. One SSR associated marker detected for GW, RM1_chr1 had PVE of 8.33% in 2013 and 6.05% in 2014. RM1-90 bp had the highest phenotypic effect value (0.20 mm) among the positive alleles found on the locus (Supplementary Table S10).

For GT trait, heritability in the broad sense averaged across 2 years was 92%. Among the seven SSR associated markers detected for GT, RM84_chr1 had the largest PVE (19.16% in 2013 and 23.46% in 2014). RM3453-135 bp had the largest phenotypic effect value (0.31 mm) among positive elite alleles found on their marker loci and the typical carrier material was Zhen9424. We expect GT could be improved by the crosses listed in Table 5.

For GL/GW trait, heritability in the broad sense average across 2 years was 98.2%. Among the three SSR associated marker detected for GL/GW, RM297_chr1 had the largest phenotypic effect value (19.37% in 2013 and 18.47%in 2014). RM297-145 bp had the highest phenotypic effect value (0.19) among the positive elite alleles found on the loci. This elite allele was carried by four accessions, in which Zijianjingnuo was the typical carrier material. GL/GW trait could be improved by the crosses listed in Table 5.

Finally on TGW trait, heritability in the broad sense average across 2 years was 95.7%. One SSR associated marker detected for TGW, RM259_chr1 had phenotypic effect value of 5.40% in 2013 and 5.88% in 2014. RM259-185 bp had the highest phenotypic effect value (0.74 g) among the positive alleles found on the locus (Supplementary Table S10).

Genetic relatedness analysis based on 262 SSR markers showed that more than 50% of the kinship coefficient values were < 0.05, 35.6% were in a range of 0.05–0.10, and the remaining 9.5% showed various degrees of genetic relatedness (Figure 6), indicating that there was no or weak relatedness between pair-wise accessions used in this study.

In conclusion, association mapping provides a powerful tool in unraveling the complex traits in plants and helps to identify superior alleles through marker-assisted selection in plant breeding. This provides essential clues that can be used to improve rice breeding programs.

## Author contributions

DH planned and designed the research; WE, XD, LL, EB, and IZ carried out the field experiment; WE, XD, LL, and EB carried out the molecular experiment; WE and XD analyzed data; WE and XD wrote the manuscript; DH revised the manuscript. WE and XD contributed equally to this work. All authors read and approved the manuscript.

### Conflict of interest statement

The authors declare that the research was conducted in the absence of any commercial or financial relationships that could be construed as a potential conflict of interest.

## References

[B1] Agrama H. A. Eizenga G. C. (2008). Molecular diversity and genome-wide linkage disequilibrium patterns in a worldwide collection of *Oryza sativa* and its wild relatives. Euphytica 160, 339–355. 10.1007/s10681-007-9535-y

[B2] Agrama H. A. Eizenga G. C. Yan W. (2007). Association mapping of yield and its components in rice cultivars. Mol. Breed. 19, 341–356. 10.1007/s11032-006-9066-6

[B3] Aluko G. Martinez C. Tohme J. Castano C. Bergman C. Oard J. H. (2004). QTL mapping of grain quality traits from the interspecific cross *Oryza sativa* × *O. glaberrima*. Theor. Appl. Genet. 109, 630–639. 10.1007/s00122-004-1668-y 15105992

[B4] Ardlie K. G. Kruglyak L. Seielstad M. (2002). Patterns of linkage disequilibrium in the human genome. Nat. Rev. Genet. 3, 299–309. 10.1038/nrg777 11967554

[B5] Atwells S. Huang Y. S. Vilhjaslmsson B. J. Willems G. Horton M. Li Y.. (2010). Genome-wide association study of 107 phenotypes in Arabidopsis thaliana inbred lines. Nature 465, 627–631. 10.1038/nature08800 20336072 PMC3023908

[B6] Bai X. F. Luo L. Yan W. Kovi M. R. Wei Z. Xing Y. (2010). Genetic dissection of rice grain shape using a recombinant inbred line population derived from two contrasting parents and fine mapping a pleiotropic quantitative trait locus qGL7. BMC Genet. 11:16. 10.1186/1471-2156-11-16 20184774 PMC2846863

[B7] Bradbury P. J. Zhang Z. Kroon D. E. Casstevens T. M. Ramdoss Y. Buckler E. S. (2007). TASSEL: software for association mapping of complex traits in diverse samples. Bioinformatics 2, 2633–2635. 10.1093/bioinformatics/btm308 17586829

[B8] Breseghello F. Sorrells M. E. (2006). Association mapping of kernel size and milling quality in wheat (*Triticum aestivum* L.) cultivars. Genetics 172, 1165–1177. 10.1534/genetics.105.044586 16079235 PMC1456215

[B9] Brondani C. Rangel P. Brondani R. Ferreira M. E. (2002). QTL mapping and introgression of yield-related traits from Oryza glumaepatula to cultivated rice (*Oryza sativa*) using microsatellite markers. Theor. Appl. Genet. 104, 1192–1203. 10.1007/s00122-002-0869-5 12582630

[B10] Cockram J. White J. Leigh F. J. Lea V. J. Chiapparino E. Laurie D. A.. (2008). Association mapping of partitioning loci in barley. BMC Genet. 9:16. 10.1186/1471-2156-9-16 18282287 PMC2276512

[B11] Dang X. J. Thi T. Edzesi W. M. Liang L. Liu Q. Liu E.. (2015). Population genetic structure of *Oryza sativa* in East and Southeast Asia and the discovery of elite alleles for grain traits. Sci. Rep. 5:11254. 10.1038/srep11254 26059752 PMC4462027

[B12] Excoffier L. Laval G. Chneider S. (2005). Arlequin ver. 3.0: an integrated software package for population genetics data analysis. Evol. Bioinform. 1, 47–50. PMC2658868 19325852

[B13] Falush D. Stephens M. Pritchard J. K. (2007). Inference of population structure using multilocus genotype data: dorminant markers and null alleles. Mol. Ecol. Notes 7, 574–578. 10.1111/j.1471-8286.2007.01758.x 18784791 PMC1974779

[B14] Fan C. Xing Y. Mao H. Lu T. Han B. Xu C. (2006). *GS3*, a major QTL for grain length and weight and minor QTL for grain width and thickness in rice, encodes a putative transmembrane protein. Theor. Appl. Genet. 112, 1164–1171. 10.1007/s00122-006-0218-1 16453132

[B15] Flint-Garcia S. Thornsberry J. Buckler E. S. (2003). Structure of linkage disequilibrium in plants. Annu. Rev. Plant Biol. 54, 357–374. 10.1146/annurev.arplant.54.031902.134907 14502995

[B16] Garris A. J. Tai T. H. Jason C. Steve K. Susan M. C. (2005). Genetic structure and diversity in *Oryza sativa* L. Genetics 169, 1631–1638. 10.1534/genetics.104.035642 15654106 PMC1449546

[B17] Gupta P. Rustgi S. Kulwal P. (2005). Linkage disequilibrium and association studies in higher plants: present status and future prospects. Plant Mol. Biol. 57, 461–485. 10.1007/s11103-005-0257-z 15821975

[B18] Hua J. P. Xing Y. Z. Xu C. G. Sun X. L. Yu S. B. Zhang Q. F. (2002). Genetic dissection of an elite rice hybrid revealed that heterozygotes are not always advantageous for performance. Genetics 162, 1885–1895. 12524357 10.1093/genetics/162.4.1885 PMC1462368

[B19] Huang X. H. Zhao Y. Wei X. Li C. Wang A. Zhao Q.. (2011). Genome-wide association study of flowering time and grain yield traits in a worldwide collection of rice germplasm. Nat. Genet. 44, 32–41. 10.1038/ng.1018 22138690

[B20] Kraakman A. Niks R. E. Petra M. Berg V. Piet S. Eeuwijk F. (2004). Linkage disequilibrium mapping of yield and yield stability in modern spring barley cultivars. Genetics 168, 435–446. 10.1534/genetics.104.026831 15454555 PMC1448125

[B21] Li J. Xiao J. Grandillo S. Jiang L. Wan Y. Deng Q.. (2004). QTL detection for rice grain quality traits using an interspecific backcross population derived from cultivated Asian (*O. sativa* L.) and African (*O. glaberrima* S.) rice. Genome 47, 697–704. 10.1139/g04-029 15284874

[B22] Li Y. B. Fan C. C. Xing Y. Z. Jiang Y. H. Luo L. J. Sun L.. (2011). Natural variation in *GS5* plays an important role in regulating grain size and yield in rice. Nat. Genet. 43, 1266–1269. 10.1038/ng.977 22019783

[B23] Lin H. X. Qian H. R. Zhuang J. Y. Lu J. Min S. K. Xiong Z. M.. (1996). RFLP mapping of QTLs for yield and related characters in rice (*Oryza sativa* L.). Theor. Appl. Genet. 92, 920–927. 10.1007/BF00224031 24166618

[B24] Liu E. Liu X. Zeng S. Zhao K. M. Zhu C. F. Liu Y.. (2015). Time-course association mapping of the grain-filling rate in rice (*Oryza sativa* L.). PLOS ONE 10:e0119959. 10.1371/journal.pone.0119959 25789629 PMC4366047

[B25] Liu K. Muse S. V. (2005). PowerMarker: integrated analysis environment for genetic marker data. Bioinformatics 21, 2128–2129. 10.1093/bioinformatics/bti282 15705655

[B26] Mackay I. Powell W. (2007). Methods for linkage disequilibrium mapping in crops. Trends Plant Sci. 12, 57–63. 10.1016/j.tplants.2006.12.001 17224302

[B27] Mather K. A. Caicedo A. L. Polato N. R. Olsen K. M. Susan M. C. Purugganan M. D. (2007). The extent of linkage disequilibrium in rice (*Oryza sativa* L.). Genetics 177, 2223–2232. 10.1534/genetics.107.079616 17947413 PMC2219496

[B28] McCouch S. R. Teytelman L. Xu Y. B. Lobos K. B. Clare K. Walton M.. (2002). Development and mapping of 2240 new SSR markers for rice (*Oryza sativa* L.). DNA Res. 9, 199–207. 10.1093/dnares/9.6.199 12597276

[B29] McKenzie K. S. Rutger J. N. (1983). Genetic analysis of amylose content, alkali spreading score, and grain dimensions in rice. Crop Sci. 23, 306–313. 10.2135/cropsci1983.0011183X002300020031x

[B30] Moose S. P. Mumm R. H. (2008). Molecular plant breeding as the foundation for 21st century crop improvement. Plant Physiol. 147, 969–977. 10.1104/pp.108.118232 18612074 PMC2442525

[B31] Murray M. G. Thompson W. F. (1980). Rapid isolation of high-molecular-weight-plant DNA. Nucleic Acids Res. 8, 4321–4325. 10.1093/nar/8.19.4321 7433111 PMC324241

[B32] Nei M. Tajima F. A. Tateno Y. (1983). Accuracy of estimated phylogenetic trees from molecular data. J. Mol. Evol. 19, 153–170. 10.1007/BF02300753 6571220

[B33] Nordborg M. Borevitz J. O. Bergelson J. Berry C. C. Chory J. Hagenblad J.. (2002). The extent of linkage disequilibrium in Arabidopsis thaliana. Nat. Genet. 30, 190–193. 10.1038/ng813 11780140

[B34] Nordborg M. Weigel D. (2008). Next-generation genetics in plants. Nature 456, 720–723. 10.1038/nature07629 19079047

[B35] Ordonez J. S. Silva J. Oard J. H. (2010). Association mapping of grain quality and flowering time in elite japonica rice germplasm. J. Cereal Sci. 51, 337–343. 10.1016/j.jcs.2010.02.001

[B36] Pritchard J. K. Stephens M. Donnelly P. (2000). Donnelly inference of population structure using multilocus genotype data. Genetics 155, 945–959. 10835412 10.1093/genetics/155.2.945 PMC1461096

[B37] Rakshit S. Rakshit A. Matsumura H. Takahashi Y. Hasegawa Y. Ito A.. (2007). Large-scale DNA polymorphism study of *Oryza sativa* and *O. rufipogon* reveals the origin and divergence of Asian rice. Theor. Appl. Genet. 114, 731–743. 10.1007/s00122-006-0473-1 17219210

[B38] Redoña E. D. MacKill D. J. (1998). Quantitative trait locus analysis for rice panicle and grain characteristics. Theor. Appl. Genet. 96, 957–963.

[B39] Remington D. L. Thornsberry J. M. Matsuoka Y. Wilson L. M. Whitt S. R. Doebley J.. (2001). Structure of linkage disequilibrium and phenotypic associations in the maize genome. Proc. Natl. Acad. Sci. U.S.A. 98, 11479–11484. 10.1073/pnas.201394398 11562485 PMC58755

[B40] Shomura A. Izawa T. Ebana K. Ebitania T. Kanega H. Konishi S.. (2008). Deletion in a gene associated with grain size increased yields during rice domestication. Nat. Genet. 40, 1023–1028. 10.1038/ng.169 18604208

[B41] Sneller C. H. Mather D. E. Crepieux S. (2009). Analytical approaches and population types for finding and utilizing QTL in complex plant populations. Crop Sci. 49, 363–380. 10.2135/cropsci2008.07.0420

[B42] Song X. J. Huang W. Shi M. Zhu M. Z. Lin H. X. (2007). A QTL for rice grain width and weight encodes a previously unknown RING-type E3 ubiquitin ligase. Nat. Genet. 39, 623–630. 10.1038/ng2014 17417637

[B43] Tamura K. Dudley J. Nei M. Kumar S. (2007). MEGA 4: Molecular Evolutionary Genetics Analysis (MEGA) software version 4.0. Mol. Biol. Evol. 24, 1596–1599. 10.1093/molbev/msm092 17488738

[B44] Tan Y. F. Xing Y. Z. Li J. X. Yu S. B. Xu C. G. Zhang Q. (2000). Genetic bases of appearance quality of rice grains in shanyou 63, an elite rice hybrid. Theor. Appl. Genet. 101, 823–829. 10.1007/s001220051549 22665200

[B45] Temnykh S. Park W. D. Ayres N. Cartinhour S. Hauck N. Lipovich L. (2000). Mapping and genome organization of microsatellite sequence in rice (*Oryza sativa* L.). Theor. Appl. Genet. 100, 697–712. 10.1007/s001220051342

[B46] Thomson M. J. Septiningsih E. M. Suwardjo F. Santoso T. J. Silitonga T. S. McCouch S. R. (2007). Genetic diversity analysis of traditional and improved Indonesian rice (*Oryza sativa* L.) germplasm using microsatellite markers. Theor. Appl. Genet. 114, 559–568. 10.1007/s00122-006-0457-1 17136372

[B47] Tian F. Zhu Z. Zhang B. Tan L. Fu Y. Wang X.. (2006). Fine mapping of a quantitative trait locus for grain number per panicle from wild rice (*Oryza rufipogon* Griff.). Theor. Appl. Genet. 113, 619–629. 10.1007/s00122-006-0326-y 16770601

[B48] Tran Thi T. G. Dang X. J. Liu Q. M. Zhao K. M. Wang H. Hong D. L. (2014). Association analysis of rice grian traits with SSR markers. Chin. J. Rice Sci. 28, 243–257. 10.3969/j.issn.1001-7216.2014.03.004

[B49] Wang J. McClean P. Lee R. Goos R. Helms T. (2008). Association mapping of iron deficiency chlorosis loci soybean (*Glycine max* L. Merr.) advanced breeding lines. Theor. Appl. Genet. 116, 777–787. 10.1007/s00122-008-0710-x 18292984

[B50] Wang L. Wang A. H. Huang X. H. Zhao Q. Dong G. J. Qian Q. (2011). Mapping 49 quantitative trait loci at high resolution through sequencing-based genotyping of rice recombinant inbred lines. Theor. Appl. Genet. 122, 327–340. 10.1007/s00122-010-1449-8 20878143 PMC3021254

[B51] Xiao J. Li J. Grandillo S. Ahn S. N. Yuan L. Tanksley S. D.. (1998). Identification of trait-improving quantitative trait loci alleles from a wild rice relative, Oryza rufipogon. Genetics 150, 899–909. 9755218 10.1093/genetics/150.2.899 PMC1460369

[B52] Xiao J. Li J. M. Yuan L. P. Tanksley S. (1996). Identification of QTLs affecting traits of agronomic importance in a recombinant inbred population derived from a sub-specific rice cross. Theor. Appl. Genet. 92, 230–244. 10.1007/BF00223380 24166172

[B53] Xing Y. Z. Tan Y. F. Hua J. P. Sun X. L. Xu C. G. Zhang Q. F. (2002). Characterization of the main effects, epistatic effects and their environmental interactions of QTLs on the genetic basis of yield traits in rice. Theor. Appl. Genet. 105, 248–257. 10.1007/s00122-002-0952-y 12582526

[B54] Yu J. M. Buckler E. S. (2006). Genetic association mapping and genome organization of maize. Biotechnology 17, 155–160. 10.1016/j.copbio.2006.02.003 16504497

[B55] Yu J. M. Pressoir G. Briggs W. H. Bi V. Yamasaki M. Doebley J.. (2005). A unified mixed-model method for association mapping that accounts for multiple levels of relatedness. Nat. Genet. 38, 203–208. 10.1038/ng1702 16380716

[B56] Zhang Z. W. Ersoz E. Lai C. Q. Todhunter R. J. Tiwari H. K, Gore, M. A.. (2010). Mixed linear model approach adapted for genome-wide association studies. Nat. Genet. 42, 355–360. 10.1038/ng.546 20208535 PMC2931336

[B57] Zhou X. Stephens M. (2012). Genome-wide efficient mixed model analysis for association studies. Nat. Genet. 44, 821–824. 10.1038/ng.2310 22706312 PMC3386377

