# Supplementary material for: Genetic Diversity and Elite Allele Mining for Grain Traits in Rice (Oryza sativa L.) by Association Mapping
Source: Front Plant Sci. 2016 Jun 7;7:787. doi: 10.3389/fpls.2016.00787 (PMC4896222; doi:10.3389/fpls.2016.00787)
Supplement: Supplementary file 1 [file Table1.DOC]

**Supplementary table S1 Rice materials and their membership probabilities corresponding to each subpopulation**

|  | | | | | | | | | | |  |
| --- | --- | --- | --- | --- | --- | --- | --- | --- | --- | --- | --- |
| **Code** | **Accessions** | **Accession ID**  **or pedigree** | **Origin** | **Q values** | | | | | | | **Sub-pop** |
|  |  |  |  | **Q1** | **Q2** | **Q3** | **Q4** | **Q5** | **Q6** | **Q7** |  |
| W1 | Yazihuang | T045 | Jinshan, Shanghai | 0.000 | 0.000 | 0.000 | 0.998 | 0.000 | 0.000 | 0.001 | 4 |
| W2 | Hongmangzaodao | T261 | Kunshan, Jiangsu | 0.003 | 0.004 | 0.000 | 0.816 | 0.109 | 0.001 | 0.068 | 4 |
| W3 | Wanhuangdao | T815 | Wuxian, Jiangsu | 0.001 | 0.001 | 0.000 | 0.997 | 0.000 | 0.000 | 0.001 | 4 |
| W4 | Guozinuo | T680 | Kunshan, Jiangsu | 0.000 | 0.000 | 0.000 | 0.999 | 0.000 | 0.000 | 0.000 | 4 |
| W5 | Shuijingbaidao | T543 | Wuxian, Jiangsu | 0.000 | 0.000 | 0.000 | 0.999 | 0.000 | 0.000 | 0.000 | 4 |
| W6 | Wumangzaodao | T442 | Changshu, Jiangsu | 0.000 | 0.000 | 0.000 | 0.999 | 0.000 | 0.000 | 0.000 | 4 |
| W7 | Sanbailitou | T527 | Kunshan, Jiangsu | 0.000 | 0.000 | 0.000 | 0.999 | 0.000 | 0.000 | 0.000 | 4 |
| W8 | Cuyingwanyangdao | T585 | Wuxi, Jiangsu | 0.000 | 0.000 | 0.000 | 0.999 | 0.000 | 0.000 | 0.000 | 4 |
| W9 | Yanglingdao | T632 | Wuxi, Jiangsu | 0.000 | 0.000 | 0.000 | 0.999 | 0.000 | 0.000 | 0.000 | 4 |
| W10 | Wanyedao | T643 | Wuxian, Jiangsu | 0.000 | 0.000 | 0.000 | 0.999 | 0.000 | 0.000 | 0.000 | 4 |
| W11 | Qiaobinghuang | T684 | Taicang, Jiangsu | 0.000 | 0.000 | 0.000 | 0.999 | 0.000 | 0.000 | 0.000 | 4 |
| W12 | Tiejingqing | T651 | Kunshan, Jiangsu | 0.000 | 0.000 | 0.000 | 1.000 | 0.000 | 0.000 | 0.000 | 4 |
| W13 | Xiaobaiyedao | T777 | Wuxi, Jiangsu | 0.000 | 0.000 | 0.000 | 0.999 | 0.000 | 0.000 | 0.000 | 4 |
| W14 | Baoxintaihuqing | T834 | Wujiang, Jiangsu | 0.003 | 0.009 | 0.000 | 0.826 | 0.044 | 0.000 | 0.081 | 4 |
| W15 | Jiangfeng4 | T655 | Jiangyin, Jiangsu | 0.000 | 0.000 | 0.000 | 0.999 | 0.000 | 0.000 | 0.000 | 4 |
| W16 | Sujing4 | T643 | Suzhou, Jiangsu | 0.000 | 0.000 | 0.000 | 0.999 | 0.000 | 0.000 | 0.000 | 4 |
| W17 | Aizhongluohanhuang | T315 | Changshu, Jiangsu | 0.000 | 0.000 | 0.000 | 0.999 | 0.000 | 0.000 | 0.000 | 4 |
| W18 | Baodao | T100 | Wuxi, Jiangsu | 0.000 | 0.000 | 0.000 | 0.999 | 0.000 | 0.000 | 0.000 | 4 |
| W19 | Wanmuxiqiu | T101 | Taicang, Jiangsu | 0.000 | 0.000 | 0.000 | 0.999 | 0.000 | 0.000 | 0.000 | 4 |
| W20 | Huangsanshi | T528 | Wujiang, Jiangsu | 0.000 | 0.000 | 0.000 | 0.999 | 0.000 | 0.000 | 0.000 | 4 |
| W21 | Erheidao | T129 | Wuxi, Jiangsu | 0.000 | 0.000 | 0.000 | 0.999 | 0.000 | 0.000 | 0.000 | 4 |
| W22 | Xiaoqingzhong | T737 | Wuxian, Jiangsu | 0.000 | 0.000 | 0.000 | 0.999 | 0.000 | 0.000 | 0.000 | 4 |
| W23 | Zaoguangtou | T179 | Wuxi, Jiangsu | 0.000 | 0.000 | 0.000 | 0.999 | 0.000 | 0.000 | 0.000 | 4 |
| W24 | Xiaoluohanhuang | T325 | Changshu, Jiangsu | 0.000 | 0.000 | 0.000 | 0.999 | 0.000 | 0.000 | 0.000 | 4 |
| W25 | Souzhouqing | T167 | Jiangyin, Jiangsu | 0.000 | 0.000 | 0.000 | 0.999 | 0.000 | 0.000 | 0.000 | 4 |
| W26 | Wanluli | T331 | Jiangyin, Jiangsu | 0.000 | 0.000 | 0.000 | 0.999 | 0.000 | 0.000 | 0.000 | 4 |
| W27 | Wanbaguo | T357 | Jiangyin, Jiangsu | 0.000 | 0.000 | 0.000 | 0.999 | 0.000 | 0.000 | 0.000 | 4 |
| W28 | Ebusinuodao | T386 | Wuxi, Jiangsu | 0.000 | 0.000 | 0.000 | 0.999 | 0.000 | 0.000 | 0.000 | 4 |
| W29 | Laodiegu | T397 | Wujiang, Jiangsu | 0.000 | 0.000 | 0.000 | 1.000 | 0.000 | 0.000 | 0.000 | 4 |
| W30 | Yefenghuang | T402 | Wujiang, Jiangsu | 0.000 | 0.000 | 0.000 | 1.000 | 0.000 | 0.000 | 0.000 | 4 |
| W31 | Chenjiazhong | T554 | Kunshan, Jiangsu | 0.000 | 0.000 | 0.000 | 1.000 | 0.000 | 0.000 | 0.000 | 4 |
| W32 | Zaoheitouhong | T473 | Wujiang, Jiangsu | 0.000 | 0.000 | 0.000 | 0.999 | 0.000 | 0.000 | 0.000 | 4 |
| W33 | Luohanhuang | T560 | Jiangyin, Jiangsu | 0.000 | 0.000 | 0.000 | 0.999 | 0.000 | 0.000 | 0.000 | 4 |
| W34 | Longgouzhong | T580 | Qingpu, Shanghai | 0.000 | 0.000 | 0.000 | 0.999 | 0.000 | 0.000 | 0.000 | 4 |
| W35 | Shiluqing | T652 | Kunshan, Jiangsu | 0.000 | 0.000 | 0.000 | 1.000 | 0.000 | 0.000 | 0.000 | 4 |
| W36 | Ligengqing | T656 | Yixing, Jiangsu | 0.000 | 0.000 | 0.000 | 0.999 | 0.000 | 0.000 | 0.000 | 4 |
| W37 | Heitouhong | T474 | Wujiang, Jiangsu | 0.000 | 0.000 | 0.000 | 1.000 | 0.000 | 0.000 | 0.000 | 4 |
| W38 | Laolaihong | T689 | Wuxian, Jiangsu | 0.000 | 0.000 | 0.000 | 0.999 | 0.000 | 0.000 | 0.000 | 4 |
| W39 | Erlibie | T701 | Wuxian, Jiangsu | 0.000 | 0.000 | 0.000 | 1.000 | 0.000 | 0.000 | 0.000 | 4 |
| W40 | Jinguhuang | T841 | Wujiang, Jiangsu | 0.000 | 0.000 | 0.000 | 0.999 | 0.000 | 0.000 | 0.000 | 4 |
| W41 | Cuganhuangdao | T718 | Wujiang, Jiangsu | 0.000 | 0.000 | 0.000 | 0.999 | 0.000 | 0.000 | 0.000 | 4 |
| W42 | Zaoshirihuangdao | T728 | Wuxian, Jiangsu | 0.000 | 0.000 | 0.000 | 0.999 | 0.000 | 0.000 | 0.000 | 4 |
| W43 | Shengtangqing | T759 | Changshu, Jiangsu | 0.000 | 0.000 | 0.000 | 1.000 | 0.000 | 0.000 | 0.000 | 4 |
| W44 | Xiaomandao | T750 | Wujiang, Jiangsu | 0.000 | 0.000 | 0.000 | 1.000 | 0.000 | 0.000 | 0.000 | 4 |
| W45 | Shengtangdao | T206 | Changshu, Jiangsu | 0.000 | 0.000 | 0.000 | 0.999 | 0.000 | 0.000 | 0.000 | 4 |
| W46 | Wanmandao | T772 | Wujiang, Jiangsu | 0.000 | 0.000 | 0.000 | 0.999 | 0.000 | 0.000 | 0.000 | 4 |
| W47 | Nantouzhong | T600 | Kunshan, Jiangsu | 0.000 | 0.000 | 0.000 | 0.999 | 0.000 | 0.000 | 0.000 | 4 |
| W48 | Daniaodao | T453 | Changshu, Jiangsu | 0.000 | 0.000 | 0.000 | 0.999 | 0.000 | 0.000 | 0.000 | 4 |
| W49 | Kongqueqing | T833 | Kunshan, Jiangsu | 0.000 | 0.007 | 0.000 | 0.989 | 0.004 | 0.000 | 0.000 | 4 |
| W50 | Kaiqing | T232 | Kunshan, Jiangsu | 0.000 | 0.000 | 0.000 | 0.972 | 0.027 | 0.000 | 0.000 | 4 |
| W51 | Manyedao | T513 | Kunshan, Jiangsu | 0.000 | 0.000 | 0.000 | 0.998 | 0.002 | 0.000 | 0.000 | 4 |
| W52 | Baikenuo | T354 | Wujiang, Jiangsu | 0.001 | 0.000 | 0.000 | 0.874 | 0.124 | 0.000 | 0.000 | 4 |
| W53 | Baimangnuo | T033 | Wujiang, Jiangsu | 0.001 | 0.005 | 0.000 | 0.989 | 0.005 | 0.000 | 0.000 | 4 |
| W54 | Xiangzhunuo | T452 | Changshu, Jiangsu | 0.000 | 0.000 | 0.000 | 0.998 | 0.001 | 0.000 | 0.000 | 4 |
| W55 | Yaxienuo | T480 | Wuxian, Jiangsu | 0.000 | 0.000 | 0.000 | 0.998 | 0.001 | 0.000 | 0.000 | 4 |
| W56 | Xianhui429 | T573 | Nanjing, Jiangsu | 0.000 | 0.000 | 0.000 | 0.952 | 0.048 | 0.000 | 0.000 | 4 |
| W57 | Zijianxian3 | T504 | Nanjing, Jiangsu | 0.000 | 0.001 | 0.000 | 0.846 | 0.149 | 0.000 | 0.004 | 4 |
| W58 | Huangsandannuo | T106 | Wuxi, Jiangsu | 0.000 | 0.000 | 0.000 | 0.878 | 0.121 | 0.000 | 0.000 | 4 |
| W59 | Jia159 | Zheshendao2003008 | Jiaxing, Zhejiang | 0.000 | 0.000 | 0.000 | 0.996 | 0.000 | 0.000 | 0.000 | 4 |
| W60 | Sidao10 | ZD-05529 | Siyang, Jiangsu | 0.000 | 0.000 | 0.000 | 0.999 | 0.000 | 0.000 | 0.000 | 4 |
| W61 | Wuqiang | ZD-05545 | Wujin, Jiangsu | 0.000 | 0.000 | 0.000 | 0.999 | 0.000 | 0.000 | 0.000 | 4 |
| W62 | Wuyujing3 | Suzhongshenzidi156 | Wujin, Jiangsu | 0.000 | 0.000 | 0.000 | 0.999 | 0.000 | 0.000 | 0.000 | 4 |
| W63 | Xiushui04 | GS01009-1990 | Nanjing, Jiangsu | 0.000 | 0.000 | 0.000 | 0.999 | 0.000 | 0.000 | 0.000 | 4 |
| W64 | Zhendao88 | Suzhongshenzidi 265 | Zhenjiang, Jiangsu | 0.001 | 0.000 | 0.000 | 0.999 | 0.000 | 0.000 | 0.000 | 4 |
| W65 | Zhendao6 | Guoshendao990009 | Zhenjiang, Jiangsu | 0.000 | 0.000 | 0.000 | 0.999 | 0.000 | 0.000 | 0.000 | 4 |
| W66 | Taijing9 | ZD-05548 | Taibei,Tianwan | 0.001 | 0.000 | 0.000 | 0.999 | 0.000 | 0.000 | 0.000 | 4 |
| W67 | Taijing16xuanAC | ZD-05549 | Taibei,Tianwan | 0.001 | 0.000 | 0.000 | 0.999 | 0.000 | 0.000 | 0.000 | 4 |
| W68 | Taijing16xuanzi | ZD-05550 | Taibei,Tianwan | 0.001 | 0.000 | 0.000 | 0.997 | 0.001 | 0.000 | 0.001 | 4 |
| W69 | Diantun502xuanzao | 09-02598 | Kunming, Yunnan | 0.001 | 0.000 | 0.000 | 0.894 | 0.102 | 0.000 | 0.002 | 4 |
| W70 | Hongdao35 | ZD-05552 | Nanjing, Jiangsu | 0.000 | 0.000 | 0.000 | 0.999 | 0.000 | 0.000 | 0.000 | 4 |
| W71 | Hongdao37 | ZD-05553 | Nanjing, Jiangsu | 0.000 | 0.000 | 0.000 | 0.999 | 0.000 | 0.000 | 0.000 | 4 |
| W72 | Zijianjingnuo | ZD-05556 | Nanjing, Jiangsu | 0.000 | 0.000 | 0.000 | 0.999 | 0.000 | 0.000 | 0.000 | 4 |
| W73 | Nannongjing62401 | ZD-05557 | Nanjing, Jiangsu | 0.041 | 0.000 | 0.000 | 0.847 | 0.105 | 0.000 | 0.001 | 4 |
| W74 | Tongjing109 | SS201115 | Nantong, Jiangsu | 0.002 | 0.000 | 0.000 | 0.997 | 0.000 | 0.000 | 0.000 | 4 |
| W75 | Yangdao6 | Guoshendao2001002 | Yangzhou, Jiangsu | 0.011 | 0.000 | 0.000 | 0.916 | 0.069 | 0.000 | 0.003 | 4 |
| W76 | Nignjing1 | Sushendao200417 | Nanjing, Jiangsu | 0.001 | 0.000 | 0.000 | 0.999 | 0.000 | 0.000 | 0.000 | 4 |
| W77 | Wujing15 | Sushendao200418 | Wujin, Jiangsu | 0.000 | 0.000 | 0.000 | 0.999 | 0.000 | 0.000 | 0.000 | 4 |
| W78 | Wuxiangjing14 | Sushendao200315 | Wujin, Jiangsu | 0.000 | 0.000 | 0.000 | 0.999 | 0.000 | 0.000 | 0.000 | 4 |
| W79 | Xudao3 | Sushendao200306 | Xuzhou, Jiangsu | 0.000 | 0.000 | 0.000 | 0.999 | 0.000 | 0.000 | 0.000 | 4 |
| W80 | Nannongjing003 | ZD-05572 | Nanjing, Jiangsu | 0.001 | 0.000 | 0.000 | 0.999 | 0.000 | 0.000 | 0.000 | 4 |
| W81 | Nannongjing005 | ZD-05574 | Nanjing, Jiangsu | 0.001 | 0.000 | 0.000 | 0.999 | 0.000 | 0.000 | 0.000 | 4 |
| W82 | 5jing20 | T718 | Nanjing, Jiangsu | 0.001 | 0.000 | 0.000 | 0.999 | 0.000 | 0.000 | 0.000 | 4 |
| W83 | 5jing15 | ZD-05575 | Nanjing, Jiangsu | 0.001 | 0.000 | 0.000 | 0.999 | 0.000 | 0.000 | 0.000 | 4 |
| W84 | molingjing | ZD-05577 | Nanjing, Jiangsu | 0.007 | 0.000 | 0.000 | 0.999 | 0.000 | 0.000 | 0.000 | 4 |
| W85 | 5jing03 | ZD-05578 | Nanjing, Jiangsu | 0.000 | 0.000 | 0.000 | 0.999 | 0.000 | 0.000 | 0.000 | 4 |
| W86 | 5jing68 | ZD-05579 | Nanjing, Jiangsu | 0.002 | 0.000 | 0.000 | 0.999 | 0.000 | 0.000 | 0.000 | 4 |
| W87 | Xudao4 | ZD-05646 | Xuzhou, Jiangsu | 0.000 | 0.000 | 0.000 | 0.999 | 0.000 | 0.000 | 0.000 | 4 |
| W88 | Xudao5 | Guoshendao2006059 | Xuzhou, Jiangsu | 0.001 | 0.000 | 0.000 | 0.999 | 0.000 | 0.000 | 0.000 | 4 |
| W89 | Huaidao9 | SS200607 | Huaian, Jiangsu | 0.001 | 0.000 | 0.000 | 0.999 | 0.000 | 0.000 | 0.000 | 4 |
| W90 | Yandao6 | Sushendao200205 | Yancheng, Jiangsu | 0.001 | 0.000 | 0.000 | 0.999 | 0.000 | 0.000 | 0.000 | 4 |
| W91 | Yangguang200 | Guoshendao2008043 | Lianyungang, Jiangsu | 0.001 | 0.000 | 0.000 | 0.999 | 0.000 | 0.000 | 0.000 | 4 |
| W92 | Lianjing2 | Guoshendao990021 | Lianyungang, Jiangsu | 0.000 | 0.000 | 0.000 | 0.999 | 0.000 | 0.000 | 0.000 | 4 |
| W93 | Xiushui79 | Guoshendao2008021 | Jiaxing, Zhejiang | 0.000 | 0.000 | 0.000 | 0.999 | 0.000 | 0.000 | 0.000 | 4 |
| W94 | Cbao | W92010108 | Hefei, Anhui | 0.001 | 0.001 | 0.000 | 0.999 | 0.000 | 0.000 | 0.000 | 4 |
| W95 | Nipponbare | Yamabiko/Saikaze | Aichi, Japan | 0.206 | 0.035 | 0.000 | 0.758 | 0.000 | 0.000 | 0.000 | 4 |
| W96 | Zhen9424 | ZD-05658 | Zhenjiang, Jiangsu | 0.073 | 0.147 | 0.000 | 0.778 | 0.000 | 0.000 | 0.000 | 4 |
| W97 | Wuyujing7 | SZS300 | Wujin, Jiangsu | 0.000 | 0.000 | 0.000 | 0.998 | 0.000 | 0.000 | 0.000 | 4 |
| W98 | Yanfujing8 | Sushendao200608 | Yancheng, Jiangsu | 0.000 | 0.000 | 0.000 | 0.999 | 0.000 | 0.000 | 0.000 | 4 |
| W99 | Zhengdao18 | Sushendao201311 | Zhenzhou, Henan | 0.000 | 0.000 | 0.000 | 0.999 | 0.000 | 0.000 | 0.000 | 4 |
| W100 | Huaidao18 | Sushendao201505 | Huaian, Jiangsu | 0.000 | 0.000 | 0.000 | 0.999 | 0.000 | 0.000 | 0.000 | 4 |
| W101 | Shengdao16 | Guoshendao2010048 | Jiaxiang, Shandong | 0.000 | 0.000 | 0.000 | 0.999 | 0.000 | 0.000 | 0.000 | 4 |
| W102 | Shengdao14 | Lunongshen2007024 | Jiaxiang, Shandong | 0.000 | 0.000 | 0.000 | 0.999 | 0.000 | 0.000 | 0.000 | 4 |
| W103 | Yujing6 | Guoshendao980002 | Zhenzhou, Henan | 0.000 | 0.000 | 0.000 | 0.999 | 0.000 | 0.000 | 0.000 | 4 |
| W104 | Huaidao8 | Sushendao200410 | Huaian, Jiangsu | 0.000 | 0.000 | 0.000 | 0.999 | 0.000 | 0.000 | 0.000 | 4 |
| W105 | Jindao9618 | Guoshendao2008044 | Dongli, Tianjin | 0.000 | 0.000 | 0.000 | 0.999 | 0.000 | 0.000 | 0.000 | 4 |
| W106 | Wandao68 | WPS03010384 | Hefei, Anhui | 0.000 | 0.000 | 0.000 | 0.999 | 0.000 | 0.000 | 0.000 | 4 |
| W107 | Xudao2 | SS201411 | Xuzhou, Jiangsu | 0.000 | 0.000 | 0.000 | 0.999 | 0.000 | 0.000 | 0.000 | 4 |
| W108 | Sujing8 | Sushendao200612 | Suzhou, Jiangsu | 0.000 | 0.000 | 0.000 | 0.999 | 0.000 | 0.000 | 0.000 | 4 |
| W109 | Xiangjing9407 | Xiangjing1/82-1244 | Nanjing, Jiangsu | 0.000 | 0.000 | 0.000 | 0.999 | 0.000 | 0.000 | 0.000 | 4 |
| W110 | Zhongjing212 | unknown | Nanjing, Jiangsu | 0.000 | 0.000 | 0.000 | 0.999 | 0.000 | 0.000 | 0.000 | 4 |
| W111 | Zhongjing9677 | unknown | Nanjing, Jiangsu | 0.000 | 0.000 | 0.000 | 0.999 | 0.000 | 0.000 | 0.000 | 4 |
| W112 | Zhongjing131 | unknown | Nanjing, Jiangsu | 0.000 | 0.000 | 0.000 | 0.999 | 0.000 | 0.000 | 0.000 | 4 |
| W113 | Suwujing | unknown | Wujin, Jiangsu | 0.000 | 0.000 | 0.000 | 0.999 | 0.000 | 0.000 | 0.000 | 4 |
| W114 | Zhognjing438 | unknown | Nanjing, Jiangsu | 0.000 | 0.000 | 0.000 | 0.999 | 0.000 | 0.000 | 0.000 | 4 |
| W115 | Yanjing9 | SS200707 | Yancheng, Jiangsu | 0.000 | 0.000 | 0.000 | 0.999 | 0.000 | 0.000 | 0.000 | 4 |
| W116 | Yangfujing7 | Sushendao200413 | Yangzhou, Jiangsu | 0.000 | 0.000 | 0.000 | 0.999 | 0.000 | 0.000 | 0.000 | 4 |
| W117 | Zijing | 09-02587 | Nanjing, Jiangsu | 0.000 | 0.000 | 0.000 | 0.999 | 0.000 | 0.000 | 0.000 | 4 |
| W118 | Zhendao10 | SS200710 | Zhenjiang, Jiangsu | 0.000 | 0.000 | 0.000 | 0.999 | 0.000 | 0.000 | 0.000 | 4 |
| W119 | Zhenghan2 | Guoshendao2003031 | Zhengzhou, Henan | 0.000 | 0.000 | 0.000 | 0.999 | 0.000 | 0.000 | 0.000 | 4 |
| W120 | Zhenghan6 | Guoshendao2005055 | Zhengzhou, Henan | 0.000 | 0.000 | 0.000 | 0.999 | 0.000 | 0.000 | 0.000 | 4 |
| W121 | Xishihuang | T204 | Wuxian, Jiangsu | 0.000 | 0.000 | 0.000 | 0.999 | 0.000 | 0.000 | 0.000 | 4 |
| W122 | Daliangdao | T227 | Wuxi, Jiangsu | 0.000 | 0.000 | 0.000 | 0.999 | 0.000 | 0.000 | 0.000 | 4 |
| W123 | Heizuidao | T262 | Changshu, Jiangsu | 0.000 | 0.000 | 0.000 | 0.999 | 0.000 | 0.000 | 0.000 | 4 |
| W124 | Xiaohuangdao | T288 | Wuxian, Jiangsu | 0.000 | 0.000 | 0.000 | 0.999 | 0.000 | 0.000 | 0.000 | 4 |
| W125 | Fenghaungdao | T526 | Changshu, Jiangsu | 0.000 | 0.000 | 0.000 | 0.999 | 0.000 | 0.000 | 0.000 | 4 |
| W126 | Maijieqing | T75 | Songjiang, Shanghai | 0.000 | 0.000 | 0.000 | 0.999 | 0.000 | 0.000 | 0.000 | 4 |
| W127 | Jijingdao | T235 | Wujiang, Jiangsu | 0.000 | 0.000 | 0.000 | 0.999 | 0.000 | 0.000 | 0.000 | 4 |
| W128 | Zhognsuyangzhogndao | T343 | Wuxi, Jiangsu | 0.000 | 0.000 | 0.000 | 0.999 | 0.000 | 0.000 | 0.000 | 4 |
| W129 | Duiguzhong | T368 | Wujiang, Jiangsu | 0.000 | 0.000 | 0.000 | 0.999 | 0.000 | 0.000 | 0.000 | 4 |
| W130 | Shuaishaban | T629 | Songjiang, Shanghai | 0.000 | 0.000 | 0.000 | 0.999 | 0.000 | 0.000 | 0.000 | 4 |
| W131 | Niumaohuang | T167 | Taicang, Jiangsu | 0.000 | 0.000 | 0.000 | 0.999 | 0.000 | 0.000 | 0.000 | 4 |
| W132 | Wanheitouhong | T132 | Wujiang, Jiangsu | 0.000 | 0.001 | 0.000 | 0.863 | 0.000 | 0.136 | 0.000 | 4 |
| W133 | Taihuqing | T2 | Kunshan, Jiangsu | 0.000 | 0.001 | 0.000 | 0.809 | 0.000 | 0.189 | 0.000 | 4 |
| W134 | Yilimang | T583 | Changshu, Jiangsu | 0.000 | 0.002 | 0.000 | 0.807 | 0.000 | 0.190 | 0.000 | 4 |
| W135 | Wuqitou | T470 | Wujin, Jiangsu | 0.000 | 0.005 | 0.000 | 0.789 | 0.000 | 0.205 | 0.000 | 4 |
| W136 | Jiaoaiheitouhong | T136 | Wujiang, Jiangsu | 0.000 | 0.001 | 0.000 | 0.799 | 0.000 | 0.199 | 0.000 | 4 |
| W137 | Laowusi | T607 | Wujiang, Jiangsu | 0.000 | 0.002 | 0.000 | 0.812 | 0.000 | 0.186 | 0.000 | 4 |
| W138 | Manliuzhong | T386 | Jinshan, Shanghai | 0.000 | 0.001 | 0.000 | 0.795 | 0.000 | 0.203 | 0.000 | 4 |
| W139 | Tainluohuang | T200 | Changshu, Jiangsu | 0.000 | 0.002 | 0.000 | 0.789 | 0.000 | 0.209 | 0.000 | 4 |
| W140 | Jiucaiqing | T73 | Changshu, Jiangsu | 0.000 | 0.001 | 0.000 | 0.803 | 0.000 | 0.196 | 0.000 | 4 |
| W141 | Aiqidaliuzhong | T375 | Jiading, Shanghai | 0.000 | 0.000 | 0.000 | 0.770 | 0.000 | 0.229 | 0.000 | 4 |
| W142 | Lujingqing | T45 | Wujiang, Jiangsu | 0.001 | 0.000 | 0.000 | 0.772 | 0.000 | 0.227 | 0.000 | 4 |
| W143 | Gaoliangqing | T67 | Wujiang, Jiangsu | 0.000 | 0.000 | 0.000 | 0.777 | 0.000 | 0.222 | 0.000 | 4 |
| W144 | Yishixing | T562 | Changshu, Jiangsu | 0.000 | 0.000 | 0.000 | 0.000 | 0.000 | 0.999 | 0.000 | 6 |
| W145 | Heizhong | T845 | Wuxian, Jiangsu | 0.000 | 0.000 | 0.000 | 0.000 | 0.000 | 0.999 | 0.000 | 6 |
| W146 | Louhanbai | T545 | Kunshan, Jiangsu | 0.000 | 0.000 | 0.000 | 0.000 | 0.000 | 0.999 | 0.000 | 6 |
| W147 | Xueliqing | T31 | Wuxi, Jiangsu | 0.000 | 0.000 | 0.000 | 0.000 | 0.000 | 0.999 | 0.000 | 6 |
| W148 | Liyangxiaohongdao | T94 | Liyang, Jiangsu | 0.000 | 0.000 | 0.000 | 0.000 | 0.000 | 0.999 | 0.000 | 6 |
| W149 | Juhuahuang | T185 | Wuxi, Jiangsu | 0.000 | 0.000 | 0.000 | 0.000 | 0.000 | 0.999 | 0.000 | 6 |
| W150 | Changzijingyedao | T332 | Wuxian, Jiangsu | 0.000 | 0.000 | 0.000 | 0.000 | 0.130 | 0.869 | 0.000 | 6 |
| W151 | Gaidaoqing | T61 | Wujiang, Jiangsu | 0.000 | 0.000 | 0.000 | 0.000 | 0.000 | 0.999 | 0.000 | 6 |
| W152 | Dingzhuangdao | T252 | Wuxi, Jiangsu | 0.000 | 0.000 | 0.000 | 0.000 | 0.000 | 0.999 | 0.000 | 6 |
| W153 | Xuetangzhong | T351 | Jiangyin, Jiangsu | 0.000 | 0.000 | 0.000 | 0.000 | 0.000 | 0.999 | 0.000 | 6 |
| W154 | Guanbaidan | T580 | Wujiang, Jiangsu | 0.000 | 0.000 | 0.000 | 0.000 | 0.000 | 0.999 | 0.000 | 6 |
| W155 | Hongbaodao | T145 | Jiaxing, Zhejiang | 0.000 | 0.001 | 0.000 | 0.000 | 0.000 | 0.999 | 0.000 | 6 |
| W156 | Tiegandao | T272 | Wujiang, Jiangsu | 0.000 | 0.000 | 0.000 | 0.000 | 0.018 | 0.999 | 0.000 | 6 |
| W157 | Juzigaung | T608 | Wuxi, Jiangsu | 0.000 | 0.000 | 0.000 | 0.000 | 0.000 | 0.999 | 0.000 | 6 |
| W158 | Yebaidao | T321 | Taicang, Jiangsu | 0.000 | 0.000 | 0.000 | 0.000 | 0.000 | 0.999 | 0.000 | 6 |
| W159 | Daheitouhong | T137 | Wujiang, Jiangsu | 0.000 | 0.000 | 0.000 | 0.000 | 0.000 | 0.999 | 0.000 | 6 |
| W160 | Baigedao | T551 | Wuxian, Jiangsu | 0.000 | 0.000 | 0.000 | 0.000 | 0.000 | 0.999 | 0.000 | 6 |
| W161 | Diediezhong | T356 | Qingpu, Shanghai | 0.000 | 0.000 | 0.000 | 0.000 | 0.000 | 0.999 | 0.000 | 6 |
| W162 | Puxidadaotou | T482 | Wujiang, Jiangsu | 0.000 | 0.000 | 0.000 | 0.000 | 0.000 | 0.999 | 0.000 | 6 |
| W163 | Yangdao | T339 | Wujiang, Jiangsu | 0.000 | 0.000 | 0.000 | 0.000 | 0.000 | 1.000 | 0.000 | 6 |
| W164 | Yanhongdao | T144 | Wujiang, Jiangsu | 0.000 | 0.000 | 0.000 | 0.000 | 0.000 | 0.999 | 0.000 | 6 |
| W165 | Baikewandao | T275 | Wuxi, Jiangsu | 0.000 | 0.000 | 0.000 | 0.000 | 0.000 | 0.999 | 0.000 | 6 |
| W166 | Aiguodadaotou | T460 | Wujiang, Jiangsu | 0.000 | 0.000 | 0.000 | 0.000 | 0.000 | 0.999 | 0.000 | 6 |
| W167 | Sishitou | T577 | Wuxian, Jiangsu | 0.000 | 0.000 | 0.000 | 0.000 | 0.000 | 0.999 | 0.000 | 6 |
| W168 | Jiuxiaozhong | T425 | Wujiang, Jiangsu | 0.000 | 0.000 | 0.000 | 0.000 | 0.000 | 0.999 | 0.000 | 6 |
| W169 | Chushuhuang | T182 | Wuxian, Jiangsu | 0.000 | 0.000 | 0.000 | 0.000 | 0.000 | 0.999 | 0.000 | 6 |
| W170 | Qianjindao | T116 | Wujiang, Jiangsu | 0.000 | 0.000 | 0.000 | 0.000 | 0.000 | 0.999 | 0.000 | 6 |
| W171 | Qijiangqing | T50 | Kunshan, Jiangsu | 0.000 | 0.000 | 0.000 | 0.000 | 0.000 | 0.999 | 0.000 | 6 |
| W172 | Baishuqing | T72 | Qingpu, Shanghai | 0.000 | 0.000 | 0.000 | 0.000 | 0.000 | 0.999 | 0.000 | 6 |
| W173 | Feilaifeng | T519 | Wuxi, Jiangsu | 0.000 | 0.000 | 0.000 | 0.000 | 0.000 | 0.999 | 0.000 | 6 |
| W174 | Kejia6 | Y11 | Kunshan, Jiangsu | 0.000 | 0.000 | 0.000 | 0.000 | 0.000 | 0.999 | 0.000 | 6 |
| W175 | Lamujia | Y14 | Kunshan, Jiangsu | 0.000 | 0.000 | 0.000 | 0.000 | 0.000 | 0.999 | 0.000 | 6 |
| W176 | Haonuopie | Y42 | Kunshan, Jiangsu | 0.000 | 0.000 | 0.000 | 0.000 | 0.000 | 0.999 | 0.000 | 6 |
| W177 | Xiganggu | Y47 | Wujiang, Jiangsu | 0.000 | 0.000 | 0.000 | 0.000 | 0.000 | 0.999 | 0.000 | 6 |
| W178 | Shuangchengnuo | Y71 | Wujiang, Jiangsu | 0.000 | 0.000 | 0.000 | 0.000 | 0.000 | 0.999 | 0.000 | 6 |
| W179 | Qiutiandaxiedao | Y73 | Wujiang, Jiangsu | 0.000 | 0.000 | 0.000 | 0.000 | 0.000 | 0.999 | 0.000 | 6 |
| W180 | Qiyunuo10 | Y74 | Wujiang, Jiangsu | 0.000 | 0.000 | 0.000 | 0.000 | 0.000 | 0.999 | 0.000 | 6 |
| W181 | Wunuo1 | Y82 | Wujiang, Jiangsu | 0.000 | 0.000 | 0.000 | 0.000 | 0.000 | 0.999 | 0.000 | 6 |
| W182 | Jianongnuo2 | Y103 | Wujiang, Jiangsu | 0.000 | 0.000 | 0.000 | 0.000 | 0.000 | 0.999 | 0.000 | 6 |
| W183 | Hongnong5 | Y129 | Wujiang, Jiangsu | 0.000 | 0.000 | 0.000 | 0.000 | 0.000 | 0.999 | 0.000 | 6 |
| W184 | Nonglinnuo4 | Y142 | Wujiang, Jiangsu | 0.000 | 0.000 | 0.000 | 0.000 | 0.000 | 0.999 | 0.000 | 6 |
| W185 | Xiangnuodao | Y143 | Wuxian, Jiangsu | 0.000 | 0.000 | 0.000 | 0.000 | 0.000 | 0.999 | 0.000 | 6 |
| W186 | Luchaihong | Y149 | Wujiang, Jiangsu | 0.000 | 0.000 | 0.000 | 0.000 | 0.004 | 0.995 | 0.000 | 6 |
| W187 | Cungu | Y164 | Wujiang, Jiangsu | 0.000 | 0.000 | 0.000 | 0.000 | 0.000 | 0.999 | 0.000 | 6 |
| W188 | Katena | Y165 | Wujiang, Jiangsu | 0.000 | 0.000 | 0.000 | 0.000 | 0.000 | 0.998 | 0.000 | 6 |
| W189 | Guanchanuo | Y167 | Wujiang, Jiangsu | 0.000 | 0.000 | 0.000 | 0.000 | 0.028 | 0.971 | 0.000 | 6 |
| W190 | Kuihuanuo | Y185 | Wuxian, Jiangsu | 0.000 | 0.000 | 0.000 | 0.000 | 0.000 | 0.999 | 0.000 | 6 |
| W191 | Suyunuo | Y226 | Wuxian, Jiangsu | 0.000 | 0.000 | 0.000 | 0.000 | 0.059 | 0.939 | 0.001 | 6 |
| W192 | Hongjiaozhan | Y297 | Wuxian, Jiangsu | 0.000 | 0.859 | 0.000 | 0.001 | 0.137 | 0.002 | 0.001 | 2 |
| W193 | Haobuka | Y317 | Wuxian, Jiangsu | 0.000 | 0.911 | 0.000 | 0.000 | 0.088 | 0.000 | 0.000 | 2 |
| W194 | Chuyanghan32 | Y341 | Wuxian, Jiangsu | 0.000 | 0.971 | 0.000 | 0.000 | 0.028 | 0.000 | 0.000 | 2 |
| W195 | Libanyi | Y343 | Wuxian, Jiangsu | 0.000 | 0.999 | 0.000 | 0.000 | 0.000 | 0.000 | 0.000 | 2 |
| W196 | Kunnong8 | T649 | Kunshan, Jiangsu | 0.000 | 0.999 | 0.000 | 0.000 | 0.000 | 0.000 | 0.000 | 2 |
| W197 | Guihuahuang | T641 | Nanjing, Jiangsu | 0.000 | 0.999 | 0.000 | 0.000 | 0.000 | 0.000 | 0.000 | 2 |
| W198 | Zhoujiazhong | T687 | Wujiang, Jiangsu | 0.000 | 0.999 | 0.000 | 0.000 | 0.000 | 0.000 | 0.000 | 2 |
| W199 | Xiaofenghuang | T529 | Wuxian, Jiangsu | 0.000 | 0.999 | 0.000 | 0.000 | 0.000 | 0.000 | 0.000 | 2 |
| W200 | Xiangjingdao | T724 | Wuxian, Jiangsu | 0.000 | 0.999 | 0.000 | 0.000 | 0.000 | 0.000 | 0.000 | 2 |
| W201 | Huizao | T591 | Wujiang, Jiangsu | 0.000 | 0.999 | 0.000 | 0.000 | 0.000 | 0.000 | 0.000 | 2 |
| W202 | Yingtoudao | T270 | Kunshan, Jiangsu | 0.000 | 0.999 | 0.000 | 0.000 | 0.000 | 0.000 | 0.000 | 2 |
| W203 | Changdaotou | T465 | Wujiang, Jiangsu | 0.000 | 0.999 | 0.000 | 0.000 | 0.000 | 0.000 | 0.000 | 2 |
| W204 | Yangmiaozhong | T440 | Wujiang, Jiangsu | 0.000 | 0.999 | 0.000 | 0.000 | 0.000 | 0.000 | 0.000 | 2 |
| W205 | Maoguangdao | T247 | Wuxian, Jiangsu | 0.000 | 0.999 | 0.000 | 0.000 | 0.000 | 0.000 | 0.000 | 2 |
| W206 | Dazhongdao | T266 | Wujiang, Jiangsu | 0.000 | 0.999 | 0.000 | 0.000 | 0.000 | 0.000 | 0.000 | 2 |
| W207 | Sanxiadao | T232 | Wuxi, Jiangsu | 0.000 | 0.999 | 0.000 | 0.000 | 0.000 | 0.000 | 0.000 | 2 |
| W208 | Xiaoqingmang | T604 | Changshu, Jiangsu | 0.000 | 0.999 | 0.000 | 0.000 | 0.000 | 0.000 | 0.000 | 2 |
| W209 | Hongganlizhihong | T123 | Wujiang, Jiangsu | 0.000 | 0.999 | 0.000 | 0.000 | 0.000 | 0.000 | 0.000 | 2 |
| W210 | Wuxidao | T256 | Changshu, Jiangsu | 0.000 | 0.999 | 0.000 | 0.000 | 0.000 | 0.000 | 0.000 | 2 |
| W211 | Wanzhognqiu | T601 | Wuxian, Jiangsu | 0.000 | 0.999 | 0.000 | 0.000 | 0.000 | 0.000 | 0.000 | 2 |
| W212 | Fengjingdao | T273 | Wuxian, Jiangsu | 0.000 | 0.999 | 0.000 | 0.000 | 0.000 | 0.000 | 0.000 | 2 |
| W213 | Liuzhong | T369 | Changshu, Jiangsu | 0.000 | 1.000 | 0.000 | 0.000 | 0.000 | 0.000 | 0.000 | 2 |
| W214 | Cuganlizhihong | T122 | Wuxian, Jiangsu | 0.000 | 0.999 | 0.000 | 0.000 | 0.000 | 0.000 | 0.000 | 2 |
| W215 | Chiguwandao | T147 | Wujiang, Jiangsu | 0.000 | 0.911 | 0.000 | 0.000 | 0.088 | 0.000 | 0.000 | 2 |
| W216 | Jiaobaiyeqing | T76 | Songjiang, Shanghai | 0.000 | 0.999 | 0.000 | 0.000 | 0.000 | 0.000 | 0.000 | 2 |
| W217 | Chiguhong | T148 | Wujiang, Jiangsu | 0.000 | 0.999 | 0.000 | 0.000 | 0.000 | 0.000 | 0.000 | 2 |
| W218 | Fanluoqing | T51 | Kunshan, Jiangsu | 0.000 | 0.999 | 0.000 | 0.000 | 0.000 | 0.000 | 0.000 | 2 |
| W219 | Zaoyedao | T322 | Kunshan, Jiangsu | 0.000 | 0.999 | 0.000 | 0.000 | 0.000 | 0.000 | 0.000 | 2 |
| W220 | Baidiegu | T361 | Wujiang, Jiangsu | 0.000 | 1.000 | 0.000 | 0.000 | 0.000 | 0.000 | 0.000 | 2 |
| W221 | Wangjiadao | T258 | Wujiang, Jiangsu | 0.000 | 1.000 | 0.000 | 0.000 | 0.000 | 0.000 | 0.000 | 2 |
| W222 | Jiangyinzhong | T447 | Jiangyin, Jiangsu | 0.000 | 0.999 | 0.000 | 0.000 | 0.000 | 0.000 | 0.000 | 2 |
| W223 | Eyingbaijingdao | T279 | Jiading, Shanghai | 0.000 | 1.000 | 0.000 | 0.000 | 0.000 | 0.000 | 0.000 | 2 |
| W224 | Tiekewanguangtou | T487 | Wujin, Jiangsu | 0.000 | 1.000 | 0.000 | 0.000 | 0.000 | 0.000 | 0.000 | 2 |
| W225 | Tiekedao | T271 | Wujin, Jiangsu | 0.000 | 0.999 | 0.000 | 0.000 | 0.000 | 0.000 | 0.000 | 2 |
| W226 | Dadaosuitou | T462 | Changshu, Jiangsu | 0.000 | 0.999 | 0.000 | 0.000 | 0.000 | 0.000 | 0.000 | 2 |
| W227 | Aibaidao | T265 | Wujiang, Jiangsu | 0.000 | 0.999 | 0.000 | 0.000 | 0.000 | 0.000 | 0.000 | 2 |
| W228 | Xiepihuang | T665 | Taicang, Jiangsu | 0.000 | 0.999 | 0.000 | 0.000 | 0.000 | 0.000 | 0.000 | 2 |
| W229 | Xiaobaidao | T667 | Wuxian, Jiangsu | 0.000 | 0.999 | 0.000 | 0.000 | 0.000 | 0.000 | 0.000 | 2 |
| W230 | Baishidao | T222 | Taicang, Jiangsu | 0.000 | 0.999 | 0.000 | 0.000 | 0.000 | 0.000 | 0.000 | 2 |
| W231 | Manbaidao | T215 | Wujiang, Jiangsu | 0.000 | 0.999 | 0.000 | 0.000 | 0.000 | 0.000 | 0.000 | 2 |
| W232 | Guangtouluhuabai | T540 | Wuxi, Jiangsu | 0.000 | 0.999 | 0.000 | 0.000 | 0.000 | 0.000 | 0.000 | 2 |
| W233 | Hongmangjing | T630 | Kunshan, Jiangsu | 0.000 | 0.999 | 0.000 | 0.000 | 0.000 | 0.000 | 0.000 | 2 |
| W234 | Wumangyedao | T335 | Changshu, Jiangsu | 0.000 | 0.999 | 0.000 | 0.000 | 0.000 | 0.000 | 0.000 | 2 |
| W235 | Luhuabai | T531 | Wuxian, Jiangsu | 0.000 | 0.999 | 0.000 | 0.000 | 0.000 | 0.000 | 0.000 | 2 |
| W236 | Haidongqing | T19 | Kunshan, Jiangsu | 0.000 | 0.999 | 0.000 | 0.000 | 0.000 | 0.000 | 0.000 | 2 |
| W237 | Shenlenuo | Y26 | Kunshan, Jiangsu | 0.000 | 0.992 | 0.000 | 0.000 | 0.007 | 0.000 | 0.000 | 2 |
| W238 | Xiangqing | XiangT302(♀) / Qingsan2377(♂) | Chongming, Shanghai | 0.000 | 0.947 | 0.000 | 0.000 | 0.052 | 0.000 | 0.000 | 2 |
| W239 | Jinghui418 | Wanlun422/Miyang23 | Shenyang, Liaoning | 0.000 | 0.859 | 0.000 | 0.000 | 0.140 | 0.999 | 0.000 | 2 |
| W240 | Malaihong | unknown | Nanjing, Jiangsu | 0.000 | 0.000 | 0.000 | 0.000 | 0.000 | 0.999 | 0.000 | 6 |
| W241 | Jingnuo4921 | Wanpingshen99010256 | Hefei, Anhui | 0.000 | 0.001 | 0.000 | 0.000 | 0.000 | 0.908 | 0.000 | 6 |
| W242 | Huadao6 | Suzhongshenzi354 | Huaian, Jiangsu | 0.000 | 0.000 | 0.000 | 0.000 | 0.092 | 0.908 | 0.000 | 6 |
| W243 | Fuyu3 | unknown | Yuexi, Anhui | 0.000 | 0.000 | 0.000 | 0.000 | 0.000 | 1.000 | 0.000 | 6 |
| W244 | Dongnongjing424 | Heishendao2005002 | Shenyang, Liaoning | 0.000 | 0.000 | 0.000 | 0.000 | 0.000 | 0.999 | 0.000 | 6 |
| W245 | Dongnong9006 | Heishendao2013016 | Shenyang, Liaoning | 0.000 | 0.000 | 0.000 | 0.000 | 0.000 | 0.999 | 0.000 | 6 |
| W246 | R254 | Xiangqing / Kouxianghong | Chongming, Shanghai | 0.000 | 0.000 | 0.000 | 0.000 | 0.000 | 0.999 | 0.000 | 6 |
| W247 | Jiangyinnuo | T867 | Jiangyin, Jiangsu | 0.000 | 0.000 | 0.000 | 0.000 | 0.000 | 0.999 | 0.000 | 6 |
| W248 | Jinggunuo | T853 | Wuxi, Jiangsu | 0.000 | 0.000 | 0.000 | 0.000 | 0.000 | 0.999 | 0.000 | 6 |
| W249 | Shanhonggu | T103 | Wujiang, Jiangsu | 0.000 | 0.000 | 0.000 | 0.000 | 0.000 | 0.999 | 0.000 | 6 |
| W250 | Wanshengmaohuang | T169 | Wuxi, Jiangsu | 0.000 | 0.000 | 0.000 | 0.000 | 0.000 | 0.999 | 0.000 | 6 |
| W251 | Wanyangdao | T345 | Wuxian, Jiangsu | 0.000 | 0.000 | 0.000 | 0.000 | 0.000 | 0.999 | 0.000 | 6 |
| W252 | Aidazhong | T407 | Wujiang, Jiangsu | 0.000 | 0.000 | 0.000 | 0.000 | 0.000 | 0.999 | 0.000 | 6 |
| W253 | Jijiaohong | T141 | Wuxian, Jiangsu | 0.000 | 0.000 | 0.000 | 0.000 | 0.000 | 1.000 | 0.000 | 6 |
| W254 | Toulaizhong | T426 | Wujiang, Jiangsu | 0.000 | 0.000 | 0.000 | 0.000 | 0.000 | 1.000 | 0.000 | 6 |
| W255 | Huakenuo | T815 | Wujiang, Jiangsu | 0.000 | 0.000 | 0.000 | 0.000 | 0.000 | 1.000 | 0.000 | 6 |
| W256 | Toudengyishixing | T714 | Kunshan, Jiangsu | 0.000 | 0.000 | 0.000 | 0.000 | 0.000 | 1.000 | 0.000 | 6 |
| W257 | Maozitou | T897 | Wujiang, Jiangsu | 0.000 | 0.000 | 0.000 | 0.000 | 0.000 | 1.000 | 0.000 | 6 |
| W258 | Zaonuodao | T868 | Wujiang, Jiangsu | 0.000 | 0.000 | 0.000 | 0.000 | 0.000 | 1.000 | 0.000 | 6 |
| W259 | Datougui | T902 | Changshu, Jiangsu | 0.000 | 0.000 | 0.000 | 0.000 | 0.000 | 1.000 | 0.000 | 6 |
| W260 | Zaoxiaobaidao | T709 | Wuxi, Jiangsu | 0.000 | 0.000 | 0.000 | 0.000 | 0.000 | 1.000 | 0.000 | 6 |
| W261 | Kangzhounuo | T769 | Wujiang, Jiangsu | 0.000 | 0.000 | 0.000 | 0.000 | 0.000 | 1.000 | 0.000 | 6 |
| W262 | Kuobanzhong | T383 | Qingpu, Shanghai | 0.000 | 0.000 | 0.000 | 0.000 | 0.000 | 1.000 | 0.000 | 6 |
| W263 | Yangzhongdao | T342 | Wujiang, Jiangsu | 0.000 | 0.000 | 0.000 | 0.000 | 0.000 | 1.000 | 0.000 | 6 |
| W264 | Huangkewanguangtou | T486 | Wujin, Jiangsu | 0.000 | 0.000 | 0.000 | 0.000 | 0.000 | 1.000 | 0.000 | 6 |
| W265 | Tiehanyishixing | T569 | Wuxi, Jiangsu | 0.000 | 0.000 | 0.000 | 0.000 | 0.000 | 1.000 | 0.000 | 6 |
| W266 | Aijiaoluganhuang | T190 | Changshu, Jiangsu | 0.000 | 0.000 | 0.000 | 0.000 | 0.000 | 1.000 | 0.000 | 6 |
| W267 | Zhonghua3 | Y235 | Haidian, Beijing | 0.000 | 0.000 | 0.000 | 0.000 | 0.000 | 1.000 | 0.000 | 6 |
| W268 | Buxienuo | Y256 | kunshan, Jiangsu | 0.000 | 0.000 | 0.000 | 0.000 | 0.000 | 1.000 | 0.000 | 6 |
| W269 | Wandao68 | Wanpinshen03010384 | Hefei, Anhui | 0.000 | 0.000 | 0.000 | 0.000 | 0.000 | 1.000 | 0.000 | 6 |
| W270 | C418 | GS2004046 | Shenyang, Liaoning | 0.000 | 0.000 | 0.000 | 0.000 | 0.000 | 1.000 | 0.000 | 6 |
| W271 | Fuxiang1 | GS2009029 | Yuexi, Anhui | 0.000 | 0.000 | 0.000 | 0.000 | 0.201 | 0.799 | 0.000 | 6 |
| W272 | Yuedao1 | YD001 | Vietnam | 0.000 | 0.000 | 0.000 | 0.000 | 0.261 | 0.739 | 0.000 | 6 |
| W273 | Yuedao2 | YD002 | Vietnam | 0.000 | 0.000 | 0.000 | 0.000 | 0.250 | 0.749 | 0.000 | 6 |
| W274 | Yuedao3 | YD003 | Vietnam | 0.000 | 0.000 | 0.000 | 0.000 | 0.261 | 0.738 | 0.000 | 6 |
| W275 | Yuedao4 | YD004 | Vietnam | 0.000 | 0.000 | 0.000 | 0.000 | 0.301 | 0.699 | 0.000 | 6 |
| W276 | Yuedao5 | YD005 | Vietnam | 0.000 | 0.000 | 0.000 | 0.000 | 0.282 | 0.717 | 0.000 | 6 |
| W277 | Yuedao6 | YD006 | Vietnam | 0.000 | 0.000 | 0.000 | 0.000 | 0.300 | 0.699 | 0.000 | 6 |
| W278 | Yuedao7 | YD007 | Vietnam | 0.000 | 0.000 | 0.000 | 0.000 | 0.306 | 0.694 | 0.000 | 6 |
| W279 | Yuedao8 | YD008 | Vietnam | 0.000 | 0.000 | 0.000 | 0.000 | 0.305 | 0.695 | 0.000 | 6 |
| W280 | Yuedao9 | YD009 | Vietnam | 0.000 | 0.000 | 0.000 | 0.000 | 0.297 | 0.702 | 0.000 | 6 |
| W281 | Yuedao10 | YD010 | Vietnam | 0.000 | 0.000 | 0.000 | 0.000 | 0.304 | 0.695 | 0.000 | 6 |
| W282 | Yuedao11 | YD011 | Vietnam | 0.000 | 0.000 | 0.000 | 0.000 | 0.313 | 0.687 | 0.000 | 6 |
| W283 | Yuedao12 | YD012 | Vietnam | 0.000 | 0.000 | 0.000 | 0.000 | 0.283 | 0.716 | 0.000 | 6 |
| W284 | Yuedao13 | YD013 | Vietnam | 0.000 | 0.000 | 0.000 | 0.000 | 0.300 | 0.700 | 0.000 | 6 |
| W285 | Yuedao14 | YD014 | Vietnam | 0.000 | 0.000 | 0.000 | 0.000 | 0.207 | 0.729 | 0.000 | 6 |
| W286 | Yuedao15 | YD015 | Vietnam | 0.000 | 0.000 | 0.000 | 0.000 | 0.286 | 0.714 | 0.000 | 6 |
| W287 | Yuedao16 | YD016 | Vietnam | 0.000 | 0.000 | 0.000 | 0.000 | 0.288 | 0.712 | 0.000 | 6 |
| W288 | Yuedao17 | YD017 | Vietnam | 0.000 | 0.000 | 0.000 | 0.000 | 0.999 | 0.000 | 0.000 | 5 |
| W289 | Yuedao18 | YD018 | Vietnam | 0.000 | 0.000 | 0.000 | 0.000 | 0.999 | 0.000 | 0.000 | 5 |
| W290 | Yuedao19 | Y1A02320 | Vietnam | 0.000 | 0.000 | 0.000 | 0.000 | 0.999 | 0.000 | 0.000 | 5 |
| W291 | Yuedao20 | Y1A02387 | Vietnam | 0.000 | 0.000 | 0.000 | 0.000 | 0.999 | 0.000 | 0.000 | 5 |
| W292 | Yuedao21 | YD021 | Vietnam | 0.000 | 0.000 | 0.000 | 0.000 | 0.999 | 0.000 | 0.000 | 5 |
| W293 | Yuedao22 | YD022 | Vietnam | 0.000 | 0.000 | 0.000 | 0.000 | 0.999 | 0.000 | 0.000 | 5 |
| W294 | Yuedao23 | Y1A02321 | Vietnam | 0.000 | 0.000 | 0.000 | 0.000 | 0.999 | 0.000 | 0.000 | 5 |
| W295 | Yuedao24 | Y1A02322 | Vietnam | 0.000 | 0.000 | 0.000 | 0.000 | 0.999 | 0.000 | 0.000 | 5 |
| W296 | Yuedao25 | YD025 | Vietnam | 0.000 | 0.000 | 0.000 | 0.000 | 0.999 | 0.000 | 0.000 | 5 |
| W297 | Yuedao26 | YD026 | Vietnam | 0.000 | 0.000 | 0.000 | 0.000 | 1.000 | 0.000 | 0.000 | 5 |
| W298 | Yuedao27 | Y1A02323 | Vietnam | 0.000 | 0.000 | 0.000 | 0.000 | 1.000 | 0.000 | 0.000 | 5 |
| W299 | Yuedao28 | Y1A02324 | Vietnam | 0.000 | 0.000 | 0.000 | 0.000 | 1.000 | 0.000 | 0.000 | 5 |
| W300 | Yuedao29 | YD029 | Vietnam | 0.000 | 0.000 | 0.000 | 0.000 | 1.000 | 0.000 | 0.000 | 5 |
| W301 | Yuedao30 | YD030 | Vietnam | 0.000 | 0.000 | 0.000 | 0.000 | 1.000 | 0.000 | 0.000 | 5 |
| W302 | Yuedao31 | Y1A02325 | Vietnam | 0.000 | 0.000 | 0.000 | 0.000 | 1.000 | 0.000 | 0.000 | 5 |
| W303 | Yuedao32 | Y1A02326 | Vietnam | 0.000 | 0.000 | 0.000 | 0.000 | 1.000 | 0.000 | 0.000 | 5 |
| W304 | Yuedao33 | YD033 | Vietnam | 0.000 | 0.000 | 0.000 | 0.000 | 1.000 | 0.000 | 0.000 | 5 |
| W305 | Yuedao34 | Y1A02327 | Vietnam | 0.000 | 0.000 | 0.000 | 0.000 | 0.999 | 0.000 | 0.000 | 5 |
| W306 | Yuedao35 | YD035 | Vietnam | 0.000 | 0.000 | 0.000 | 0.000 | 1.000 | 0.000 | 0.000 | 5 |
| W307 | Yuedao36 | YD36 | Vietnam | 0.000 | 0.000 | 0.000 | 0.000 | 1.000 | 0.000 | 0.000 | 5 |
| W308 | Yuedao37 | YD37 | Vietnam | 0.000 | 0.000 | 0.000 | 0.000 | 1.000 | 0.000 | 0.000 | 5 |
| W309 | Yuedao38 | YD38 | Vietnam | 0.000 | 0.000 | 0.000 | 0.000 | 1.000 | 0.000 | 0.000 | 5 |
| W310 | Yuedao39 | YD39 | Vietnam | 0.000 | 0.000 | 0.000 | 0.000 | 1.000 | 0.000 | 0.000 | 5 |
| W311 | Yuedao40 | YD40 | Vietnam | 0.000 | 0.000 | 0.000 | 0.000 | 1.000 | 0.000 | 0.000 | 5 |
| W312 | Yuedao41 | Y1A02328 | Vietnam | 0.000 | 0.000 | 0.000 | 0.000 | 1.000 | 0.000 | 0.000 | 5 |
| W313 | Yuedao42 | YD42 | Vietnam | 0.000 | 0.000 | 0.000 | 0.000 | 1.000 | 0.000 | 0.000 | 5 |
| W314 | Yuedao43 | YD43 | Vietnam | 0.000 | 0.000 | 0.000 | 0.000 | 1.000 | 0.000 | 0.000 | 5 |
| W315 | Yuedao44 | YD44 | Vietnam | 0.000 | 0.000 | 0.000 | 0.000 | 1.000 | 0.000 | 0.000 | 5 |
| W316 | Yuedao45 | Y1A02329 | Vietnam | 0.000 | 0.000 | 0.000 | 0.000 | 0.999 | 0.000 | 0.000 | 5 |
| W317 | Yuedao46 | Y1A02330 | Vietnam | 0.000 | 0.000 | 0.000 | 0.000 | 0.999 | 0.000 | 0.000 | 5 |
| W318 | Yuedao47 | YD47 | Vietnam | 0.000 | 0.000 | 0.000 | 0.000 | 0.999 | 0.000 | 0.000 | 5 |
| W319 | Yuedao48 | YD48 | Vietnam | 0.000 | 0.000 | 0.000 | 0.000 | 0.999 | 0.000 | 0.000 | 5 |
| W320 | Yuedao49 | YD49 | Vietnam | 0.000 | 0.000 | 0.000 | 0.000 | 1.000 | 0.000 | 0.000 | 5 |
| W321 | Yuedao50 | Y1A02331 | Vietnam | 0.000 | 0.000 | 0.000 | 0.000 | 1.000 | 0.000 | 0.000 | 5 |
| W322 | Yuedao51 | Y1A02332 | Vietnam | 0.000 | 0.000 | 0.000 | 0.000 | 0.999 | 0.000 | 0.000 | 5 |
| W323 | Yuedao52 | YD52 | Vietnam | 0.000 | 0.000 | 0.000 | 0.000 | 1.000 | 0.000 | 0.000 | 5 |
| W324 | Yuedao53 | YD53 | Vietnam | 0.000 | 0.000 | 0.000 | 0.000 | 0.619 | 0.380 | 0.000 | 5 |
| W325 | Yuedao54 | Y1A02335 | Vietnam | 0.001 | 0.002 | 0.000 | 0.000 | 0.995 | 0.002 | 0.000 | 5 |
| W326 | Yuedao55 | YD55 | Vietnam | 0.000 | 0.000 | 0.000 | 0.000 | 0.999 | 0.000 | 0.000 | 5 |
| W327 | Yuedao56 | YD56 | Vietnam | 0.000 | 0.000 | 0.000 | 0.000 | 0.999 | 0.000 | 0.000 | 5 |
| W328 | Yuedao57 | Y1A02336 | Vietnam | 0.000 | 0.000 | 0.000 | 0.000 | 0.999 | 0.000 | 0.000 | 5 |
| W329 | Yuedao58 | Y1A02337 | Vietnam | 0.000 | 0.000 | 0.000 | 0.000 | 1.000 | 0.000 | 0.000 | 5 |
| W330 | Yuedao59 | YD59 | Vietnam | 0.000 | 0.000 | 0.000 | 0.000 | 1.000 | 0.000 | 0.000 | 5 |
| W331 | Yuedao60 | YD60 | Vietnam | 0.000 | 0.000 | 0.000 | 0.000 | 0.999 | 0.000 | 0.000 | 5 |
| W332 | Yuedao61 | YD61 | Vietnam | 0.000 | 0.000 | 0.000 | 0.000 | 0.999 | 0.000 | 0.000 | 5 |
| W333 | Yuedao62 | YD62 | Vietnam | 0.000 | 0.000 | 0.000 | 0.000 | 0.999 | 0.000 | 0.000 | 5 |
| W334 | Yuedao63 | YD63 | Vietnam | 0.001 | 0.018 | 0.000 | 0.001 | 0.978 | 0.002 | 0.000 | 5 |
| W335 | Yuedao64 | Y1A02338 | Vietnam | 0.000 | 0.000 | 0.000 | 0.000 | 0.999 | 0.000 | 0.000 | 5 |
| W336 | Yuedao65 | YD65 | Vietnam | 0.001 | 0.010 | 0.000 | 0.002 | 0.986 | 0.000 | 0.000 | 5 |
| W337 | Yuedao66 | YD66 | Vietnam | 0.000 | 0.000 | 0.000 | 0.000 | 0.999 | 0.000 | 0.000 | 5 |
| W338 | Yuedao67 | Y1A02339 | Vietnam | 0.000 | 0.000 | 0.000 | 0.000 | 0.999 | 0.000 | 0.000 | 5 |
| W339 | Yuedao68 | YD68 | Vietnam | 0.000 | 0.000 | 0.000 | 0.000 | 0.999 | 0.000 | 0.000 | 5 |
| W340 | Yuedao69 | YD69 | Vietnam | 0.000 | 0.000 | 0.000 | 0.000 | 0.999 | 0.000 | 0.000 | 5 |
| W341 | Yuedao70 | YD70 | Vietnam | 0.000 | 0.000 | 0.000 | 0.000 | 0.999 | 0.000 | 0.000 | 5 |
| W342 | Yuedao71 | Y1A02340 | Vietnam | 0.000 | 0.000 | 0.000 | 0.000 | 0.999 | 0.000 | 0.000 | 5 |
| W343 | Yuedao72 | Y1A02341 | Vietnam | 0.000 | 0.000 | 0.000 | 0.000 | 0.999 | 0.000 | 0.000 | 5 |
| W344 | Yuedao73 | YD73 | Vietnam | 0.000 | 0.000 | 0.000 | 0.000 | 0.999 | 0.000 | 0.000 | 5 |
| W345 | Yuedao74 | YD74 | Vietnam | 0.000 | 0.000 | 0.000 | 0.000 | 0.999 | 0.000 | 0.000 | 5 |
| W346 | Yuedao75 | Y1A02342 | Vietnam | 0.000 | 0.000 | 0.000 | 0.000 | 1.000 | 0.000 | 0.000 | 5 |
| W347 | Yuedao76 | YD76 | Vietnam | 0.000 | 0.000 | 0.000 | 0.000 | 0.999 | 0.000 | 0.000 | 5 |
| W348 | Yuedao77 | Y1A02389 | Vietnam | 0.000 | 0.008 | 0.000 | 0.002 | 0.989 | 0.000 | 0.000 | 5 |
| W349 | Yuedao78 | Y1A02343 | Vietnam | 0.000 | 0.000 | 0.000 | 0.000 | 0.999 | 0.000 | 0.000 | 5 |
| W350 | Yuedao79 | YD79 | Vietnam | 0.000 | 0.000 | 0.000 | 0.000 | 0.999 | 0.000 | 0.000 | 5 |
| W351 | Yuedao80 | Y1A02390 | Vietnam | 0.000 | 0.000 | 0.000 | 0.000 | 1.000 | 0.000 | 0.000 | 5 |
| W352 | Yuedao81 | Y1A02344 | Vietnam | 0.000 | 0.000 | 0.000 | 0.000 | 0.999 | 0.000 | 0.000 | 5 |
| W353 | Yuedao82 | YD82 | Vietnam | 0.000 | 0.000 | 0.000 | 0.000 | 0.999 | 0.000 | 0.000 | 5 |
| W354 | Yuedao83 | YD83 | Vietnam | 0.000 | 0.000 | 0.000 | 0.000 | 0.619 | 0.000 | 0.000 | 5 |
| W355 | Yuedao84 | Y1A02391 | Vietnam | 0.000 | 0.000 | 0.000 | 0.000 | 0.999 | 0.000 | 0.000 | 5 |
| W356 | Yuedao85 | Y1A02345 | Vietnam | 0.002 | 0.017 | 0.000 | 0.001 | 0.978 | 0.000 | 0.000 | 5 |
| W357 | Yuedao86 | YD86 | Vietnam | 0.000 | 0.000 | 0.000 | 0.000 | 0.999 | 0.000 | 0.000 | 5 |
| W358 | Yuedao87 | YD87 | Vietnam | 0.000 | 0.000 | 0.000 | 0.000 | 1.000 | 0.000 | 0.000 | 5 |
| W359 | Yuedao88 | Y1A02346 | Vietnam | 0.000 | 0.000 | 0.000 | 0.000 | 0.999 | 0.000 | 0.000 | 5 |
| W360 | Yuedao89 | Y1A02347 | Vietnam | 0.000 | 0.000 | 0.000 | 0.000 | 0.999 | 0.000 | 0.000 | 5 |
| W361 | Yuedao90 | Y1A02392 | Vietnam | 0.000 | 0.000 | 0.000 | 0.000 | 0.999 | 0.000 | 0.000 | 5 |
| W362 | Yuedao91 | Y1A02393 | Vietnam | 0.000 | 0.000 | 0.000 | 0.000 | 1.000 | 0.000 | 0.000 | 5 |
| W363 | Yuedao92 | YD92 | Vietnam | 0.000 | 0.000 | 0.000 | 0.000 | 0.999 | 0.000 | 0.000 | 5 |
| W364 | Yuedao93 | Y1A02394 | Vietnam | 0.000 | 0.000 | 0.000 | 0.000 | 1.000 | 0.000 | 0.000 | 5 |
| W365 | Yuedao94 | YD94 | Vietnam | 0.000 | 0.000 | 0.000 | 0.000 | 0.999 | 0.000 | 0.000 | 5 |
| W366 | Yuedao95 | YD95 | Vietnam | 0.000 | 0.017 | 0.000 | 0.000 | 0.981 | 0.001 | 0.000 | 5 |
| W367 | Yuedao96 | YD96 | Vietnam | 0.000 | 0.000 | 0.000 | 0.000 | 1.000 | 0.000 | 0.000 | 5 |
| W368 | Yuedao97 | YD97 | Vietnam | 0.000 | 0.000 | 0.000 | 0.000 | 1.000 | 0.000 | 0.000 | 5 |
| W369 | Yuedao98 | Y1A02348 | Vietnam | 0.000 | 0.000 | 0.000 | 0.000 | 1.000 | 0.000 | 0.000 | 5 |
| W370 | Yuedao99 | Y1A02349 | Vietnam | 0.000 | 0.000 | 0.000 | 0.000 | 1.000 | 0.000 | 0.000 | 5 |
| W371 | Yuedao100 | Y1A02350 | Vietnam | 0.000 | 0.000 | 0.000 | 0.000 | 1.000 | 0.000 | 0.000 | 5 |
| W372 | Yuedao101 | Y1A02351 | Vietnam | 0.000 | 0.000 | 0.000 | 0.000 | 0.999 | 0.000 | 0.000 | 5 |
| W373 | Yuedao102 | Y1A02352 | Vietnam | 0.000 | 0.000 | 0.000 | 0.000 | 0.999 | 0.000 | 0.000 | 5 |
| W374 | Yuedao103 | YD103 | Vietnam | 0.000 | 0.000 | 0.000 | 0.000 | 1.000 | 0.000 | 0.000 | 5 |
| W375 | Yuedao104 | Y1A02353 | Vietnam | 0.000 | 0.000 | 0.000 | 0.000 | 1.000 | 0.000 | 0.000 | 5 |
| W376 | Yuedao105 | Y1A02396 | Vietnam | 0.000 | 0.000 | 0.000 | 0.000 | 0.999 | 0.000 | 0.000 | 5 |
| W377 | Yuedao106 | Y1A02354 | Vietnam | 0.000 | 0.000 | 0.000 | 0.000 | 0.999 | 0.000 | 0.000 | 5 |
| W378 | Yuedao107 | YD107 | Vietnam | 0.001 | 0.007 | 0.007 | 0.001 | 0.983 | 0.001 | 0.000 | 5 |
| W379 | Yuedao108 | Y1A02355 | Vietnam | 0.018 | 0.037 | 0.000 | 0.003 | 0.940 | 0.001 | 0.000 | 5 |
| W380 | Yuedao109 | Y1A02356 | Vietnam | 0.000 | 0.000 | 0.000 | 0.000 | 0.999 | 0.000 | 0.000 | 5 |
| W381 | Yuedao110 | YD110 | Vietnam | 0.000 | 0.000 | 0.000 | 0.000 | 1.000 | 0.000 | 0.000 | 5 |
| W382 | Yuedao111 | Y1A02398 | Vietnam | 0.000 | 0.000 | 0.000 | 0.000 | 0.999 | 0.000 | 0.000 | 5 |
| W383 | Yuedao112 | Y1A02357 | Vietnam | 0.000 | 0.000 | 0.000 | 0.000 | 0.999 | 0.000 | 0.000 | 5 |
| W384 | Yuedao113 | YD113 | Vietnam | 0.001 | 0.544 | 0.000 | 0.000 | 0.453 | 0.002 | 0.000 | 2 |
| W385 | Yuedao114 | Y1A02399 | Vietnam | 0.000 | 0.627 | 0.000 | 0.000 | 0.369 | 0.003 | 0.000 | 2 |
| W386 | Yuedao115 | Y1A02358 | Vietnam | 0.000 | 0.604 | 0.000 | 0.000 | 0.396 | 0.000 | 0.000 | 2 |
| W387 | Yuedao116 | YD116 | Vietnam | 0.000 | 0.625 | 0.000 | 0.000 | 0.374 | 0.000 | 0.000 | 2 |
| W388 | Yuedao117 | YD117 | Vietnam | 0.000 | 0.663 | 0.000 | 0.000 | 0.336 | 0.000 | 0.000 | 2 |
| W389 | Yuedao118 | YD118 | Vietnam | 0.002 | 0.763 | 0.000 | 0.001 | 0.233 | 0.000 | 0.000 | 2 |
| W390 | Yuedao119 | Y1A02359 | Vietnam | 0.000 | 0.676 | 0.000 | 0.000 | 0.323 | 0.000 | 0.000 | 2 |
| W391 | Yuedao120 | Y1A02360 | Vietnam | 0.000 | 0.699 | 0.000 | 0.000 | 0.300 | 0.000 | 0.000 | 2 |
| W392 | Yuedao121 | Y1A02361 | Vietnam | 0.001 | 0.998 | 0.000 | 0.000 | 0.000 | 0.000 | 0.000 | 2 |
| W393 | Jia45 | Jia25/D293//Jia25 | Jiaxing, Zhejiang | 0.000 | 0.999 | 0.000 | 0.000 | 0.000 | 0.000 | 0.000 | 2 |
| W394 | Nannongjing3786 | Guoshendao2008029 | Nanjing, Jiangsu | 0.002 | 0.997 | 0.000 | 0.000 | 0.000 | 0.000 | 0.000 | 2 |
| W395 | 02428 | ZD-05542 | Nanjing, Jiangsu | 0.001 | 0.999 | 0.000 | 0.000 | 0.000 | 0.000 | 0.000 | 2 |
| W396 | Nannongjing4004 | Xihai26/fengqianben | Nanjing, Jiangsu | 0.000 | 0.999 | 0.000 | 0.000 | 0.000 | 0.000 | 0.000 | 2 |
| W397 | Nannongjing4016 | Akei103B/(Aoisora/Kitariku103)F1 | Nanjing, Jiangsu | 0.000 | 0.999 | 0.000 | 0.000 | 0.000 | 0.000 | 0.000 | 2 |
| W398 | Zijianwujing | Wuyujing3/Zidao | Nanjing, Jiangsu | 0.000 | 0.999 | 0.000 | 0.000 | 0.000 | 0.000 | 0.000 | 2 |
| W399 | Ningjing2 | WPS05010476 | Nanjing, Jiangsu | 0.001 | 0.999 | 0.000 | 0.000 | 0.000 | 0.000 | 0.000 | 2 |
| W400 | Wuxiang99-8 | M-02 | Wujin, Jiangsu | 0.000 | 0.999 | 0.000 | 0.000 | 0.000 | 0.000 | 0.000 | 2 |
| W401 | Wuyujing8 | SZS313 | Wujin, Jiangsu | 0.000 | 0.999 | 0.000 | 0.000 | 0.000 | 0.000 | 0.000 | 2 |
| W402 | Nannongjing002 | unknown | Nanjing, Jiangsu | 0.000 | 0.999 | 0.000 | 0.000 | 0.000 | 0.000 | 0.000 | 2 |
| W403 | Nannongjing004 | unknown | Nanjing, Jiangsu | 0.000 | 0.999 | 0.000 | 0.000 | 0.000 | 0.000 | 0.000 | 2 |
| W404 | Huaidao5hao | SZS358 | Huaian, Jiangsu | 0.000 | 0.999 | 0.000 | 0.000 | 0.000 | 0.000 | 0.000 | 2 |
| W405 | Zhongzuo93 | Jinshendao1995001 | Tongzhou, Beijing | 0.000 | 0.999 | 0.000 | 0.000 | 0.000 | 0.000 | 0.000 | 2 |
| W406 | Yandao9 | SS200506 | Yancheng, Jiangsu | 0.000 | 0.999 | 0.000 | 0.000 | 0.000 | 0.000 | 0.000 | 2 |
| W407 | Lianjing4 | SS200704 | Lianyungang, Jiangsu | 0.000 | 0.999 | 0.000 | 0.000 | 0.000 | 0.000 | 0.000 | 2 |
| W408 | Jindao1007 | Guoshendao2004043 | Dongli, Tianjin | 0.000 | 0.999 | 0.000 | 0.000 | 0.000 | 0.000 | 0.000 | 2 |
| W409 | Huajing5 | SS200505 | Huaibei, Jiangsu | 0.000 | 0.999 | 0.000 | 0.000 | 0.000 | 0.000 | 0.000 | 2 |
| W410 | Huajing6 | SS200706 | Huaibei, Jiangsu | 0.000 | 0.999 | 0.000 | 0.000 | 0.000 | 0.000 | 0.000 | 2 |
| W411 | Yangfujing7 | Sushendao200413 | Lixiahe, Jiangsu | 0.000 | 0.999 | 0.000 | 0.000 | 0.000 | 0.000 | 0.000 | 2 |
| W412 | Yangfujing8 | Sushendao200608 | Lixiahe, Jiangsu | 0.000 | 0.999 | 0.000 | 0.000 | 0.000 | 0.000 | 0.000 | 2 |
| W413 | Zhendao99 | SS200106 | Zhenjiang, Jiangsu | 0.000 | 0.999 | 0.000 | 0.000 | 0.000 | 0.000 | 0.000 | 2 |
| W414 | Nanjing42 | Sushendao200616 | Nanjing, Jiangsu | 0.000 | 0.999 | 0.000 | 0.000 | 0.000 | 0.000 | 0.000 | 2 |
| W415 | Lianjing2 | Guoshendao990021 | Lianyungang, Jiangsu | 0.000 | 0.999 | 0.000 | 0.000 | 0.000 | 0.000 | 0.000 | 2 |
| W416 | Huifeng1 | unknown | Yancheng, Jiangsu | 0.000 | 0.999 | 0.000 | 0.000 | 0.000 | 0.000 | 0.000 | 2 |
| W417 | Huifeng2 | unknown | Yancheng, Jiangsu | 0.000 | 0.999 | 0.000 | 0.000 | 0.000 | 0.000 | 0.000 | 2 |
| W418 | Yandao8 | 816922/H88-39 | Yancheng, Jiangsu | 0.000 | 0.999 | 0.000 | 0.000 | 0.000 | 0.000 | 0.000 | 2 |
| W419 | Wuyujing21 | Sushendao200705 | Wujin, Jiangsu | 0.001 | 0.999 | 0.000 | 0.000 | 0.000 | 0.000 | 0.000 | 2 |
| W420 | Shashani | Heishendao2006011 | Haerbin, Heilongjiang | 0.001 | 0.999 | 0.000 | 0.000 | 0.000 | 0.000 | 0.000 | 2 |
| W421 | Muzhan4 | Heishendao2005007 | Mudanjiang, Heilongjiang | 0.001 | 0.999 | 0.000 | 0.000 | 0.000 | 0.000 | 0.000 | 2 |
| W422 | Mudanjiang29 | Heishendao2006007 | Mudanjiang, Heilongjiang | 0.001 | 0.999 | 0.000 | 0.000 | 0.000 | 0.000 | 0.000 | 2 |
| W423 | Mudanjiang28 | Heishendao2006006 | Mudanjiang, Heilongjiang | 0.001 | 0.999 | 0.000 | 0.000 | 0.000 | 0.000 | 0.000 | 2 |
| W424 | Mudanjiang27 | Heishendao2005006 | Mudanjiang, Heilongjiang | 0.001 | 0.999 | 0.000 | 0.000 | 0.000 | 0.000 | 0.000 | 2 |
| W425 | Kenyu20 | Pin1/Jijing13 | Nongken, Heilongjiang | 0.001 | 0.999 | 0.000 | 0.000 | 0.000 | 0.000 | 0.000 | 2 |
| W426 | Heijing8 | Heishendao2007009 | Haerbin, Heilongjiang | 0.002 | 0.998 | 0.000 | 0.000 | 0.000 | 0.000 | 0.000 | 2 |
| W427 | Hejiang21 | Hejiang20/Puxuan10 | Haerbin, Heilongjiang | 0.001 | 0.999 | 0.000 | 0.000 | 0.000 | 0.000 | 0.000 | 2 |
| W428 | Beidao4 | Heishendao2010006 | Haerbin, Heilongjiang | 0.001 | 0.999 | 0.000 | 0.000 | 0.000 | 0.000 | 0.000 | 2 |
| W429 | Beidao3 | Heishendao2000002 | Haerbin, Heilongjiang | 0.002 | 0.998 | 0.000 | 0.000 | 0.000 | 0.000 | 0.000 | 2 |
| W430 | Suijing12 | HS2009013 | Suihua, Heilongjiang | 0.002 | 0.998 | 0.000 | 0.000 | 0.000 | 0.000 | 0.000 | 2 |
| W431 | Songjing12 | HS2008003 | Songhuajiang, Heilongjiang | 0.004 | 0.995 | 0.000 | 0.000 | 0.000 | 0.000 | 0.000 | 2 |
| W432 | Songjing11 | HS2007006 | Songhuajiang, Heilongjiang | 1.000 | 0.000 | 0.000 | 0.000 | 0.000 | 0.000 | 0.000 | 1 |
| W433 | Songjing10 | HS2005005 | Songhuajiang, Heilongjiang | 0.999 | 0.000 | 0.000 | 0.000 | 0.000 | 0.000 | 0.000 | 1 |
| W434 | Dongnong430 | HS2009002 | Haerbin, Heilongjiang | 1.000 | 0.000 | 0.000 | 0.000 | 0.000 | 0.000 | 0.000 | 1 |
| W435 | Dongnong424 | HS2005002 | Haerbin, Heilongjiang | 1.000 | 0.000 | 0.000 | 0.000 | 0.000 | 0.000 | 0.000 | 1 |
| W436 | Longnuo3 | HS2009015 | Haerbin, Heilongjiang | 1.000 | 0.000 | 0.000 | 0.000 | 0.000 | 0.000 | 0.000 | 1 |
| W437 | Longjing28 | HS2009011 | Haerbin, Heilongjiang | 1.000 | 0.000 | 0.000 | 0.000 | 0.000 | 0.000 | 0.000 | 1 |
| W438 | Longjing27 | HS2009010 | Haerbin, Heilongjiang | 1.000 | 0.000 | 0.000 | 0.000 | 0.000 | 0.000 | 0.000 | 1 |
| W439 | Longjing26 | HS2009008 | Haerbin, Heilongjiang | 1.000 | 0.000 | 0.000 | 0.000 | 0.000 | 0.000 | 0.000 | 1 |
| W440 | Longjing25 | HS2009009 | Haerbin, Heilongjiang | 1.000 | 0.000 | 0.000 | 0.000 | 0.000 | 0.000 | 0.000 | 1 |
| W441 | Longjing24 | HS2008017 | Haerbin, Heilongjiang | 1.000 | 0.000 | 0.000 | 0.000 | 0.000 | 0.000 | 0.000 | 1 |
| W442 | Longjing22 | HS2008010 | Haerbin, Heilongjiang | 1.000 | 0.000 | 0.000 | 0.000 | 0.000 | 0.000 | 0.000 | 1 |
| W443 | Longjing21 | HS2008008 | Haerbin, Heilongjiang | 1.000 | 0.000 | 0.000 | 0.000 | 0.000 | 0.000 | 0.000 | 1 |
| W444 | Longjing20 | HS2007004 | Haerbin, Heilongjiang | 1.000 | 0.000 | 0.000 | 0.000 | 0.000 | 0.000 | 0.000 | 1 |
| W445 | Longjing19 | HS2007003 | Haerbin, Heilongjiang | 0.999 | 0.000 | 0.000 | 0.000 | 0.000 | 0.000 | 0.000 | 1 |
| W446 | Longjing18 | HS2007002 | Haerbin, Heilongjiang | 1.000 | 0.000 | 0.000 | 0.000 | 0.000 | 0.000 | 0.000 | 1 |
| W447 | Longjing17 | HS2007001 | Haerbin, Heilongjiang | 1.000 | 0.000 | 0.000 | 0.000 | 0.000 | 0.000 | 0.000 | 1 |
| W448 | Longjing16 | HS2006002 | Haerbin, Heilongjiang | 1.000 | 0.000 | 0.000 | 0.000 | 0.000 | 0.000 | 0.000 | 1 |
| W449 | Longjing15 | HS2006001 | Haerbin, Heilongjiang | 1.000 | 0.000 | 0.000 | 0.000 | 0.000 | 0.000 | 0.000 | 1 |
| W450 | Zhonglongdao1 | HS2008004 | Haerbin, Heilongjiang | 1.000 | 0.000 | 0.000 | 0.000 | 0.000 | 0.000 | 0.000 | 1 |
| W451 | Longdao8 | Muzhan3/Dongqin241 | Haerbin, Heilongjiang | 1.000 | 0.000 | 0.000 | 0.000 | 0.000 | 0.000 | 0.000 | 1 |
| W452 | Longdao6 | HS2006003 | Haerbin, Heilongjiang | 0.999 | 0.000 | 0.000 | 0.000 | 0.000 | 0.000 | 0.000 | 1 |
| W453 | Longdao5 | HS2006002 | Haerbin, Heilongjiang | 1.000 | 0.000 | 0.000 | 0.000 | 0.000 | 0.000 | 0.000 | 1 |
| W454 | Longdao4 | HS2005003 | Haerbin, Heilongjiang | 0.999 | 0.000 | 0.000 | 0.000 | 0.000 | 0.000 | 0.000 | 1 |
| W455 | Kendao19 | HS2009012 | Haerbin, Heilongjiang | 1.000 | 0.000 | 0.000 | 0.000 | 0.000 | 0.000 | 0.000 | 1 |
| W456 | Kendao18 | HS2008012 | Haerbin, Heilongjiang | 0.999 | 0.000 | 0.000 | 0.000 | 0.000 | 0.000 | 0.000 | 1 |
| W457 | Kendao12 | HS2006009 | Haerbin, Heilongjiang | 1.000 | 0.000 | 0.000 | 0.000 | 0.000 | 0.000 | 0.000 | 1 |
| W458 | Kendao13 | HS2008011 | Haerbin, Heilongjiang | 1.000 | 0.000 | 0.000 | 0.000 | 0.000 | 0.000 | 0.000 | 1 |
| W459 | Kendao20 | HS2009003 | Haerbin, Heilongjiang | 1.000 | 0.000 | 0.000 | 0.000 | 0.000 | 0.000 | 0.000 | 1 |
| W460 | Longdun106 | HS2008016 | Haerbin, Heilongjiang | 0.999 | 0.000 | 0.000 | 0.000 | 0.000 | 0.000 | 0.000 | 1 |
| W461 | Longdun105 | HS2007008 | Haerbin, Heilongjiang | 1.000 | 0.000 | 0.000 | 0.000 | 0.000 | 0.000 | 0.000 | 1 |
| W462 | Sanjiang2 | HS2008018 | Haerbin, Heilongjiang | 0.004 | 0.006 | 0.000 | 0.000 | 0.001 | 0.000 | 0.989 | 7 |
| W463 | Nongxiang20 | Xiangshendao2010038 | Changsha, Hunan | 0.000 | 0.000 | 0.000 | 0.000 | 0.000 | 0.000 | 0.999 | 7 |
| W464 | Nongxiangyou205 | Xiangshendao2010029 | Changsha, Hunan | 0.000 | 0.000 | 0.000 | 0.000 | 0.000 | 0.000 | 0.999 | 7 |
| W465 | Nongxiangyou206 | NongxiangA/R206 | Changsha, Hunan | 0.000 | 0.000 | 0.000 | 0.000 | 0.000 | 0.000 | 0.999 | 7 |
| W466 | Yuzhenxiang | XS2008038 | Changsha, Hunan | 0.000 | 0.000 | 0.000 | 0.000 | 0.000 | 0.000 | 0.999 | 7 |
| W467 | Xiangwanxian17 | XS2008035 | Changsha, Hunan | 0.000 | 0.000 | 0.000 | 0.000 | 0.001 | 0.000 | 0.998 | 7 |
| W468 | Huanghuazhan | ES2007017 | Changsha, Hunan | 0.000 | 0.000 | 0.000 | 0.000 | 0.000 | 0.000 | 0.999 | 7 |
| W469 | Nongxiang18 | Xiangshendao2010038 | Changsha, Hunan | 0.000 | 0.000 | 0.000 | 0.000 | 0.000 | 0.000 | 0.999 | 7 |
| W470 | Ribenqing | Yamabiko/Xingfeng | Haerbin, Heilongjiang | 0.000 | 0.000 | 0.000 | 0.000 | 0.000 | 0.000 | 0.999 | 7 |
| W471 | Tijin | NL150 | Haerbin, Heilongjiang | 0.000 | 0.000 | 0.000 | 0.000 | 0.000 | 0.000 | 0.999 | 7 |
| W472 | M1004 | Oochikara Mutant | Haerbin, Heilongjiang | 0.000 | 0.000 | 0.000 | 0.000 | 0.000 | 0.000 | 0.999 | 7 |
| W473 | Zhongguo91 | NL274 | Haerbin, Heilongjiang | 0.000 | 0.000 | 0.000 | 0.000 | 0.000 | 0.000 | 0.999 | 7 |
| W474 | Qiutainxiaoding | Yueguang/Aoyu292 | Haerbin, Heilongjiang | 0.000 | 0.000 | 0.000 | 0.000 | 0.000 | 0.000 | 0.999 | 7 |
| W475 | Cai | Daobei43/Beiming | Haerbin, Heilongjiang | 0.000 | 0.000 | 0.000 | 0.000 | 0.000 | 0.000 | 1.000 | 7 |
| W476 | Qiuguang | Liming/fengjin | Haerbin, Heilongjiang | 0.000 | 0.000 | 0.000 | 0.000 | 0.000 | 0.000 | 1.000 | 7 |
| W477 | Kangbingyueguang | Nonglin22/Nonglin1-R | Haerbin, Heilongjiang | 0.001 | 0.000 | 0.000 | 0.000 | 0.000 | 0.000 | 0.998 | 7 |
| W478 | Youzhiyueguang | Nonglin22/Nonglin1-H | Haerbin, Heilongjiang | 0.000 | 0.000 | 0.000 | 0.000 | 0.000 | 0.000 | 0.999 | 7 |
| W479 | Kasalath | Introduced from Japan | Haerbin, Heilongjiang | 0.000 | 0.000 | 0.000 | 0.000 | 0.000 | 0.000 | 1.000 | 7 |
| W480 | Xiangchuanwuxinbaimi | unknown | Haerbin, Heilongjiang | 0.000 | 0.000 | 0.000 | 0.000 | 0.000 | 0.000 | 1.000 | 7 |
| W481 | Wuyujing24 | Sushendao201209 | Hongze, Jiangsu | 0.000 | 0.000 | 0.000 | 0.000 | 0.000 | 0.000 | 0.999 | 7 |
| W482 | Wuyujing27 | Sushendao201209 | Hongze, Jiangsu | 0.000 | 0.000 | 0.000 | 0.000 | 0.000 | 0.000 | 1.000 | 7 |
| W483 | Heimixiandao-1 | 09-02543 | Nanjing, Jiangsu | 0.000 | 0.000 | 0.000 | 0.000 | 0.000 | 0.000 | 1.000 | 7 |
| W484 | Heimijingdao-2 | 09-02544 | Nanjing, Jiangsu | 0.000 | 0.000 | 0.000 | 0.000 | 0.000 | 0.000 | 0.999 | 7 |
| W485 | Zidao | unknown | Nanjing, Jiangsu | 0.000 | 0.000 | 0.000 | 0.000 | 0.000 | 0.000 | 1.000 | 7 |
| W486 | 9311 | GS2001002 | Yangzhou, Jiangsu | 0.000 | 0.000 | 0.000 | 0.000 | 0.000 | 0.000 | 1.000 | 7 |
| W487 | Zacaodao | unknown | Nanjing, Jiangsu | 0.000 | 0.000 | 0.000 | 0.000 | 0.000 | 0.000 | 1.000 | 7 |
| W488 | Youmang429 | unknown | Nanjing, Jiangsu | 0.000 | 0.000 | 0.000 | 0.000 | 0.000 | 0.000 | 1.000 | 7 |
| W489 | Wanqu429 | unknown | Nanjing, Jiangsu | 0.000 | 0.000 | 0.000 | 0.000 | 0.000 | 0.000 | 1.000 | 7 |
| W490 | Zhili429 | unknown | Nanjing, Jiangsu | 0.000 | 0.000 | 0.000 | 0.000 | 0.000 | 0.000 | 1.000 | 7 |
| W491 | Si4029 | Introduction 4029 | Sihong, Jiangsu | 0.000 | 0.000 | 0.000 | 0.000 | 0.000 | 0.000 | 1.000 | 7 |
| W492 | Si4031 | Introduction4031 | Sihong, Jiangsu | 0.000 | 0.000 | 0.000 | 0.000 | 0.000 | 0.000 | 1.000 | 7 |
| W493 | Si4033 | Introduction4033 | Sihong, Jiangsu | 0.000 | 0.000 | 0.000 | 0.000 | 0.000 | 0.000 | 1.000 | 7 |
| W494 | Si4039 | Introduction4039 | Sihong, Jiangsu | 0.000 | 0.000 | 0.000 | 0.000 | 0.000 | 0.000 | 1.000 | 7 |
| W495 | Si4040 | Introduction4040 | Sihong, Jiangsu | 0.000 | 0.000 | 0.000 | 0.000 | 0.000 | 0.000 | 1.000 | 7 |
| W496 | Si4041 | Introduction4041 | Sihong, Jiangsu | 0.000 | 0.000 | 0.000 | 0.000 | 0.000 | 0.000 | 1.000 | 7 |
| W497 | Si4049 | Introduction4049 | Sihong, Jiangsu | 0.000 | 0.000 | 0.000 | 0.000 | 0.000 | 0.000 | 1.000 | 7 |
| W498 | Si4079 | Introduction4079 | Sihong, Jiangsu | 0.000 | 0.000 | 0.000 | 0.000 | 0.000 | 0.000 | 1.000 | 7 |
| W499 | Si4081 | Introduction4081 | Sihong, Jiangsu | 0.000 | 0.000 | 0.000 | 0.000 | 0.000 | 0.000 | 1.000 | 7 |
| W500 | Si4082 | Introduction4082 | Sihong, Jiangsu | 0.000 | 0.000 | 0.000 | 0.000 | 0.000 | 0.000 | 1.000 | 7 |
| W501 | Si4139 | Introduction4139 | Sihong, Jiangsu | 0.000 | 0.000 | 0.000 | 0.000 | 0.000 | 0.000 | 1.000 | 7 |
| W502 | Si4152 | Introduction4152 | Sihong, Jiangsu | 0.000 | 0.000 | 0.000 | 0.000 | 0.000 | 0.000 | 1.000 | 7 |
| W503 | Si4161 | Introduction4161 | Sihong, Jiangsu | 0.000 | 0.000 | 0.000 | 0.000 | 0.000 | 0.000 | 1.000 | 7 |
| W504 | Si4229 | Introduction4229 | Sihong, Jiangsu | 0.000 | 0.000 | 0.000 | 0.000 | 0.000 | 0.000 | 1.000 | 7 |
| W505 | Si4230 | Introduction4230 | Sihong, Jiangsu | 0.000 | 0.000 | 0.000 | 0.000 | 0.000 | 0.000 | 1.000 | 7 |
| W506 | Si4251 | Introduction4251 | Sihong, Jiangsu | 0.000 | 0.000 | 0.000 | 0.000 | 0.000 | 0.000 | 1.000 | 7 |
| W507 | Si4252 | Introduction4252 | Sihong, Jiangsu | 0.000 | 0.000 | 0.000 | 0.000 | 0.000 | 0.000 | 1.000 | 7 |
| W508 | Si4259 | Introduction4259 | Sihong, Jiangsu | 0.000 | 0.000 | 0.000 | 0.000 | 0.000 | 0.000 | 1.000 | 7 |
| W509 | Si4263 | Introduction4263 | Sihong, Jiangsu | 0.000 | 0.000 | 0.000 | 0.000 | 0.000 | 0.000 | 0.999 | 7 |
| W510 | Si4280 | Introduction4280 | Sihong, Jiangsu | 0.000 | 0.000 | 0.000 | 0.000 | 0.000 | 0.000 | 0.999 | 7 |
| W511 | Si4330 | Introduction4330 | Sihong, Jiangsu | 0.000 | 0.000 | 0.000 | 0.000 | 0.000 | 0.000 | 0.999 | 7 |
| W512 | Si4360 | Introduction4360 | Sihong, Jiangsu | 0.000 | 0.000 | 0.000 | 0.000 | 0.000 | 0.000 | 0.999 | 7 |
| W513 | Si4364 | Introduction4364 | Sihong, Jiangsu | 0.000 | 0.000 | 0.000 | 0.000 | 0.000 | 0.000 | 0.999 | 7 |
| W514 | Digudao | Mutant | Sihong, Jiangsu | 0.000 | 0.000 | 0.000 | 0.000 | 0.000 | 0.000 | 0.999 | 7 |
| W515 | Si4385 | Introduction4385 | Sihong, Jiangsu | 0.000 | 0.000 | 0.000 | 0.000 | 0.000 | 0.000 | 0.999 | 7 |
| W516 | Si4386 | Introduction4386 | Sihong, Jiangsu | 0.000 | 0.000 | 0.000 | 0.000 | 0.000 | 0.000 | 1.000 | 7 |
| W517 | Sihao4141 | Sidao8/zhongdan2 | Sihong, Jiangsu | 0.000 | 0.000 | 0.000 | 0.000 | 0.000 | 0.000 | 0.999 | 7 |
| W518 | Ningjinghui096 | Introduction 096 | Nanjing, Jiangsu | 0.000 | 0.000 | 0.000 | 0.000 | 0.000 | 0.000 | 0.999 | 7 |
| W519 | Ningjinghui117 | Introduction117 | Nanjing, Jiangsu | 0.000 | 0.000 | 0.000 | 0.000 | 0.000 | 0.000 | 0.999 | 7 |
| W520 | Ningjinghui145 | Introduction145 | Nanjing, Jiangsu | 0.000 | 0.000 | 0.000 | 0.000 | 0.000 | 0.000 | 0.999 | 7 |
| W521 | Ningjinghui166 | Introduction166 | Nanjing, Jiangsu | 0.000 | 0.000 | 0.000 | 0.000 | 0.000 | 0.000 | 0.999 | 7 |
| W522 | Ningjinghui208 | Introduction208 | Nanjing, Jiangsu | 0.000 | 0.000 | 0.000 | 0.000 | 0.000 | 0.000 | 0.999 | 7 |
| W523 | Ningjinghui210 | Introduction210 | Nanjing, Jiangsu | 0.000 | 0.000 | 0.000 | 0.000 | 0.000 | 0.000 | 0.999 | 7 |
| W524 | Ningjinghui237 | Introduction237 | Nanjing, Jiangsu | 0.000 | 0.000 | 0.000 | 0.000 | 0.000 | 0.000 | 0.999 | 7 |
| W525 | Ningjinghui246 | Introduction246 | Nanjing, Jiangsu | 0.000 | 0.000 | 0.000 | 0.000 | 0.000 | 0.000 | 0.999 | 7 |
| W526 | Ningjinghui260 | Introduction260 | Nanjing, Jiangsu | 0.000 | 0.000 | 0.000 | 0.000 | 0.000 | 0.000 | 0.999 | 7 |
| W527 | Ningjinghui285 | Introduction285 | Nanjing, Jiangsu | 0.000 | 0.000 | 0.000 | 0.000 | 0.000 | 0.000 | 0.999 | 7 |
| W528 | Ningjinghui286 | Introduction286 | Nanjing, Jiangsu | 0.000 | 0.000 | 0.000 | 0.000 | 0.000 | 0.000 | 0.999 | 7 |
| W529 | Ningjinghui290 | Introduction290 | Nanjing, Jiangsu | 0.000 | 0.000 | 0.000 | 0.000 | 0.000 | 0.000 | 0.999 | 7 |
| W530 | Ningjinghui292 | Introduction292 | Nanjing, Jiangsu | 0.000 | 0.000 | 0.000 | 0.000 | 0.000 | 0.000 | 0.999 | 7 |
| W531 | Ningjinghui293 | Introduction293 | Nanjing, Jiangsu | 0.000 | 0.000 | 0.000 | 0.000 | 0.000 | 0.000 | 1.000 | 7 |
| W532 | Ningjinghui296 | Introduction296 | Nanjing, Jiangsu | 0.000 | 0.000 | 0.000 | 0.000 | 0.000 | 0.000 | 1.000 | 7 |
| W533 | Ningjinghui298 | Introduction298 | Nanjing, Jiangsu | 0.000 | 0.000 | 0.000 | 0.000 | 0.000 | 0.000 | 0.999 | 7 |
| W534 | Ningjinghui338 | Introduction338 | Nanjing, Jiangsu | 0.000 | 0.000 | 0.000 | 0.000 | 0.000 | 0.000 | 0.999 | 7 |
| W535 | Hongyin1009 | Introduction1009 | Nanjing, Jiangsu | 0.000 | 0.000 | 0.000 | 0.000 | 0.000 | 0.000 | 1.000 | 7 |
| W536 | Hongyin1010 | Introduction1010 | Nanjing, Jiangsu | 0.000 | 0.000 | 0.000 | 0.000 | 0.000 | 0.000 | 0.999 | 7 |
| W537 | Hongyin1011 | Introduction1011 | Nanjing, Jiangsu | 0.000 | 0.000 | 0.000 | 0.000 | 0.000 | 0.000 | 0.999 | 7 |
| W538 | Hongyin1012 | Introduction1012 | Nanjing, Jiangsu | 0.000 | 0.000 | 0.000 | 0.000 | 0.000 | 0.000 | 1.000 | 7 |
| W539 | Cai 1 | Yongxi8721 | Haerbin, Heilongjiang | 0.000 | 0.000 | 0.000 | 0.000 | 0.000 | 0.000 | 1.000 | 7 |
| W540 | Nannongjing1R | C57/IR28//Jingyin37 | Nanjing, Jiangsu | 0.000 | 0.000 | 0.000 | 0.000 | 0.000 | 0.000 | 0.999 | 7 |
| W541 | Nannongjing2R | Ke6/Ke3//Jingyin35 | Nanjing, Jiangsu | 0.252 | 0.014 | 0.120 | 0.101 | 0.098 | 0.066 | 0.349 | 7 |
| W542 | Nannongjing3R | Zhanli/Jinghui7623 | Nanjing, Jiangsu | 0.341 | 0.003 | 0.111 | 0.104 | 0.059 | 0.143 | 0.240 | 1 |
| W543 | Ningjing1R-34 | Ningjing1/R254// Ningjing1-CSSL34 | Nanjing, Jiangsu | 0.000 | 0.000 | 1.000 | 0.000 | 0.000 | 0.000 | 0.000 | 3 |
| W544 | Ningjing1R-38 | Ningjing1/R254// Ningjing1-CSSL38 | Nanjing, Jiangsu | 0.000 | 0.000 | 1.000 | 0.000 | 0.000 | 0.000 | 0.000 | 3 |
| W545 | Ningjing1R-39 | Ningjing1/R254// Ningjing1-CSSL39 | Nanjing, Jiangsu | 0.000 | 0.000 | 1.000 | 0.000 | 0.000 | 0.000 | 0.000 | 3 |
| W546 | Ningjing1R-43 | Ningjing1/R254// Ningjing1-CSSL43 | Nanjing, Jiangsu | 0.000 | 0.000 | 1.000 | 0.000 | 0.000 | 0.000 | 0.000 | 3 |
| W547 | Ningjing1R-45 | Ningjing1/R254// Ningjing1-CSSL45 | Nanjing, Jiangsu | 0.000 | 0.000 | 1.000 | 0.000 | 0.000 | 0.000 | 0.000 | 3 |
| W548 | Ningjing1R-97 | Ningjing1/R254// Ningjing1-CSSL97 | Nanjing, Jiangsu | 0.000 | 0.000 | 1.000 | 0.000 | 0.000 | 0.000 | 0.000 | 3 |
| W549 | Ningjing1R-99 | Ningjing1/R254// Ningjing1-CSSL99 | Nanjing, Jiangsu | 0.000 | 0.000 | 1.000 | 0.000 | 0.000 | 0.000 | 0.000 | 3 |
| W550 | Ningjing1R-108 | Ningjing1/R254// Ningjing1-CSSL108 | Nanjing, Jiangsu | 0.000 | 0.000 | 1.000 | 0.000 | 0.000 | 0.000 | 0.000 | 3 |
| W551 | Ningjing1R-122 | Ningjing1/R254// Ningjing1-CSSL122 | Nanjing, Jiangsu | 0.000 | 0.000 | 1.000 | 0.000 | 0.000 | 0.000 | 0.000 | 3 |
| W552 | Ningjing1R-125 | Ningjing1/R254// Ningjing1-CSSL125 | Nanjing, Jiangsu | 0.000 | 0.000 | 1.000 | 0.000 | 0.000 | 0.000 | 0.000 | 3 |
| W553 | Ningjing1R-126 | Ningjing1/R254// Ningjing1-CSSL126 | Nanjing, Jiangsu | 0.000 | 0.000 | 1.000 | 0.000 | 0.000 | 0.000 | 0.000 | 3 |
| W554 | Ningjing1R-128 | Ningjing1/R254// Ningjing1-CSSL128 | Nanjing, Jiangsu | 0.000 | 0.000 | 1.000 | 0.000 | 0.000 | 0.000 | 0.000 | 3 |
| W555 | Ningjing1R-129 | Ningjing1/R254// Ningjing1-CSSL129 | Nanjing, Jiangsu | 0.000 | 0.000 | 1.000 | 0.000 | 0.000 | 0.000 | 0.000 | 3 |
| W556 | Ningjing1R-130 | Ningjing1/R254// Ningjing1-CSSL130 | Nanjing, Jiangsu | 0.000 | 0.000 | 1.000 | 0.000 | 0.000 | 0.000 | 0.000 | 3 |
| W557 | Ningjing1R-132 | Ningjing1/R254// Ningjing1-CSSL132 | Nanjing, Jiangsu | 0.000 | 0.000 | 1.000 | 0.000 | 0.000 | 0.000 | 0.000 | 3 |
| W558 | Ningjing1R-133 | Ningjing1/R254// Ningjing1-CSSL133 | Nanjing, Jiangsu | 0.000 | 0.000 | 1.000 | 0.000 | 0.000 | 0.000 | 0.000 | 3 |
| W559 | Ningjing1R-35 | Ningjing1/R254// Ningjing1-CSSL35 | Nanjing, Jiangsu | 0.000 | 0.000 | 1.000 | 0.000 | 0.000 | 0.000 | 0.000 | 3 |
| W560 | Ningjing1R-36 | Ningjing1/R254// Ningjing1-CSSL36 | Nanjing, Jiangsu | 0.000 | 0.000 | 1.000 | 0.000 | 0.000 | 0.000 | 0.000 | 3 |
| W561 | Ningjing1R-37 | Ningjing1/R254// Ningjing1-CSSL37 | Nanjing, Jiangsu | 0.000 | 0.000 | 1.000 | 0.000 | 0.000 | 0.000 | 0.000 | 3 |
| W562 | Ningjing1R-40 | Ningjing1/R254// Ningjing1-CSSL40 | Nanjing, Jiangsu | 0.000 | 0.000 | 1.000 | 0.000 | 0.000 | 0.000 | 0.000 | 3 |
| W563 | Ningjing1R-41 | Ningjing1/R254// Ningjing1-CSSL41 | Nanjing, Jiangsu | 0.000 | 0.000 | 1.000 | 0.000 | 0.000 | 0.000 | 0.000 | 3 |
| W564 | Ningjing1R-42 | Ningjing1/R254// Ningjing1-CSSL42 | Nanjing, Jiangsu | 0.000 | 0.000 | 1.000 | 0.000 | 0.000 | 0.000 | 0.000 | 3 |
| W565 | Ningjing1R-44 | Ningjing1/R254// Ningjing1-CSSL44 | Nanjing, Jiangsu | 0.000 | 0.000 | 1.000 | 0.000 | 0.000 | 0.000 | 0.000 | 3 |
| W566 | Ningjing1R-47 | Ningjing1/R254// Ningjing1-CSSL47 | Nanjing, Jiangsu | 0.000 | 0.000 | 1.000 | 0.000 | 0.000 | 0.000 | 0.000 | 3 |
| W567 | Ningjing1R-50 | Ningjing1/R254// Ningjing1-CSSL50 | Nanjing, Jiangsu | 0.000 | 0.000 | 1.000 | 0.000 | 0.000 | 0.000 | 0.000 | 3 |
| W568 | Ningjing1R-53 | Ningjing1/R254// Ningjing1-CSSL53 | Nanjing, Jiangsu | 0.000 | 0.000 | 1.000 | 0.000 | 0.000 | 0.000 | 0.000 | 3 |
| W569 | Ningjing1R-56 | Ningjing1/R254// Ningjing1-CSSL56 | Nanjing, Jiangsu | 0.000 | 0.000 | 1.000 | 0.000 | 0.000 | 0.000 | 0.000 | 3 |
| W570 | Ningjing1R-104 | Ningjing1/R254// Ningjing1-CSSL104 | Nanjing, Jiangsu | 0.000 | 0.000 | 1.000 | 0.000 | 0.000 | 0.000 | 0.000 | 3 |
| W571 | Ningjing1R-107 | Ningjing1/R254// Ningjing1-CSSL107 | Nanjing, Jiangsu | 0.000 | 0.000 | 1.000 | 0.000 | 0.000 | 0.000 | 0.000 | 3 |
| W572 | Ningjing1R-111 | Ningjing1/R254// Ningjing1-CSSL111 | Nanjing, Jiangsu | 0.000 | 0.000 | 1.000 | 0.000 | 0.000 | 0.000 | 0.000 | 3 |
| W573 | Ningjing1R-114 | Ningjing1/R254// Ningjing1-CSSL114 | Nanjing, Jiangsu | 0.000 | 0.000 | 1.000 | 0.000 | 0.000 | 0.000 | 0.000 | 3 |
| W574 | Ningjing1R-115 | Ningjing1/R254// Ningjing1-CSSL115 | Nanjing, Jiangsu | 0.000 | 0.000 | 1.000 | 0.000 | 0.000 | 0.000 | 0.000 | 3 |
| W575 | Ningjing1R-117 | Ningjing1/R254// Ningjing1-CSSL117 | Nanjing, Jiangsu | 0.000 | 0.000 | 1.000 | 0.000 | 0.000 | 0.000 | 0.000 | 3 |
| W576 | Ningjing1R-123 | Ningjing1/R254// Ningjing1-CSSL123 | Nanjing, Jiangsu | 0.000 | 0.000 | 1.000 | 0.000 | 0.000 | 0.000 | 0.000 | 3 |
| W577 | Ningjing1R-127 | Ningjing1/R254// Ningjing1-CSSL127 | Nanjing, Jiangsu | 0.000 | 0.000 | 1.000 | 0.000 | 0.000 | 0.000 | 0.000 | 3 |
| W578 | Ningjing1R-131 | Ningjing1/R254// Ningjing1-CSSL131 | Nanjing, Jiangsu | 0.000 | 0.000 | 1.000 | 0.000 | 0.000 | 0.000 | 0.000 | 3 |
| W579 | Ningjing1R-21 | Ningjing1/R254// Ningjing1-CSSL21 | Nanjing, Jiangsu | 0.000 | 0.000 | 1.000 | 0.000 | 0.000 | 0.000 | 0.000 | 3 |
| W580 | Ningjing1R-26 | Ningjing1/R254// Ningjing1-CSSL26 | Nanjing, Jiangsu | 0.000 | 0.000 | 1.000 | 0.000 | 0.000 | 0.000 | 0.000 | 3 |
| W581 | Ningjing1R-30 | Ningjing1/R254// Ningjing1-CSSL30 | Nanjing, Jiangsu | 0.000 | 0.000 | 1.000 | 0.000 | 0.000 | 0.000 | 0.000 | 3 |
| W582 | Ningjing1R-68 | Ningjing1/R254// Ningjing1-CSSL68 | Nanjing, Jiangsu | 0.000 | 0.000 | 1.000 | 0.000 | 0.000 | 0.000 | 0.000 | 3 |
| W583 | Ningjing1R-70 | Ningjing1/R254// Ningjing1-CSSL70 | Nanjing, Jiangsu | 0.000 | 0.000 | 1.000 | 0.000 | 0.000 | 0.000 | 0.000 | 3 |
| W584 | Ningjing1R-118 | Ningjing1/R254// Ningjing1-CSSL118 | Nanjing, Jiangsu | 0.000 | 0.000 | 1.000 | 0.000 | 0.000 | 0.000 | 0.000 | 3 |
| W585 | Ningjing1R-137 | Ningjing1/R254// Ningjing1-CSSL137 | Nanjing, Jiangsu | 0.000 | 0.000 | 1.000 | 0.000 | 0.000 | 0.000 | 0.000 | 3 |
| W586 | Ningjing1R-143 | Ningjing1/R254// Ningjing1-CSSL143 | Nanjing, Jiangsu | 0.000 | 0.000 | 1.000 | 0.000 | 0.000 | 0.000 | 0.000 | 3 |
| W587 | Ningjing1R-134 | Ningjing1/R254// Ningjing1-CSSL134 | Nanjing, Jiangsu | 0.000 | 0.000 | 1.000 | 0.000 | 0.000 | 0.000 | 0.000 | 3 |
| W588 | Ningjing1R-135 | Ningjing1/R254// Ningjing1-CSSL135 | Nanjing, Jiangsu | 0.000 | 0.000 | 1.000 | 0.000 | 0.000 | 0.000 | 0.000 | 3 |
| W589 | Ningjing1R-136 | Ningjing1/R254// Ningjing1-CSSL136 | Nanjing, Jiangsu | 0.000 | 0.000 | 1.000 | 0.000 | 0.000 | 0.000 | 0.000 | 3 |
| W590 | Ningjing1R-138 | Ningjing1/R254// Ningjing1-CSSL138 | Nanjing, Jiangsu | 0.000 | 0.000 | 1.000 | 0.000 | 0.000 | 0.000 | 0.000 | 3 |
| W591 | Ningjing1R-139 | Ningjing1/R254// Ningjing1-CSSL139 | Nanjing, Jiangsu | 0.000 | 0.000 | 1.000 | 0.000 | 0.000 | 0.000 | 0.000 | 3 |
| W592 | Ningjing1R-140 | Ningjing1/R254// Ningjing1-CSSL140 | Nanjing, Jiangsu | 0.000 | 0.000 | 1.000 | 0.000 | 0.000 | 0.000 | 0.000 | 3 |
| W593 | Ningjing1R-141 | Ningjing1/R254// Ningjing1-CSSL141 | Nanjing, Jiangsu | 0.000 | 0.000 | 1.000 | 0.000 | 0.000 | 0.000 | 0.000 | 3 |
| W594 | Ningjing1R-142 | Ningjing1/R254// Ningjing1-CSSL142 | Nanjing, Jiangsu | 0.000 | 0.000 | 1.000 | 0.000 | 0.000 | 0.000 | 0.000 | 3 |
| W595 | Ningjing1R-144 | Ningjing1/R254// Ningjing1-CSSL144 | Nanjing, Jiangsu | 0.000 | 0.000 | 1.000 | 0.000 | 0.000 | 0.000 | 0.000 | 3 |
| W596 | Ningjing1R-18 | Ningjing1/R254// Ningjing1-CSSL18 | Nanjing, Jiangsu | 0.000 | 0.000 | 1.000 | 0.000 | 0.000 | 0.000 | 0.000 | 3 |
| W597 | Ningjing1R-59 | Ningjing1/R254// Ningjing1-CSSL59 | Nanjing, Jiangsu | 0.000 | 0.000 | 1.000 | 0.000 | 0.000 | 0.000 | 0.000 | 3 |
| W598 | Ningjing1R-60 | Ningjing1/R254// Ningjing1-CSSL60 | Nanjing, Jiangsu | 0.000 | 0.000 | 1.000 | 0.000 | 0.000 | 0.000 | 0.000 | 3 |
| W599 | Ningjing1R-61 | Ningjing1/R254// Ningjing1-CSSL61 | Nanjing, Jiangsu | 0.000 | 0.000 | 1.000 | 0.000 | 0.000 | 0.000 | 0.000 | 3 |
| W600 | Ningjing1R-62 | Ningjing1/R254// Ningjing1-CSSL62 | Nanjing, Jiangsu | 0.000 | 0.000 | 1.000 | 0.000 | 0.000 | 0.000 | 0.000 | 3 |
| W601 | Ningjing1R-63 | Ningjing1/R254// Ningjing1-CSSL63 | Nanjing, Jiangsu | 0.000 | 0.000 | 1.000 | 0.000 | 0.000 | 0.000 | 0.000 | 3 |
| W602 | Ningjing1R-100 | Ningjing1/R254// Ningjing1-CSSL100 | Nanjing, Jiangsu | 0.000 | 0.000 | 1.000 | 0.000 | 0.000 | 0.000 | 0.000 | 3 |
| W603 | Ningjing1R-102 | Ningjing1/R254// Ningjing1-CSSL102 | Nanjing, Jiangsu | 0.000 | 0.000 | 1.000 | 0.000 | 0.000 | 0.000 | 0.000 | 3 |
| W604 | Ningjing1R-124 | Ningjing1/R254// Ningjing1-CSSL124 | Nanjing, Jiangsu | 0.000 | 0.000 | 1.000 | 0.000 | 0.000 | 0.000 | 0.000 | 3 |
| W605 | Ningjing1R-55 | Ningjing1/R254// Ningjing1-CSSL55 | Nanjing, Jiangsu | 0.000 | 0.000 | 1.000 | 0.000 | 0.000 | 0.000 | 0.000 | 3 |
| W606 | Ningjing1R-101 | Ningjing1/R254// Ningjing1-CSSL101 | Nanjing, Jiangsu | 0.000 | 0.000 | 1.000 | 0.000 | 0.000 | 0.000 | 0.000 | 3 |
| W607 | Ningjing1R-109 | Ningjing1/R254// Ningjing1-CSSL109 | Nanjing, Jiangsu | 0.000 | 0.000 | 1.000 | 0.000 | 0.000 | 0.000 | 0.000 | 3 |
| W608 | Ningjing1R-110 | Ningjing1/R254// Ningjing1-CSSL | Nanjing, Jiangsu | 0.000 | 0.000 | 1.000 | 0.000 | 0.000 | 0.000 | 0.000 | 3 |
| W609 | Ningjing1R-112 | Ningjing1/R254// Ningjing1-CSSL112 | Nanjing, Jiangsu | 0.000 | 0.000 | 1.000 | 0.000 | 0.000 | 0.000 | 0.000 | 3 |
| W610 | Ningjing1R-116 | Ningjing1/R254// Ningjing1-CSSL116 | Nanjing, Jiangsu | 0.000 | 0.000 | 1.000 | 0.000 | 0.000 | 0.000 | 0.000 | 3 |
| W611 | Ningjing1R-149 | Ningjing1/R254// Ningjing1-CSSL149 | Nanjing, Jiangsu | 0.000 | 0.000 | 1.000 | 0.000 | 0.000 | 0.000 | 0.000 | 3 |
| W612 | Ningjing1R-13 | Ningjing1/R254// Ningjing1-CSSL13 | Nanjing, Jiangsu | 0.000 | 0.000 | 1.000 | 0.000 | 0.000 | 0.000 | 0.000 | 3 |
| W613 | Ningjing1R-15 | Ningjing1/R254// Ningjing1-CSSL15 | Nanjing, Jiangsu | 0.000 | 0.000 | 1.000 | 0.000 | 0.000 | 0.000 | 0.000 | 3 |
| W614 | Ningjing1R-17 | Ningjing1/R254// Ningjing1-CSSL17 | Nanjing, Jiangsu | 0.000 | 0.000 | 1.000 | 0.000 | 0.000 | 0.000 | 0.000 | 3 |
| W615 | Ningjing1R-22 | Ningjing1/R254// Ningjing1-CSSL22 | Nanjing, Jiangsu | 0.000 | 0.000 | 1.000 | 0.000 | 0.000 | 0.000 | 0.000 | 3 |
| W616 | Ningjing1R-24 | Ningjing1/R254// Ningjing1-CSSL24 | Nanjing, Jiangsu | 0.000 | 0.000 | 1.000 | 0.000 | 0.000 | 0.000 | 0.000 | 3 |
| W617 | Ningjing1R-27 | Ningjing1/R254// Ningjing1-CSSL27 | Nanjing, Jiangsu | 0.000 | 0.000 | 1.000 | 0.000 | 0.000 | 0.000 | 0.000 | 3 |
| W618 | Ningjing1R-28 | Ningjing1/R254// Ningjing1-CSSL28 | Nanjing, Jiangsu | 0.000 | 0.000 | 1.000 | 0.000 | 0.000 | 0.000 | 0.000 | 3 |
| W619 | Ningjing1R-31 | Ningjing1/R254// Ningjing1-CSSL31 | Nanjing, Jiangsu | 0.000 | 0.000 | 1.000 | 0.000 | 0.000 | 0.000 | 0.000 | 3 |
| W620 | Ningjing1R-32 | Ningjing1/R254// Ningjing1-CSSL32 | Nanjing, Jiangsu | 0.000 | 0.000 | 1.000 | 0.000 | 0.000 | 0.000 | 0.000 | 3 |
| W621 | Ningjing1R-33 | Ningjing1/R254// Ningjing1-CSSL33 | Nanjing, Jiangsu | 0.000 | 0.000 | 1.000 | 0.000 | 0.000 | 0.000 | 0.000 | 3 |
| W622 | Ningjing1R-69 | Ningjing1/R254// Ningjing1-CSSL69 | Nanjing, Jiangsu | 0.000 | 0.000 | 1.000 | 0.000 | 0.000 | 0.000 | 0.000 | 3 |
| W623 | Ningjing1R-46 | Ningjing1/R254// Ningjing1-CSSL46 | Nanjing, Jiangsu | 0.000 | 0.000 | 1.000 | 0.000 | 0.000 | 0.000 | 0.000 | 3 |
| W624 | Ningjing1R-48 | Ningjing1/R254// Ningjing1-CSSL48 | Nanjing, Jiangsu | 0.000 | 0.000 | 1.000 | 0.000 | 0.000 | 0.000 | 0.000 | 3 |
| W625 | Ningjing1R-49 | Ningjing1/R254// Ningjing1-CSSL49 | Nanjing, Jiangsu | 0.000 | 0.000 | 1.000 | 0.000 | 0.000 | 0.000 | 0.000 | 3 |
| W626 | Ningjing1R-52 | Ningjing1/R254// Ningjing1-CSSL52 | Nanjing, Jiangsu | 0.000 | 0.000 | 1.000 | 0.000 | 0.000 | 0.000 | 0.000 | 3 |
| W627 | Ningjing1R-54 | Ningjing1/R254// Ningjing1-CSSL54 | Nanjing, Jiangsu | 0.000 | 0.000 | 1.000 | 0.000 | 0.000 | 0.000 | 0.000 | 3 |
| W628 | Ningjing1R-57 | Ningjing1/R254// Ningjing1-CSSL57 | Nanjing, Jiangsu | 0.000 | 0.000 | 1.000 | 0.000 | 0.000 | 0.000 | 0.000 | 3 |
