# Supplementary material for: Genetic Diversity and Elite Allele Mining for Grain Traits in Rice (Oryza sativa L.) by Association Mapping
Source: Front Plant Sci. 2016 Jun 7;7:787. doi: 10.3389/fpls.2016.00787 (PMC4896222; doi:10.3389/fpls.2016.00787)
Supplement: Supplementary file 2 [file Table2.DOC]

Supplementary table S2 Summary statistics for the 262 SSR markers used in this study

| Code | Locus | Chr. | Position(cM) | Allele number | Gene diversity | PIC |  | Code | Locus | Chr. No. | Position(cM) | Allele number | Gene diversity | PIC |
| --- | --- | --- | --- | --- | --- | --- | --- | --- | --- | --- | --- | --- | --- | --- |
| 1 | RM84 | 1 | 18.8 | 7 | 0.4408 | 0.4126 |  | 133 | RM136 | 6 | 53 | 19 | 0.8689 | 0.8571 |
| 2 | RM1-003 | 1 | 19.9 | 12 | 0.8272 | 0.8067 |  | 134 | RM3330 | 6 | 61.6 | 14 | 0.8842 | 0.8742 |
| 3 | RM283 | 1 | 25.4 | 8 | 0.8438 | 0.824 |  | 135 | RM3187 | 6 | 73.2 | 6 | 0.6971 | 0.6436 |
| 4 | RM3453 | 1 | 25.4 | 14 | 0.8628 | 0.8498 |  | 136 | RM7579 | 6 | 84.5 | 6 | 0.7418 | 0.6947 |
| 5 | RM1 | 1 | 29.7 | 11 | 0.7892 | 0.7673 |  | 137 | RM8239 | 6 | 91.9 | 5 | 0.7348 | 0.6901 |
| 6 | RM259 | 1 | 38.8 | 21 | 0.8803 | 0.8698 |  | 138 | RM454 | 6 | 99.3 | 4 | 0.5339 | 0.4778 |
| 7 | RM583 | 1 | 43.2 | 6 | 0.7068 | 0.6533 |  | 139 | RM7309 | 6 | 100.3 | 16 | 0.8555 | 0.8424 |
| 8 | RM490 | 1 | 51 | 5 | 0.3021 | 0.2861 |  | 140 | RM528 | 6 | 100.8 | 11 | 0.7446 | 0.71 |
| 9 | RM8095 | 1 | 60.6 | 9 | 0.8303 | 0.8084 |  | 141 | RM3138 | 6 | 110.6 | 12 | 0.8391 | 0.8205 |
| 10 | RM140 | 1 | 65.4 | 8 | 0.2914 | 0.2803 |  | 142 | RM162 | 6 | 114.9 | 21 | 0.9088 | 0.9021 |
| 11 | RM562 | 1 | 78.4 | 21 | 0.9205 | 0.9148 |  | 143 | RM6811 | 6 | 115.6 | 15 | 0.9014 | 0.8931 |
| 12 | RM9 | 1 | 92.4 | 16 | 0.8185 | 0.7986 |  | 144 | RM345 | 6 | 123.9 | 7 | 0.5979 | 0.5405 |
| 13 | RM129 | 1 | 93 | 5 | 0.3299 | 0.3177 |  | 145 | RM5753 | 6 | 124.4 | 21 | 0.8749 | 0.866 |
| 14 | RM5 | 1 | 98.5 | 10 | 0.8335 | 0.8118 |  | 146 | RM295 | 7 | 0 | 13 | 0.6886 | 0.6606 |
| 15 | RM1231 | 1 | 123.2 | 16 | 0.9001 | 0.8919 |  | 147 | RM125 | 7 | 24.8 | 4 | 0.5625 | 0.4659 |
| 16 | RM128 | 1 | 126.5 | 16 | 0.8451 | 0.8297 |  | 148 | RM180 | 7 | 30.1 | 8 | 0.6294 | 0.5936 |
| 17 | RM297 | 1 | 132 | 10 | 0.7721 | 0.7405 |  | 149 | RM542 | 7 | 34.7 | 7 | 0.8017 | 0.7725 |
| 18 | RM246 | 1 | 134.6 | 16 | 0.8685 | 0.8548 |  | 150 | RM8263 | 7 | 35.7 | 8 | 0.6675 | 0.6132 |
| 19 | RM212 | 1 | 135.8 | 5 | 0.4171 | 0.394 |  | 151 | RM418 | 7 | 42.1 | 9 | 0.7265 | 0.6856 |
| 20 | RM5389 | 1 | 142.4 | 13 | 0.8603 | 0.8447 |  | 152 | RM346 | 7 | 47 | 9 | 0.7778 | 0.7497 |
| 21 | RM486 | 1 | 153.5 | 10 | 0.7199 | 0.6777 |  | 153 | RM2530 | 7 | 53.4 | 15 | 0.8763 | 0.8646 |
| 22 | RM265 | 1 | 155.9 | 8 | 0.7213 | 0.6853 |  | 154 | RM336 | 7 | 61 | 12 | 0.8228 | 0.8038 |
| 23 | RM3482 | 1 | 157.6 | 14 | 0.8431 | 0.8303 |  | 155 | RM5380 | 7 | 67 | 10 | 0.7713 | 0.7354 |
| 24 | RM6831 | 1 | 181.8 | 9 | 0.7542 | 0.7185 |  | 156 | RM6011 | 7 | 73.2 | 14 | 0.896 | 0.887 |
| 25 | RM14 | 1 | 194 | 14 | 0.8774 | 0.866 |  | 157 | RM505 | 7 | 78.6 | 9 | 0.8305 | 0.8093 |
| 26 | RM5340 | 2 | 36.3 | 16 | 0.879 | 0.8677 |  | 158 | RM3589 | 7 | 89.8 | 13 | 0.817 | 0.7952 |
| 27 | RM7288 | 2 | 42.4 | 23 | 0.9029 | 0.8955 |  | 159 | RM11 | 7 | 93.8 | 8 | 0.7658 | 0.7319 |
| 28 | RM5356 | 2 | 43.3 | 10 | 0.8468 | 0.8281 |  | 160 | RM234 | 7 | 93.9 | 14 | 0.818 | 0.8006 |
| 29 | RM1358 | 2 | 48.1 | 10 | 0.8251 | 0.802 |  | 161 | RM134 | 7 | 99.6 | 10 | 0.8363 | 0.8166 |
| 30 | RM1313 | 2 | 51.1 | 10 | 0.8045 | 0.7773 |  | 162 | RM1306 | 7 | 116.1 | 13 | 0.8408 | 0.8268 |
| 31 | RM324 | 2 | 51.1 | 5 | 0.4238 | 0.3837 |  | 163 | RM82 | 7 | 128.9 | 4 | 0.2812 | 0.2663 |
| 32 | RM327 | 2 | 51.9 | 11 | 0.8428 | 0.8236 |  | 164 | RM506 | 8 | 0 | 12 | 0.7941 | 0.7709 |
| 33 | RM301 | 2 | 53.5 | 8 | 0.7848 | 0.7554 |  | 165 | RM1019 | 8 | 0.5 | 18 | 0.875 | 0.8636 |
| 34 | RM300 | 2 | 54.6 | 15 | 0.8832 | 0.8722 |  | 166 | RM152 | 8 | 9.4 | 13 | 0.8129 | 0.79 |
| 35 | RM262 | 2 | 70.2 | 10 | 0.7767 | 0.7451 |  | 167 | RM1235 | 8 | 12.8 | 5 | 0.597 | 0.5261 |
| 36 | RM5427 | 2 | 84.6 | 13 | 0.702 | 0.6589 |  | 168 | RM6863 | 8 | 16.4 | 11 | 0.7887 | 0.7582 |
| 37 | RM3688 | 2 | 88.2 | 8 | 0.8526 | 0.8348 |  | 169 | RM4085 | 8 | 35.7 | 12 | 0.854 | 0.8392 |
| 38 | RM183 | 2 | 93.5 | 15 | 0.8612 | 0.8462 |  | 170 | RM544 | 8 | 38.5 | 15 | 0.9129 | 0.9063 |
| 39 | RM5804 | 2 | 98.2 | 8 | 0.7783 | 0.7455 |  | 171 | RM8243 | 8 | 50.8 | 12 | 0.8788 | 0.8665 |
| 40 | RM106 | 2 | 101.5 | 18 | 0.902 | 0.8942 |  | 172 | RM25 | 8 | 52.2 | 12 | 0.7787 | 0.75 |
| 41 | RM6361 | 2 | 102.9 | 8 | 0.7478 | 0.7131 |  | 173 | RM331 | 8 | 59 | 11 | 0.802 | 0.7743 |
| 42 | RM573 | 2 | 118.1 | 15 | 0.8366 | 0.8188 |  | 174 | RM72 | 8 | 60.9 | 14 | 0.8059 | 0.7873 |
| 43 | RM450 | 2 | 122.8 | 7 | 0.8095 | 0.7849 |  | 175 | RM6215 | 8 | 66.8 | 11 | 0.7922 | 0.77 |
| 44 | RM7598 | 2 | 126.4 | 7 | 0.5802 | 0.5562 |  | 176 | RM7556 | 8 | 86.7 | 12 | 0.8436 | 0.8243 |
| 45 | RM263 | 2 | 127.5 | 14 | 0.8629 | 0.8492 |  | 177 | RM6976 | 8 | 92.2 | 19 | 0.8928 | 0.8842 |
| 46 | RM112 | 2 | 137.5 | 9 | 0.6479 | 0.6206 |  | 178 | RM80 | 8 | 103.7 | 13 | 0.6368 | 0.6189 |
| 47 | RM525 | 2 | 143.7 | 19 | 0.852 | 0.8398 |  | 179 | RM502 | 8 | 109.3 | 15 | 0.8606 | 0.846 |
| 48 | RM213 | 2 | 150.5 | 11 | 0.8416 | 0.823 |  | 180 | RM3754 | 8 | 112.6 | 7 | 0.8141 | 0.7897 |
| 49 | RM208 | 2 | 154.1 | 6 | 0.6547 | 0.597 |  | 181 | RM6948 | 8 | 114.4 | 7 | 0.7235 | 0.6894 |
| 50 | RM3850 | 2 | 156.3 | 14 | 0.8521 | 0.8367 |  | 182 | RM433 | 8 | 116 | 7 | 0.2615 | 0.2538 |
| 51 | RM498 | 2 | 156.3 | 19 | 0.821 | 0.7998 |  | 183 | RM281 | 8 | 128.1 | 14 | 0.8372 | 0.8181 |
| 52 | RM48 | 2 | 191.2 | 10 | 0.4334 | 0.4196 |  | 184 | RM264 | 8 | 138.2 | 12 | 0.8649 | 0.8503 |
| 53 | RM266 | 2 | 192.2 | 11 | 0.7587 | 0.7297 |  | 185 | RM1328 | 9 | 0 | 14 | 0.8787 | 0.8665 |
| 54 | RM535 | 2 | 195.7 | 17 | 0.8687 | 0.8571 |  | 186 | RM8206 | 9 | 3.2 | 11 | 0.7441 | 0.7131 |
| 55 | RM132 | 3 | 3.9 | 6 | 0.4043 | 0.3748 |  | 187 | RM524 | 9 | 42.5 | 12 | 0.8334 | 0.813 |
| 56 | RM1332 | 3 | 11.5 | 4 | 0.6874 | 0.6251 |  | 188 | RM3912 | 9 | 46.3 | 10 | 0.8463 | 0.8268 |
| 57 | RM5849 | 3 | 18.4 | 15 | 0.8122 | 0.7898 |  | 189 | RM566 | 9 | 50.7 | 9 | 0.7515 | 0.714 |
| 58 | RM489 | 3 | 20.3 | 13 | 0.8928 | 0.8831 |  | 190 | RM434 | 9 | 57.7 | 10 | 0.8544 | 0.8384 |
| 59 | RM545 | 3 | 24.7 | 5 | 0.6118 | 0.5437 |  | 191 | RM3600 | 9 | 62.7 | 12 | 0.7971 | 0.7724 |
| 60 | RM5480 | 3 | 25.9 | 11 | 0.6356 | 0.6046 |  | 192 | RM24481 | 9 | 63 | 19 | 0.8966 | 0.8883 |
| 61 | RM3467 | 3 | 28.2 | 14 | 0.8923 | 0.8827 |  | 193 | RM3533 | 9 | 65.1 | 14 | 0.827 | 0.8057 |
| 62 | RM3766 | 3 | 34.8 | 10 | 0.7173 | 0.6876 |  | 194 | RM6570 | 9 | 68.2 | 9 | 0.5486 | 0.5225 |
| 63 | RM7 | 3 | 36.9 | 6 | 0.7178 | 0.6687 |  | 195 | RM410 | 9 | 79.3 | 9 | 0.7061 | 0.664 |
| 64 | RM5639 | 3 | 39.8 | 8 | 0.7272 | 0.6846 |  | 196 | RM257 | 9 | 79.7 | 11 | 0.7725 | 0.7489 |
| 65 | RM7197 | 3 | 44.4 | 11 | 0.8348 | 0.814 |  | 197 | RM201 | 9 | 81.2 | 9 | 0.7699 | 0.7358 |
| 66 | RM7345 | 3 | 48.8 | 7 | 0.6722 | 0.638 |  | 198 | OSR28 | 9 | 85.4 | 15 | 0.8596 | 0.8447 |
| 67 | RM282 | 3 | 55.8 | 11 | 0.8483 | 0.8306 |  | 199 | RM5384 | 9 | 90.7 | 10 | 0.8618 | 0.8464 |
| 68 | RM338 | 3 | 61.9 | 8 | 0.7434 | 0.7048 |  | 200 | RM1013 | 9 | 93.5 | 8 | 0.762 | 0.7266 |
| 69 | RM218 | 3 | 67.8 | 9 | 0.7143 | 0.6844 |  | 201 | RM7492 | 10 | 0 | 12 | 0.8683 | 0.8549 |
| 70 | RM232 | 3 | 76.7 | 13 | 0.8395 | 0.8218 |  | 202 | RM7545 | 10 | 7.6 | 25 | 0.942 | 0.9389 |
| 71 | RM7403 | 3 | 82.3 | 4 | 0.2271 | 0.2076 |  | 203 | RM6646 | 10 | 13.3 | 9 | 0.8151 | 0.7952 |
| 72 | RM6266 | 3 | 94.9 | 6 | 0.6558 | 0.612 |  | 204 | RM244 | 10 | 15 | 6 | 0.4863 | 0.4534 |
| 73 | RM7097 | 3 | 115.6 | 7 | 0.789 | 0.7576 |  | 205 | RM216 | 10 | 24.8 | 3 | 0.6003 | 0.5272 |
| 74 | RM135 | 3 | 120.4 | 9 | 0.7733 | 0.743 |  | 206 | RM311 | 10 | 25.2 | 8 | 0.7989 | 0.7721 |
| 75 | RM168 | 3 | 122.8 | 12 | 0.5901 | 0.564 |  | 207 | RM184 | 10 | 41.6 | 5 | 0.7572 | 0.7163 |
| 76 | RM186 | 3 | 127.4 | 7 | 0.7767 | 0.74 |  | 208 | RM1125 | 10 | 46.8 | 14 | 0.8477 | 0.8335 |
| 77 | RM16 | 3 | 131.5 | 7 | 0.3499 | 0.3187 |  | 209 | RM258 | 10 | 48.8 | 8 | 0.6832 | 0.6172 |
| 78 | RM5475 | 3 | 137.9 | 21 | 0.9091 | 0.9021 |  | 210 | RM5629 | 10 | 53.6 | 11 | 0.7842 | 0.7577 |
| 79 | RM416 | 3 | 140.1 | 9 | 0.7348 | 0.6973 |  | 211 | RM6100 | 10 | 53.9 | 5 | 0.314 | 0.2917 |
| 80 | RM6712 | 3 | 158.2 | 12 | 0.7876 | 0.7628 |  | 212 | RM1108 | 10 | 55.3 | 6 | 0.3226 | 0.3071 |
| 81 | RM448 | 3 | 191.6 | 13 | 0.8729 | 0.8603 |  | 213 | RM3773 | 10 | 58.9 | 17 | 0.8994 | 0.8908 |
| 82 | RM148 | 3 | 249.3 | 8 | 0.7374 | 0.6917 |  | 214 | RM269 | 10 | 69.6 | 5 | 0.6592 | 0.5958 |
| 83 | RM307 | 4 | 0 | 13 | 0.8486 | 0.8311 |  | 215 | RM5352 | 10 | 71.4 | 5 | 0.5324 | 0.4359 |
| 84 | RM335 | 4 | 5.4 | 16 | 0.8399 | 0.8213 |  | 216 | RM304 | 10 | 73 | 6 | 0.1444 | 0.1423 |
| 85 | RM518 | 4 | 7.9 | 10 | 0.7967 | 0.7683 |  | 217 | RM171 | 10 | 73 | 5 | 0.6438 | 0.5752 |
| 86 | RM3471 | 4 | 16.7 | 20 | 0.8901 | 0.8816 |  | 218 | RM6160 | 10 | 81 | 8 | 0.6045 | 0.5679 |
| 87 | RM4835 | 4 | 18.3 | 9 | 0.5341 | 0.4478 |  | 219 | RM590 | 10 | 83.3 | 10 | 0.8163 | 0.7915 |
| 88 | RM5687 | 4 | 25.4 | 9 | 0.767 | 0.7406 |  | 220 | RM333 | 10 | 110.4 | 7 | 0.3637 | 0.3515 |
| 89 | RM6314 | 4 | 41.5 | 16 | 0.828 | 0.8088 |  | 221 | RM286 | 11 | 0.1 | 8 | 0.7042 | 0.6736 |
| 90 | RM471 | 4 | 53.8 | 10 | 0.7822 | 0.7594 |  | 222 | RM6327 | 11 | 1.7 | 20 | 0.8915 | 0.8817 |
| 91 | RM5951 | 4 | 56.1 | 5 | 0.7031 | 0.6515 |  | 223 | RM1240 | 11 | 6.5 | 16 | 0.8597 | 0.8456 |
| 92 | RM142 | 4 | 60.2 | 16 | 0.8722 | 0.8601 |  | 224 | RM7557 | 11 | 9.2 | 5 | 0.5874 | 0.5371 |
| 93 | RM6997 | 4 | 62.1 | 12 | 0.7657 | 0.7397 |  | 225 | RM1812 | 11 | 10.3 | 10 | 0.6117 | 0.5532 |
| 94 | RM7563 | 4 | 68.3 | 8 | 0.8506 | 0.8334 |  | 226 | RM6544 | 11 | 19.8 | 5 | 0.4871 | 0.4505 |
| 95 | RM6114 | 4 | 72 | 12 | 0.8605 | 0.8459 |  | 227 | RM3133 | 11 | 32.7 | 5 | 0.687 | 0.6314 |
| 96 | RM6589 | 4 | 85.2 | 8 | 0.837 | 0.8166 |  | 228 | RM167 | 11 | 37.5 | 5 | 0.6592 | 0.5885 |
| 97 | RM317 | 4 | 96 | 6 | 0.7116 | 0.662 |  | 229 | RM3701 | 11 | 45.3 | 11 | 0.7292 | 0.6915 |
| 98 | RM6089 | 4 | 97.7 | 12 | 0.8392 | 0.8206 |  | 230 | RM7391 | 11 | 54.3 | 10 | 0.8255 | 0.8073 |
| 99 | RM3513 | 4 | 99.6 | 8 | 0.5394 | 0.4995 |  | 231 | RM7303 | 11 | 64.2 | 4 | 0.0845 | 0.0833 |
| 100 | RM3836 | 4 | 108.2 | 11 | 0.8428 | 0.8252 |  | 232 | RM7120 | 11 | 66.6 | 6 | 0.5972 | 0.5367 |
| 101 | RM280 | 4 | 128.9 | 3 | 0.388 | 0.338 |  | 233 | RM287 | 11 | 68.6 | 8 | 0.7654 | 0.7344 |
| 102 | RM559 | 4 | 129.6 | 6 | 0.725 | 0.6824 |  | 234 | RM457 | 11 | 78.8 | 8 | 0.5817 | 0.5365 |
| 103 | RM349 | 4 | 146.8 | 8 | 0.7285 | 0.6937 |  | 235 | RM5349 | 11 | 79.1 | 6 | 0.6801 | 0.6319 |
| 104 | RM348 | 4 | 160.8 | 5 | 0.5228 | 0.4653 |  | 236 | RM209 | 11 | 84.7 | 6 | 0.4316 | 0.3874 |
| 105 | RM1182 | 5 | 3 | 11 | 0.8551 | 0.8383 |  | 237 | RM21 | 11 | 85.7 | 11 | 0.7621 | 0.7262 |
| 106 | RM153 | 5 | 3 | 13 | 0.8381 | 0.8205 |  | 238 | RM7170 | 11 | 101.9 | 12 | 0.876 | 0.8634 |
| 107 | RM122 | 5 | 3 | 6 | 0.2254 | 0.2141 |  | 239 | RM206 | 11 | 102.9 | 15 | 0.8567 | 0.8414 |
| 108 | RM159 | 5 | 5.4 | 14 | 0.8933 | 0.8837 |  | 240 | RM7163 | 11 | 112.4 | 2 | 0.0829 | 0.0794 |
| 109 | RM267 | 5 | 25 | 12 | 0.858 | 0.8456 |  | 241 | RM6293 | 11 | 117.3 | 6 | 0.6658 | 0.6015 |
| 110 | RM437 | 5 | 31.5 | 2 | 0.2585 | 0.2251 |  | 242 | RM224 | 11 | 120.1 | 15 | 0.8913 | 0.8813 |
| 111 | RM3193 | 5 | 36.4 | 6 | 0.7561 | 0.7245 |  | 243 | RM20 | 12 | 3.2 | 13 | 0.8137 | 0.7894 |
| 112 | RM574 | 5 | 41 | 7 | 0.7719 | 0.7467 |  | 244 | RM19 | 12 | 20.9 | 15 | 0.8568 | 0.8411 |
| 113 | RM249 | 5 | 50.2 | 8 | 0.7255 | 0.6824 |  | 245 | RM247 | 12 | 26.7 | 11 | 0.8211 | 0.7986 |
| 114 | RM6082 | 5 | 53.5 | 15 | 0.8558 | 0.8433 |  | 246 | RM6296 | 12 | 26.7 | 5 | 0.5781 | 0.535 |
| 115 | RM598 | 5 | 62.7 | 6 | 0.5378 | 0.4843 |  | 247 | RM7619 | 12 | 38.1 | 3 | 0.4953 | 0.4067 |
| 116 | RM473B | 5 | 78.7 | 10 | 0.8589 | 0.8429 |  | 248 | RM512 | 12 | 39.4 | 6 | 0.3131 | 0.285 |
| 117 | RM164 | 5 | 91.4 | 8 | 0.66 | 0.6291 |  | 249 | RM5746 | 12 | 39.4 | 16 | 0.8518 | 0.8378 |
| 118 | RM188 | 5 | 95.3 | 12 | 0.8289 | 0.8097 |  | 250 | RM277 | 12 | 48.2 | 3 | 0.4164 | 0.3344 |
| 119 | RM161 | 5 | 96.9 | 18 | 0.9043 | 0.8965 |  | 251 | RM1337 | 12 | 51.5 | 9 | 0.7053 | 0.6585 |
| 120 | RM305 | 5 | 96.9 | 7 | 0.5781 | 0.5235 |  | 252 | RM511 | 12 | 59.8 | 11 | 0.8242 | 0.8021 |
| 121 | RM3170 | 5 | 115.4 | 14 | 0.8756 | 0.8632 |  | 253 | RM1246 | 12 | 65.3 | 11 | 0.7139 | 0.6881 |
| 122 | RM480 | 5 | 130.6 | 18 | 0.8778 | 0.8679 |  | 254 | RM7102 | 12 | 71.8 | 10 | 0.7733 | 0.7495 |
| 123 | RM5818 | 5 | 144.9 | 11 | 0.8314 | 0.8112 |  | 255 | RM309 | 12 | 73 | 10 | 0.8132 | 0.7888 |
| 124 | RM8109 | 6 | 1.7 | 11 | 0.7724 | 0.7433 |  | 256 | RM6869 | 12 | 75.8 | 15 | 0.8823 | 0.8711 |
| 125 | RM508 | 6 | 2.3 | 15 | 0.8389 | 0.821 |  | 257 | RM463 | 12 | 75.5 | 8 | 0.4325 | 0.4119 |
| 126 | RM510 | 6 | 11.5 | 12 | 0.8259 | 0.8108 |  | 258 | RM3331 | 12 | 89.5 | 9 | 0.7172 | 0.6692 |
| 127 | RM225 | 6 | 26.2 | 12 | 0.7548 | 0.7204 |  | 259 | RM270 | 12 | 91.3 | 9 | 0.7391 | 0.7022 |
| 128 | RM405 | 6 | 28.6 | 6 | 0.7279 | 0.6798 |  | 260 | RM5479 | 12 | 95.4 | 19 | 0.727 | 0.7061 |
| 129 | RM2126 | 6 | 32.7 | 12 | 0.7299 | 0.6904 |  | 261 | RM17 | 12 | 107.4 | 12 | 0.838 | 0.8207 |
| 130 | RM50 | 6 | 32.7 | 7 | 0.6934 | 0.6614 |  | 262 | RM12 | 12 | 107.4 | 4 | 0.5373 | 0.4561 |
| 131 | RM276 | 6 | 33.5 | 13 | 0.8605 | 0.8456 |  |  | Total Alleles | |  | 2754 |  |  |
| 132 | RM314 | 6 | 33.6 | 9 | 0.7793 | 0.7476 |  |  | Mean |  |  | 10.51 | 0.7331 | 0.7053 |
