# Supplementary material for: Genetic Diversity and Elite Allele Mining for Grain Traits in Rice (Oryza sativa L.) by Association Mapping
Source: Front Plant Sci. 2016 Jun 7;7:787. doi: 10.3389/fpls.2016.00787 (PMC4896222; doi:10.3389/fpls.2016.00787)
Supplement: Supplementary file 3 [file Table3.DOC]

Supplementary table S3 Marker-trait associations with *P*-value less than 0.05, proportion of phenotypic variance explained (PVE), marker position on chromosome derived from 262 markers and 628 rice accessions

| Trait | Marker | Chr. | Position (cM) | 2013 | | 2014 | |
| --- | --- | --- | --- | --- | --- | --- | --- |
|  |  |  |  | P value | PVE (%) | P value | PVE (%) |
| GL | RM486 | 1 | 147.9 | 1.02E-02 | 3.74 | 9.92E-03 | 3.75 |
|  | RM128 | 1 | 151.6 | 2.93E-02 | 4.38 | 2.84E-02 | 4.4 |
|  | RM297 | 1 | 161.3 | 1.19E-03 | 21.97 | 1.26E-03 | 21.89 |
|  | RM7288 | 2 | 42.4 | 4.47E-02 | 6.37 | 3.93E-02 | 6.47 |
|  | RM3766 | 3 | 34.8 | 4.09E-04 | 4.92 | 4.02E-04 | 4.93 |
|  | RM7097 | 3 | 115.6 | 4.11E-02 | 2.42 | 3.91E-02 | 2.45 |
|  | RM168 | 3 | 138.6 | 1.73E-02 | 3.76 | 1.68E-02 | 3.78 |
|  | RM335 | 4 | 0.8 | 7.77E-05 | 7.92 | 8.38E-05 | 7.88 |
|  | RM4835 | 4 | 18.3 | 1.93E-02 | 4.74 | 1.77E-02 | 4.81 |
|  | RM153 | 5 | 0.5 | 8.67E-04 | 6.43 | 8.26E-04 | 6.45 |
|  | RM161 | 5 | 96.9 | 1.49E-02 | 5.36 | 1.47E-02 | 5.37 |
|  | RM3330 | 6 | 48.5 | 4.22E-02 | 4.17 | 4.44E-02 | 4.13 |
|  | RM345 | 6 | 140.6 | 3.98E-05 | 5.22 | 4.03E-05 | 5.22 |
|  | RM82 | 7 | 41.6 | 1.78E-02 | 2.04 | 1.90E-02 | 2.01 |
|  | RM125 | 7 | 44.4 | 9.86E-03 | 2.6 | 9.78E-03 | 2.61 |
|  | RM6011 | 7 | 73.2 | 6.53E-03 | 17.38 | 6.32E-03 | 17.42 |
|  | RM336 | 7 | 80 | 3.70E-02 | 3.63 | 3.91E-02 | 3.6 |
|  | RM134 | 7 | 108 | 3.62E-02 | 3.13 | 4.01E-02 | 3.08 |
|  | RM6863 | 8 | 16.4 | 1.08E-02 | 3.81 | 1.23E-02 | 3.75 |
|  | RM6976 | 8 | 92.2 | 3.91E-04 | 8.42 | 4.09E-04 | 8.4 |
|  | RM524 | 9 | 31.5 | 1.28E-02 | 4.03 | 1.23E-02 | 4.05 |
|  | RM566 | 9 | 37.3 | 2.56E-02 | 3.07 | 2.27E-02 | 3.12 |
|  | RM3600 | 9 | 62.7 | 1.16E-04 | 6.75 | 1.19E-04 | 6.74 |
|  | RM201 | 9 | 84.3 | 8.33E-03 | 3.93 | 7.78E-03 | 3.97 |
|  | RM5384 | 9 | 91.5 | 2.54E-02 | 3.31 | 2.94E-02 | 3.23 |
|  | RM269 | 10 | 45.9 | 9.76E-03 | 3.15 | 9.25E-03 | 3.18 |
|  | RM3773 | 10 | 58.9 | 2.17E-02 | 4.91 | 1.85E-02 | 5 |
|  | RM6544 | 11 | 19.8 | 6.04E-03 | 2.75 | 6.73E-03 | 2.71 |
|  | RM1337 | 12 | 0 | 2.04E-03 | 5.05 | 2.16E-03 | 5.03 |
|  | RM20 | 12 | 0 | 3.85E-02 | 3.72 | 3.91E-02 | 3.71 |
|  | RM7102 | 12 | 51.8 | 2.36E-02 | 3.54 | 1.95E-02 | 3.64 |
|  | RM3331 | 12 | 89.5 | 1.25E-03 | 4.52 | 1.35E-03 | 4.48 |
|  |  |  |  |  |  |  |  |
| GW | RM1 | 1 | 31.7 | 5.28E-04 | 8.33 | 4.75E-03 | 6.05 |
|  |  |  |  |  |  |  |  |
| GT | RM84 | 1 | 18.8 | 2.16E-04 | 19.16 | 1.03E-06 | 23.46 |
|  | RM283 | 1 | 36.7 | 2.37E-04 | 5.76 | 1.92E-05 | 4.87 |
|  | RM3453 | 1 | 25.4 | 3.64E-04 | 9.18 | 5.73E-05 | 10.16 |
|  | RM1 | 1 | 31.7 | 8.12E-04 | 5.09 | 5.07E-04 | 5.23 |
|  | RM259 | 1 | 66.3 | 1.54E-03 | 6.5 | 8.22E-04 | 4.66 |
|  | RM583 | 1 | 43.2 | 2.56E-03 | 5.68 | 9.50E-04 | 5.2 |
|  | RM490 | 1 | 40.7 | 2.70E-03 | 2.99 | 1.03E-03 | 6.69 |
|  | RM8095 | 1 | 60.6 | 8.33E-03 | 3.66 | 2.16E-03 | 3.01 |
|  | RM140 | 1 | 60.1 | 8.68E-03 | 4.42 | 7.44E-03 | 4.2 |
|  | RM562 | 1 | 99.2 | 1.09E-02 | 3.37 | 7.53E-03 | 4 |
|  | RM9 | 1 | 121.7 | 1.27E-02 | 2.31 | 7.70E-03 | 3.85 |
|  | RM129 | 1 | 103.9 | 1.35E-02 | 15.72 | 1.27E-02 | 16.31 |
|  | RM5 | 1 | 117.8 | 1.50E-02 | 2.56 | 1.34E-02 | 3.26 |
|  | RM1231 | 1 | 123.2 | 2.36E-02 | 3.93 | 1.45E-02 | 3.27 |
|  | RM128 | 1 | 151.6 | 2.62E-02 | 4.18 | 2.21E-02 | 4.52 |
|  | RM297 | 1 | 161.3 | 2.69E-02 | 3.55 | 2.21E-02 | 4.3 |
|  | RM246 | 1 | 134.6 | 2.98E-02 | 3.13 | 2.32E-02 | 3.89 |
|  | RM212 | 1 | 163.1 | 3.22E-02 | 2.8 | 2.33E-02 | 5.42 |
|  | RM5389 | 1 | 142.4 | 3.38E-02 | 3.88 | 2.47E-02 | 2.39 |
|  | RM486 | 1 | 147.9 | 3.40E-02 | 2.14 | 3.69E-02 | 2.86 |
|  | RM265 | 1 | 170 | 3.72E-02 | 1.15 | 3.72E-02 | 3.09 |
|  | RM3482 | 1 | 157.6 | 3.88E-02 | 3.51 | 3.80E-02 | 2.17 |
|  | RM6831 | 1 | 213 | 5.00E-02 | 4.39 | 3.89E-02 | 3.85 |
|  |  |  |  |  |  |  |  |
| GL/GW | RM1 | 1 | 31.7 | 2.03E-02 | 5.28 | 2.33E-04 | 7.36 |
|  | RM297 | 1 | 161.3 | 5.35E-04 | 19.37 | 2.28E-03 | 18.47 |
|  | RM486 | 1 | 147.9 | 3.70E-02 | 3.09 | 4.43E-03 | 3.63 |
|  | RM7288 | 2 | 42.4 | 2.88E-03 | 8.3 | 2.24E-02 | 5.21 |
|  | RM300 | 2 | 55.1 | 3.51E-02 | 4.26 | 4.26E-02 | 2.96 |
|  |  |  |  |  |  |  |  |
| TGW | RM259 | 1 | 66.3 | 7.04E-03 | 5.4 | 3.08E-02 | 5.88 |
