# Supplementary material for: Genetic Diversity and Elite Allele Mining for Grain Traits in Rice (Oryza sativa L.) by Association Mapping
Source: Front Plant Sci. 2016 Jun 7;7:787. doi: 10.3389/fpls.2016.00787 (PMC4896222; doi:10.3389/fpls.2016.00787)
Supplement: Supplementary file 4 [file Table4.DOC]

Supplementary table S4 Positive elite alleles, phenotypic effect value and typical carrier materials for grain traits

| Trait | Locus-allele | Phenotypic effect value | | | Carrier variety |
| --- | --- | --- | --- | --- | --- |
|  |  | 2013 | 2014 | Mean |  |
| GL | RM297-145 | 0.24 | 0.19 | 0.22 | Tongjing109 |
|  | RM7288-110 | 0.79 | 0.83 | 0.81 | Cungu |
|  | RM7288-170 | 0.34 | 0.38 | 0.36 | Haobuka |
|  | RM7288-130 | 0.33 | 0.35 | 0.34 | Yuedao84 |
|  | RM7288-145 | 0.21 | 0.25 | 0.23 | Yuedao13 |
|  | RM335-160 | 0.69 | 0.73 | 0.71 | Sanjiang2 |
|  | RM335-140 | 0.45 | 0.47 | 0.46 | Huanghuazhan |
|  | RM335-180 | 0.23 | 0.28 | 0.26 | Kendao12 |
|  | RM153-200 | 0.44 | 0.45 | 0.45 | Yuedao41 |
|  | RM153-220 | 0.17 | 0.21 | 0.19 | Yuzhenxiang |
|  | RM161-110 | 1.77 | 1.82 | 1.8 | Yuedao62 |
|  | RM161-145 | 0.71 | 0.76 | 0.74 | Yuedao37 |
|  | RM161-125 | 0.51 | 0.56 | 0.54 | Fuyu3 |
|  | RM161-135 | 0.32 | 0.37 | 0.35 | Yuedao17 |
|  | RM161-180 | 0.25 | 0.27 | 0.26 | Yuedao72 |
|  | RM345-165 | 0.95 | 0.97 | 0.96 | Yuedao62 |
|  | RM345-155 | 0.67 | 0.72 | 0.7 | Sanjiang2 |
|  | RM345-105 | 0.24 | 0.28 | 0.26 | Maozitou |
|  | RM6011-150 | 1.21 | 1.25 | 1.23 | Yuedao41 |
|  | RM6011-145 | 0.21 | 1.25 | 0.73 | Yuedao13 |
|  | RM6976-135 | 1.75 | 1.74 | 1.75 | Yuedao62 |
|  | RM6976-210 | 0.81 | 0.86 | 0.84 | Yuedao85 |
|  | RM6976-155 | 0.6 | 0.65 | 0.63 | Yuedao13 |
|  | RM6976-195 | 0.38 | 0.43 | 0.41 | Katena |
|  | RM3600-120 | 1.78 | 1.83 | 1.81 | Yuedao62 |
|  | RM3600-170 | 1.17 | 1.22 | 1.2 | Yuedao88 |
|  | RM3600-180 | 0.61 | 0.66 | 0.64 | Yuedao113 |
|  | RM1337-145 | 0.85 | 0.88 | 0.87 | Yuedao41 |
|  | RM1337-170 | 0.47 | 0.51 | 0.49 | Yuedao51 |
|  |  |  |  |  |  |
| GW | RM1-120 | 0.19 | 0.18 | 0.19 | Ningjing1R-40 |
|  | RM1-90 | 0.19 | 0.21 | 0.2 | Wumangzaodao |
|  |  |  |  |  |  |
| GT | RM84-105 | 0.15 | 0.11 | 0.13 | Huaidao8 |
|  | RM3453-135 | 0.34 | 0.28 | 0.31 | Zhen9424 |
|  | RM3453-175 | 0.14 | 0.13 | 0.14 | Ningjing1R-122 |
|  | RM1-120 | 0.19 | 0.19 | 0.19 | Ningjing1R-37 |
|  | RM283-170 | 0.16 | 0.15 | 0.16 | Ningjing1R-122 |
|  | RM583-200 | 0.17 | 0.17 | 0.17 | Zhendao99 |
|  | RM259-190 | 0.17 | 0.16 | 0.17 | Ningjing1R-61 |
|  | RM259-175 | 0.11 | 0.14 | 0.13 | Zhendao99 |
|  | RM129-200 | 0.17 | 0.16 | 0.17 | Ningjing1R-122 |
|  | RM129-175 | 0.11 | 0.09 | 0.1 | Si4364 |
|  |  |  |  |  |  |
| GL/GW | RM1-170 | 0.91 | 0.93 | 0.92 | Yuedao100 |
|  | RM1-105 | 0.27 | 0.29 | 0.28 | Yuedao12 |
|  | RM297-145 | 0.22 | 0.15 | 0.19 | Zijianjingnuo |
|  | RM7288-130 | 0.25 | 0.27 | 0.26 | Yuedao89 |
|  | RM7288-145 | 0.19 | 0.21 | 0.2 | Yuedao12 |
|  |  |  |  |  |  |
| TGW | RM259-185 | 0.77 | 0.7 | 0.74 | Yuedao86 |
|  | RM259-155 | 0.46 | 0.71 | 0.59 | Haobuka |
|  | RM259-165 | 0.48 | 0.44 | 0.46 | Yuedao45 |
