# Supplementary material for: Genetic Diversity and Elite Allele Mining for Grain Traits in Rice (Oryza sativa L.) by Association Mapping
Source: Front Plant Sci. 2016 Jun 7;7:787. doi: 10.3389/fpls.2016.00787 (PMC4896222; doi:10.3389/fpls.2016.00787)
Supplement: Supplementary file 5 [file Table5.DOC]

Supplementary table S5 Elite alleles carried by the superior parents for grain traits and corresponding phenotypic effect

| Trait | Super parent | Locus-allele (Corresponding phenotypic effect value) |
| --- | --- | --- |
| Grain length | Yuedao 62 | RM153-200 (0.45), RM161-110 (1.80), RM345-165 (0.96), RM6976-135 (1.75), RM3600-120 (1.81) |
|  | Yuedao 85 | RM7288-130 (0.34), RM153-200 (0.45), RM6011-150 (1.23), RM345-165 (0.96), RM6976-210 (0.84), RM3600-170 (1.20), RM335-160 (0.71), RM1337-145 (0.87) |
|  | Yuedao 88 | RM153-200 (0.45), RM6011-150 (1.23), RM345-165 (0.96), RM6976-155 (0.63), RM3600-170 (1.20), RM335-160 (0.71), RM1337-145 (0.87) |
|  | Yuedao 113 | RM153-200 (0.45), RM6011-150 (1.23), RM345-165 (0.96), RM6976-135 (1.75), RM161-180 (0.26), RM3600-180 (0.64), RM335-160 (0.71), RM1337-145 (0.87) |
| Grain thickness | Zhen 9424 | RM3453-135 (0.31), RM583-200 (0.17) |
|  | Ningjing1R-37 | RM1-120 (0.19), RM283-170 (0.16), RM129-200 (0.17) |
|  | Ningjing1R-61 | RM283-170 (0.16), RM259-190 (0.17), RM129-200 (0.17) |
|  | Zhendao 99 | RM583-200 (0.17), RM259-175 (0.13) |
| Grain length to grain width ratio | Yuedao 12 | RM1-105 (0.28), RM7288-145 (0.20) |
|  | Yuedao 100 | RM1-170 (0.92) |
|  | Yuedao 89 | RM1-170 (0.92), RM7288-130 (0.26) |
