# Supplementary material for: Genetic Diversity and Elite Allele Mining for Grain Traits in Rice (Oryza sativa L.) by Association Mapping
Source: Front Plant Sci. 2016 Jun 7;7:787. doi: 10.3389/fpls.2016.00787 (PMC4896222; doi:10.3389/fpls.2016.00787)
Supplement: Supplementary file 6 [file Table6.DOC]

Supplementary table S6 The list for QTLs identified from this study and shared in previous studies

| Traits | SSR  markers | Chromosome | Start position  /bp^a^ | End position  /bp^a^ | QTL reported in the previous studies | | |
| --- | --- | --- | --- | --- | --- | --- | --- |
|  |  |  |  |  | Start position  /bp^a^ | End position /bp^a^ | Reference |
| Grain length | RM7288 | 2 | 9,033,547 | 9,033,882 | 9,033,547 | 9,033,882 | Tran Thi et al. (2014) |
|  | RM335 | 4 | 688,353 | 688,466 | 688,353 | 688,466 | Dang et al. (2015) |
|  | RM345 | 6 | 30,864,845 | 30,864,999 | 24,035,491 | 31,206,401 | Aluko et al. (2004) |
|  | RM6011 | 7 | 20,786,437 | 20,786,575 | 19,745,179 | 20,914,347 | Huang et al. (2011) |
|  | RM6976 | 8 | 23,555,534 | 23,555,817 | 21,427,234 | 26,700,842 | Agrama et al. (2007) |
|  | RM3600 | 9 | 17,107,752 | 17,107,843 | 17,107,752 | 17,107,843 | Tran Thi et al. (2014) |
| 1000-grain weight | RM259 |  | 7,445,627 | 7,445,919 | 7,270,403 | 16,486,411 | Lin et al. (1996) |
|  |  |  |  |  | 4,753,787 | 16,486,442 | Hua et al. (2002); Xing et al. (2002) |
